# Exposure of Endothelium to Biomimetic Flow Waveforms Yields Identification of miR-199a-5p as a Potent Regulator of Arteriogenesis

**DOI:** 10.1016/j.omtn.2018.08.001

**Published:** 2018-08-08

**Authors:** Joshua L. Heuslein, Catherine M. Gorick, Stephanie P. McDonnell, Ji Song, Brian H. Annex, Richard J. Price

**Affiliations:** 1 Department of Biomedical Engineering, University of Virginia, Charlottesville, VA, USA; 2 Robert M. Berne Cardiovascular Research Center, University of Virginia, Charlottesville, VA, USA

**Keywords:** endothelial, microRNA, miR-199a-5p, peripheral arterial disease, femoral arterial ligation, hindlimb ischemia, shear stress

## Abstract

Arteriogenesis, the growth of endogenous collateral arteries bypassing arterial occlusion(s), is a fundamental shear stress-induced adaptation with implications for treating peripheral arterial disease (PAD). Nonetheless, endothelial mechano-signaling during arteriogenesis is incompletely understood. Here we tested the hypothesis that a mechanosensitive microRNA, miR-199a-5p, regulates perfusion recovery and collateral arteriogenesis following femoral arterial ligation (FAL) via control of monocyte recruitment and pro-arteriogenic gene expression. We have previously shown that collateral artery segments exhibit distinctly amplified arteriogenesis if they are exposed to reversed flow following FAL in the mouse. We performed a genome-wide analysis of endothelial cells exposed to a biomimetic reversed flow waveform. From this analysis, we identified mechanosensitive miR-199a-5p as a novel candidate regulator of collateral arteriogenesis. *In vitro*, miR-199a-5p inhibited pro-arteriogenic gene expression (IKKβ, Cav1) and monocyte adhesion to endothelium. *In vivo*, following FAL in mice, miR-199a-5p overexpression impaired foot perfusion and arteriogenesis. In contrast, a single intramuscular anti-miR-199a-5p injection elicited a robust therapeutic response, including complete foot perfusion recovery, markedly augmented arteriogenesis (>3.4-fold increase in segment conductance), and improved gastrocnemius tissue composition. Finally, we found plasma miR-199a-5p to be elevated in human PAD patients with intermittent claudication compared to a risk factor control population. Through our transformative analysis of endothelial mechano-signaling in response to a biomimetic amplified arteriogenesis flow waveform, we have identified miR-199a-5p as both a potent regulator of arteriogenesis and a putative target for treating PAD.

## Graphical Abstract

**Figure:**
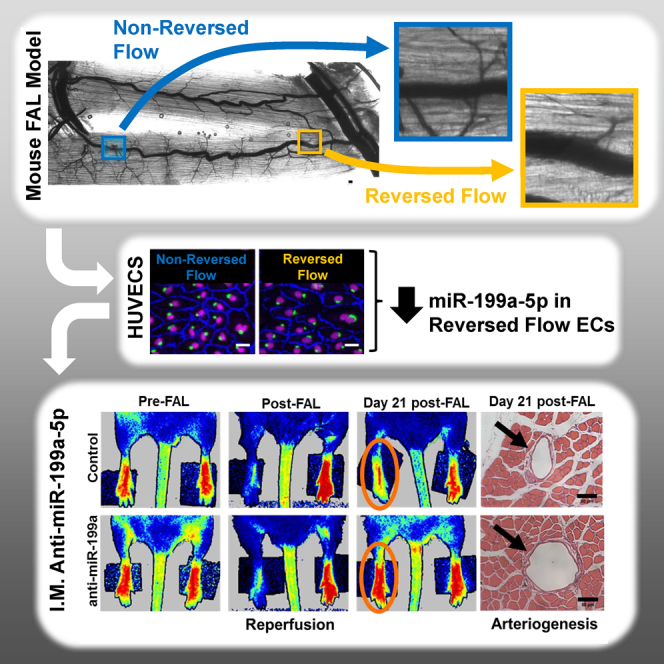

## Introduction

Peripheral arterial disease (PAD) has become a global problem: it is estimated that over 202 million people worldwide have PAD.^1^ PAD arises when atherosclerotic plaques block arteries in the lower limbs, thereby limiting blood flow to the distal tissue, ultimately leading to intermittent claudication or critical limb ischemia in severe cases. Many PAD patients are either not amenable to surgical intervention or receive little long-term benefit from surgery.^2^ Revascularization strategies to stimulate the growth of new capillaries from preexisting vessels (i.e., angiogenesis) or lumenal expansion of pre-existing arteries (i.e., arteriogenesis) remain promising therapeutic options, despite their limited success to date.^3^ The stimulation of angiogenesis is important in PAD, as capillary density is reduced in these patients;^2^, ^4^, ^5^ however, it is also imperative to restore the driving pressure to the distal tissue via lumenal expansion (i.e., arteriogenesis) of collateral arteries bypassing the occlusion(s).^6^, ^7^, ^8^ Arteriogenesis is well known to be stimulated by altered shear stress,^9^ though endothelial mechano-signaling in response to this altered shear stress is incompletely understood.

**Figure 1:**
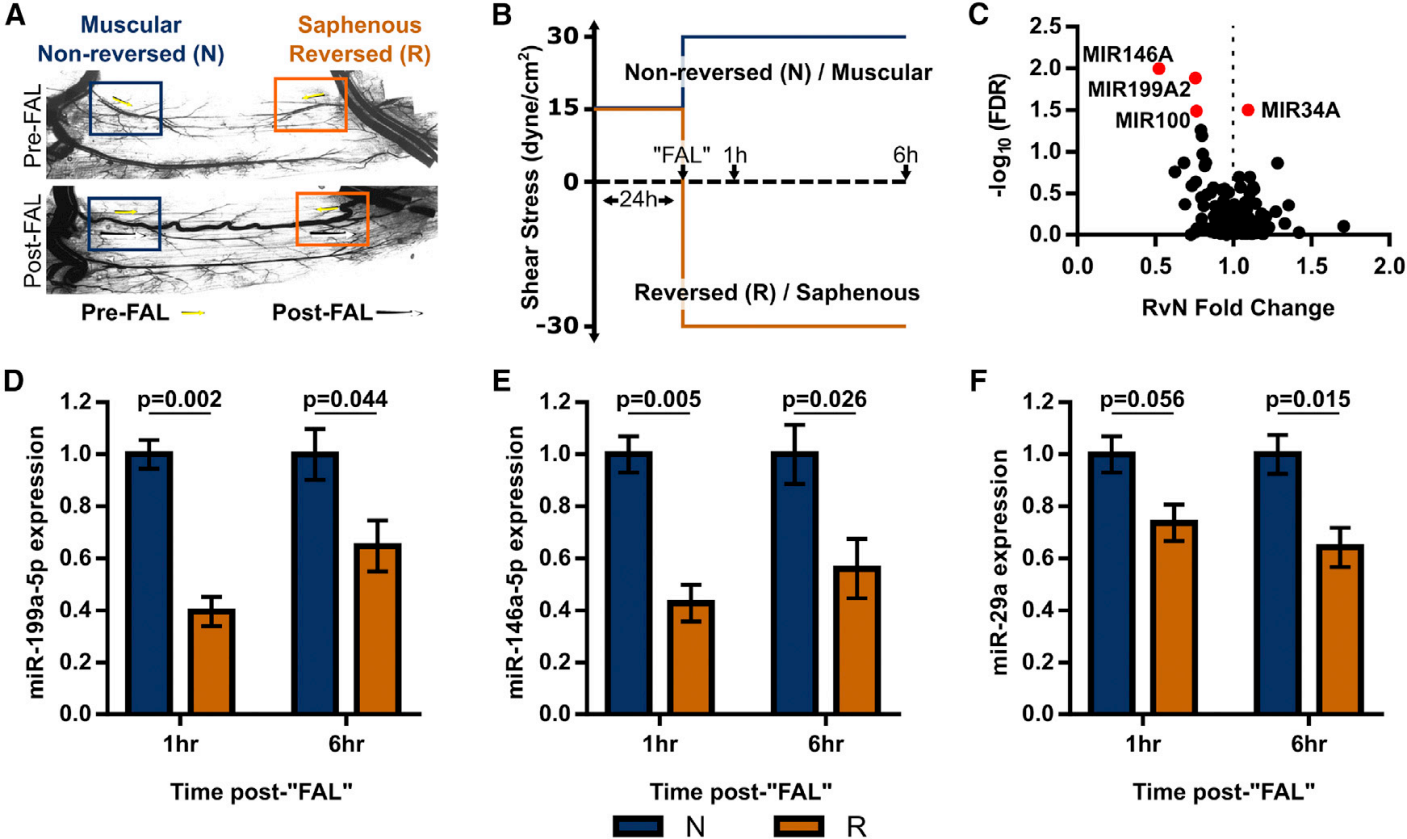
Endothelial Cell miRNA Expression Is Differentially Regulated by Shear Stress Waveforms Biomimetic of Collateral Artery Segments Exhibiting Varied Arteriogenic Responses (A) Representative vascular cast images of gracilis adductor collaterals in BALB/c mice taken from unligated sham control (top) and day 21 post-femoral arterial ligation (FAL, bottom) groups. Arrows indicate direction and magnitude of blood flow pre- (yellow) and post- (white) FAL. The femoral artery is ligated just distal to the epigastric artery such that some collateral segments (muscular) experience a 2-fold increase in shear stress magnitude (non-reversed flow), while other segments (saphenous) are exposed to both a 2-fold increase in shear stress magnitude and reversed flow direction (reversed flow). Arteriogenesis is amplified in the flow-reversed, saphenous regions of collateral arteries. (B) Schematic depicting biomimetic waveforms applied to HUVECs to simulate saphenous (reversed flow, R) and muscular (non-reversed flow, N) regions. (C) Volcano plot of all microRNAs (miRNAs) on the Affymetrix ST 1.0 human microarray dataset (n = 4) from Heuslein et al.^9^ HUVECs were exposed to the flow waveforms in (B). Gene expression was determined at 6 hr after simulated FAL. FDR, false discovery rate. Red dots indicate miRNAs with FDR < 0.05. (D–F) Bar graphs of mature miR-199a-5p (D), miR-146a-5p (E), and miR-29a (F) expression in HUVECs exposed to biomimetic shear stress conditions from (B) (n = 3–5). Student’s t test. Data are mean ± SEM.

We recently reported that a subset of collateral segments in the mouse hindlimb display remarkably permanent, amplified arteriogenesis after femoral arterial ligation (FAL).^10^ Specifically, we showed that collateral artery segments exposed to both a 2-fold increase in shear stress magnitude and reversed flow direction (reversed flow) following FAL exhibit an ∼30% increase in lumenal diameter 12 weeks post-FAL compared to segments experiencing just a 2-fold increase in shear stress magnitude (non-reversed flow) (Figure 1A).^11^ Moreover, by applying shear stress waveforms biomimetic of these *in vivo* hemodynamics to endothelial cells (ECs) *in vitro*, we were able to generate a direct, comprehensive mapping of EC mechanosensitive signaling to differential arteriogenesis responses.^11^ Comparative analysis of EC mechano-signaling corresponding to these differential responses may enable the discovery of novel regulators of arteriogenesis.

To this end, mature microRNAs (miRNAs) are now well recognized as key regulators of vascular remodeling.^12^, ^13^ miRNAs are ∼22-nt, non-coding RNAs that are endogenous regulators of gene expression.^14^ Mature miRNA incorporates into the RNA-induced silencing complex (RISC) and then binds to a target mRNA, usually in the 3′ UTR of the mRNA.^15^ miRNA binding acts to suppress target gene expression by inhibiting mRNA translation to protein or by promoting mRNA degradation,^16^ depending on miRNA-target complementarity.^17^ Additionally, miRNAs are attractive as potential therapeutic targets as they are short, highly conserved, and can negatively regulate gene expression of multiple mRNA targets.^18^ Several miRNAs have been implicated as regulators of vascular growth, primarily angiogenesis in ischemic tissue, in response to arterial occlusion.^19^, ^20^, ^21^, ^22^, ^23^, ^24^, ^25^, ^26^, ^27^ However, the explicit role of mechanosensitive miRNAs in collateral arteriogenesis is poorly understood. Here, using a genome-wide approach on cultured ECs exposed to flow waveforms biomimetic of collateral segments exhibiting moderate and amplified arteriogenesis *in vivo*, we first identified mechanosensitive miRNA-199a as a potential regulator of collateral arteriogenesis. We then tested the hypothesis that mechanosensitive miRNA-199a regulates perfusion recovery and collateral arteriogenesis following FAL via the control of monocyte recruitment and pro-arteriogenic gene expression.

## Results

### EC miRNA Expression Is Regulated by Shear Stress Waveforms Biomimetic of Collateral Artery Segments Exhibiting Differential Arteriogenic Responses

To identify candidate miRNAs, we examined previously generated genome-wide cDNA microarray data (GEO: GSE46248)^10^ from ECs exposed to shear stress waveforms biomimetic of those measured *in vivo*^11^ in collateral artery segments exhibiting either moderate (muscular) or amplified (saphenous) arteriogenesis responses (Figure 1A). Briefly, human umbilical vein ECs (HUVECs) were preconditioned for 24 hr at a baseline arterial shear stress (15 dynes/cm^2^)^28^ to establish EC alignment, planar cell polarity, and steady-state signaling, thereby mimicking the *in vivo* baseline state. A FAL was then simulated by a stepwise 100% increase in shear stress, in either the same direction or in the opposite direction, to mimic shear stress changes occurring in the muscular branch (non-reversed flow) and saphenous artery (reversed flow) entrance regions, respectively (Figures 1A and 1B). At 6 hr after our simulated FAL, we isolated total RNA and performed genome-wide analysis using Affymetrix cDNA 1.0 ST microarrays. Genome-wide analysis identified a small subset of miRNA genes differentially regulated between reversed and non-reversed flow conditions (Figure 1C; Table S1). The expression of several candidate miRNAs (miR-199a-5p, miR-146a-5p, and miR-29a) was assessed by qRT-PCR both at 1 and 6 hr after simulated FAL to confirm microarray results. We found all these candidate miRNAs to be significantly downregulated (∼40%) in HUVECs exposed to the reversed flow waveform 6 hr after simulated FAL (Figures 1D–1F).

### miRNA-199a Negatively Regulates Pro-arteriogenic Endothelial Gene Expression and Monocyte Adhesion to Flow-Exposed ECs *In Vitro*

Of these candidate miRNAs, miR-199a-5p (henceforth denoted as miR-199a) was of particular interest as it is known to regulate mRNAs of several pathways necessary for arteriogenesis, including inhibitor of nuclear factor kappa-B kinases subunit beta (IKKβ)^29^ and caveolin-1 (Cav1).^30^, ^31^ We therefore sought to determine if miR-199a regulates the expression of these pro-arteriogenic genes in ECs exposed to the biomimetic shear stress waveforms. HUVECs were transfected with 20 nM miR-199a mimic, anti-miR-199a locked nucleic acid oligonucleotides, or respective scramble controls, 24 hr prior to shear stress exposure. At 6 hr after simulated FAL, in scramble-transfected cells, the application of a reversed flow waveform resulted in an ∼50% reduction in miR-199a expression compared to non-reversed flow conditions (Figure S2), similar to previous results seen in Figure 1D. Transfection with miR-199a mimics increased relative miR-199a expression >60-fold (Figure S1A), while anti-miR-199a reduced miR-199a expression by ∼80% (Figure S1B), compared to their respective scramble controls in HUVECs exposed to the biomimetic waveforms. Overexpression of miR-199a significantly decreased the relative mRNA expression of IKKβ in HUVECs exposed to reversed flow conditions, while there was only a trend toward decreased IKKβ at the protein level (Figure S2). Inhibition of miR-199a did, however, lead to a significant increase in IKKβ expression at both the mRNA and protein levels under reversed flow conditions (Figure S2). Interestingly, Cav1 expression was significantly altered only at the protein level with miR-199a modulation, suggesting miR-199a targets Cav1 by inhibiting translation in this context. Cav1 protein expression was decreased with miR-199a overexpression, whereas it was increased with miR-199a inhibition in HUVECs exposed to a reversed flow (pro-arteriogenic) waveform (Figure S3).

**Figure 2:**
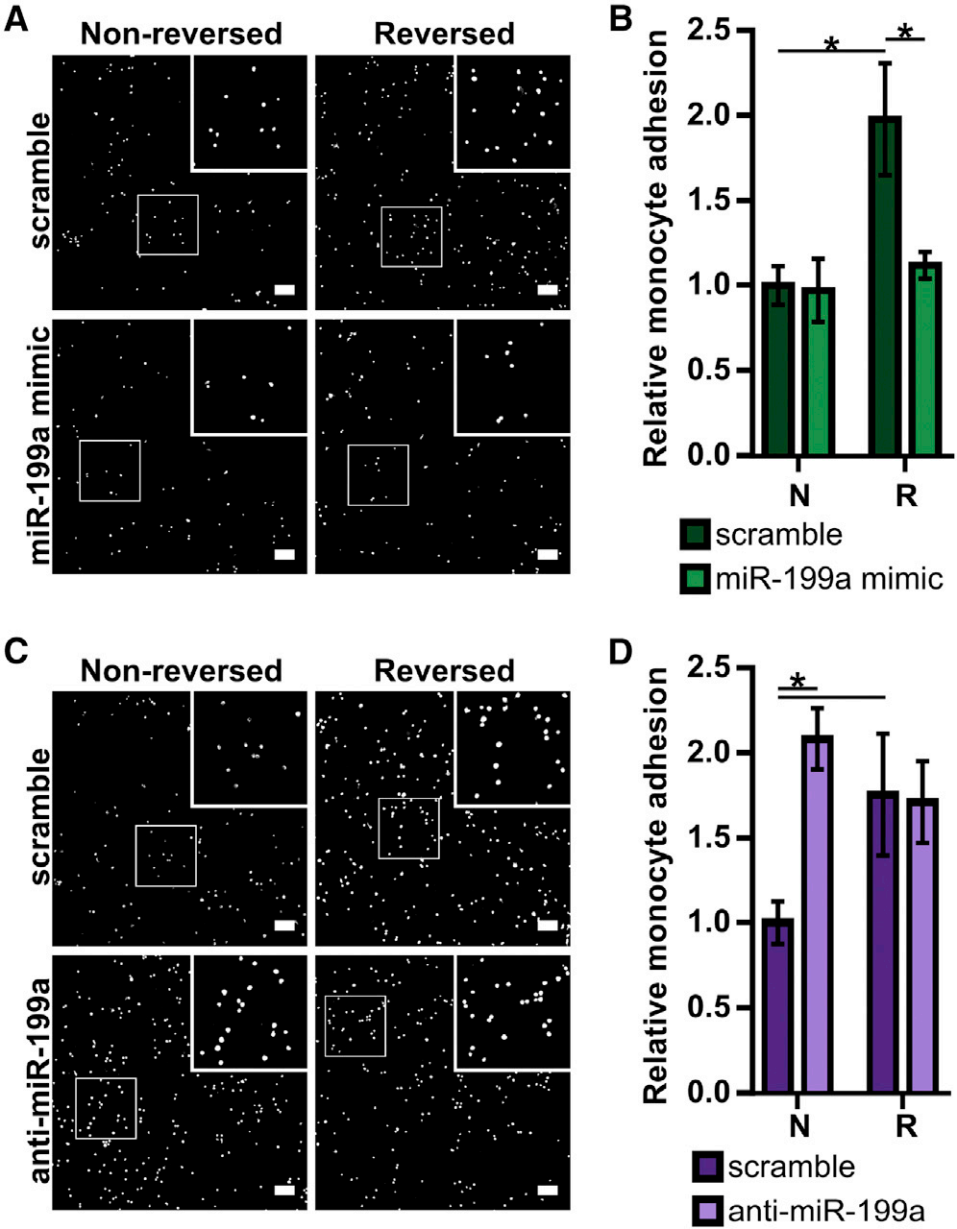
Monocyte Adhesion to HUVECs Exposed to Shear Stress Waveforms Biomimetic of Arteriogenic Collaterals Is Modulated by miR-199a (A) Representative confocal microscopy images of fluorescently labeled THP-1 monocytes adhered to HUVECs transfected with miR-199a or scrambled mimic, then subjected to non-reversed (N) or reversed (R) flow waveforms outlined in Figure 1B, 6 hr after simulated FAL (scale bar, 100 μm). Insets are the magnified 300 × 300-μm regions outlined by white boxes. (B) Bar graph quantifying relative number of adhered monocytes in each condition (n = 4). *p < 0.05, two-way ANOVA followed by a Holm-Sidak multiple comparisons test. (C) Representative images of fluorescently labeled THP-1 monocytes adhered to flow-exposed HUVECs transfected with anti-miR-199a or scrambled locked nucleic acid oligonucleotides, 6 hr after simulated FAL (scale bar, 100 μm). (D) Bar graph quantifying relative number of adhered monocytes in each condition (n = 4). *p < 0.05, two-way ANOVA followed by a Holm-Sidak multiple comparisons test. Data are mean ± SEM.

Monocyte adhesion to activated endothelium is a required step in the arteriogenesis cascade.^32^, ^33^, ^34^, ^35^, ^36^, ^37^ We sought to determine whether altered miR-199a expression modulates this critical process. In control conditions, HUVECs exposed to the reversed flow waveform experienced increased monocyte adhesion compared to those exposed to the non-reversed waveform (Figure 2), consistent with previous results.^10^ Overexpression of miR-199a attenuated this enhanced functional response, leading to a 44% decrease in monocyte adhesion in reversed flow conditions (Figures 2A and 2B). In non-reversed flow conditions, miR-199a overexpression did not further reduce monocyte adhesion. Conversely, the inhibition of miR-199a led to enhanced pro-arteriogenic function *in vitro*, as seen by a 90% increase in monocyte adhesion to non-reversed flow-exposed HUVECs, while there was no further increase with reversed flow (Figures 2C and 2D).

### Overexpression of miR-199a Limits Foot Reperfusion following FAL, while miR-199a Inhibition Elicits Complete Perfusion Recovery in BALB/c Mice

**Figure 3:**
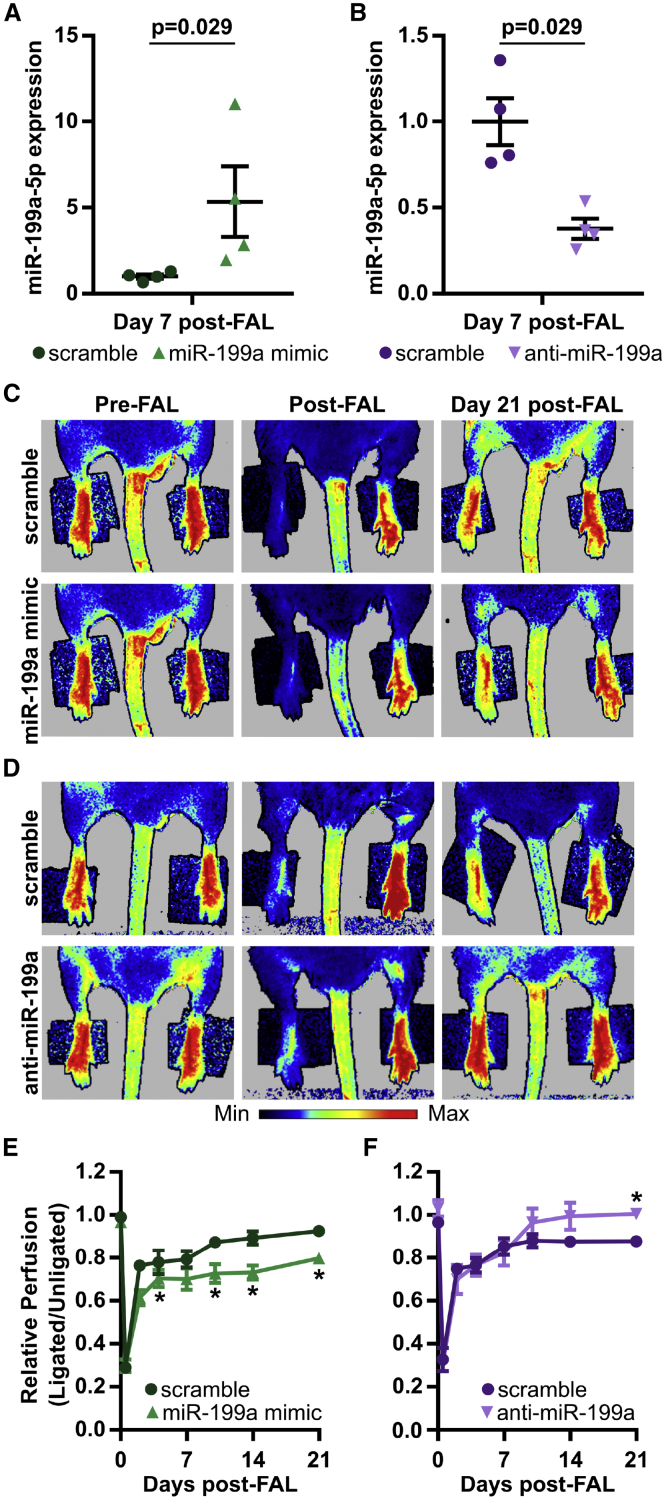
Overexpression of miR-199a Limits Foot Reperfusion following FAL, while miR-199a Inhibition Leads to Complete Perfusion Recovery in BALB/c Mice (A and B) Relative miR-199a-5p expression in the gracilis muscle 7 days post-FAL in BALB/c mice. Mice were treated with a single intramuscular (i.m.) injection of 7.5 nmol miR-199a mimic (A), anti-miR-199a (B), or respective scramble oligonucleotides immediately post-FAL (n = 4). Mann-Whitney U test. (C and D) Post-FAL foot perfusion recovery as assessed by laser Doppler perfusion imaging (n = 6) for miR-199a mimic (C) and anti-miR-199a (D) treated mice. (E and F) Line graphs of perfusion recovery in miR-199a mimic (E) and anti-miR-199a (F) treated mice. *p < 0.05 versus scramble, two-way ANOVA with repeated measures followed by Holm-Sidak test for multiple comparisons. Data are mean ± SEM.

To test the hypothesis that miR-199a regulates perfusion recovery and/or arteriogenesis *in vivo*, we performed FALs on BALB/c mice, and we modulated miR-199a expression via intramuscular injection of miR-199a mimic, anti-miR-199a locked nucleic acid oligonucleotides, or respective scramble controls directly into the gracilis muscle immediately following FAL. Local intramuscular injection of miR-199a mimic led to an ∼5-fold increase in relative miR-199a expression (Figure 3A), while anti-miR-199a decreased miR-199a expression ∼2.6-fold in the gracilis muscle 7 days post-FAL (Figure 3B). Additionally, we found that miR-199a overexpression decreased Cav1 protein expression and anti-miR-199a increased Cav1 protein expression compared to controls (Figure S4). Perfusion measurements of the plantar surface of the foot indicated moderate ischemia immediately post-FAL, followed by an incomplete perfusion recovery in the scramble-treated mice for both the overexpression (0.92 ± 0.02, day 21 post-FAL) and inhibition (0.88 ± 0.02, day 21 post-FAL) studies (Figures 3C–3F). When miR-199a was overexpressed, the reperfusion response was further dampened as early as 4 days post-FAL and only reached 80% reperfusion 21 days post-FAL (Figures 3C and 3E). However, anti-miR-199a-treated mice fully recovered foot perfusion by day 21 post-FAL (Figures 3D and 3F).

### Overexpression of miR-199a Inhibits Arteriogenesis in BALB/c Mice

**Figure 4:**
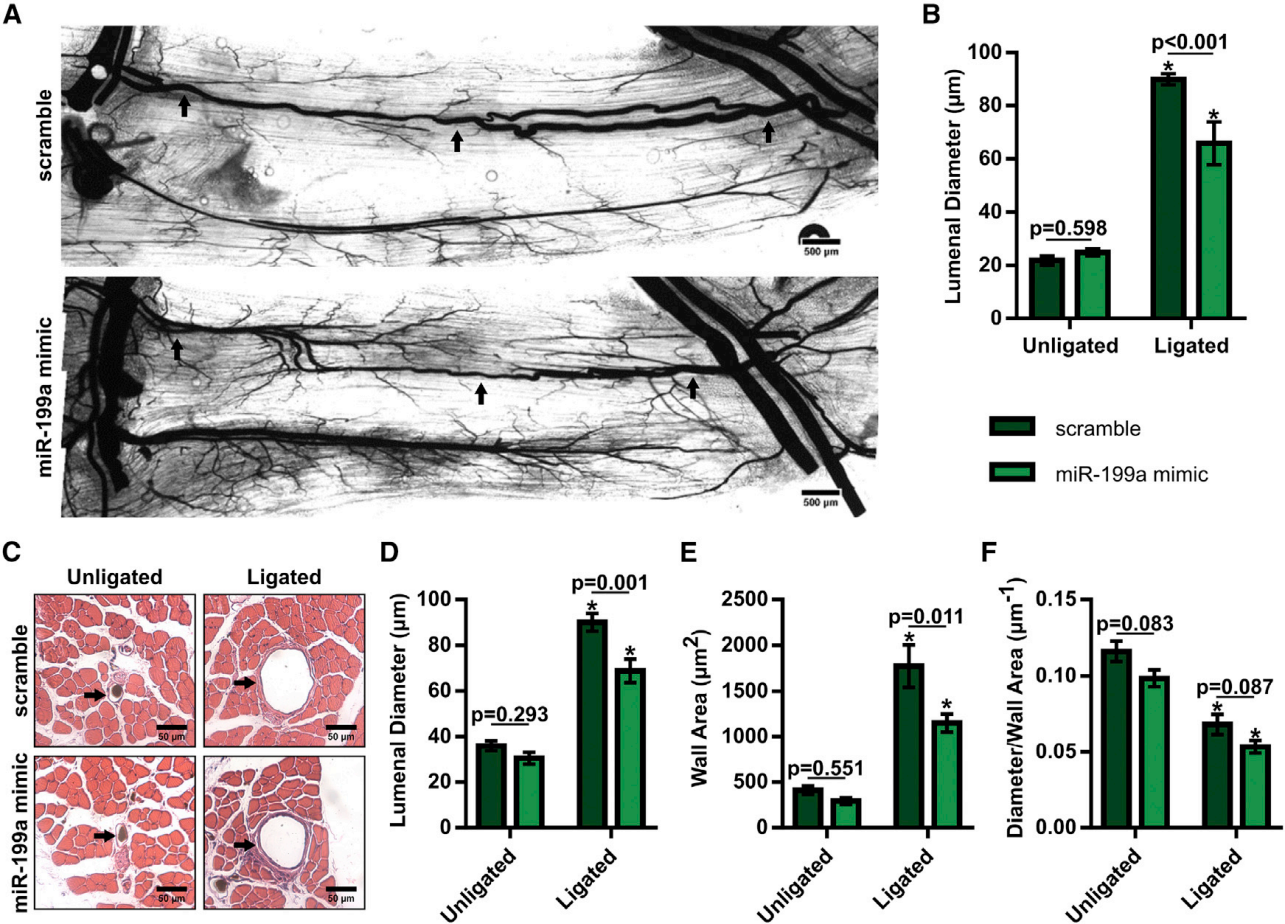
Overexpression of miR-199a Inhibits Arteriogenesis in BALB/c Mice (A) Representative whole-mount vascular cast images of gracilis collateral arteries 21 days post-FAL in scramble- (top) or miR-199a mimic- (bottom) treated BALB/c mice. (B) Bar graph of mean lumenal diameter along collateral artery length for ligated and unligated limbs of miR-199a mimic- and scramble-treated mice (n = 5–6 for mimic and scramble groups, respectively). *p < 0.001 versus unligated, two-way ANOVA followed by Holm-Sidak test for multiple comparisons. (C) Representative H&E-stained cross sections of collateral arteries from ligated and unligated limbs. (D–F) Bar graphs of lumenal diameter (D), wall area (E), and diameter per wall area (F) from H&E-stained cross sections (n = 5–6 for mimic and scramble groups, respectively). *p < 0.001 versus unligated, two-way ANOVA followed by Holm-Sidak test for multiple comparisons. Arrows indicate the primary collateral artery in (A) and (C). Data are mean ± SEM.

**Figure 5:**
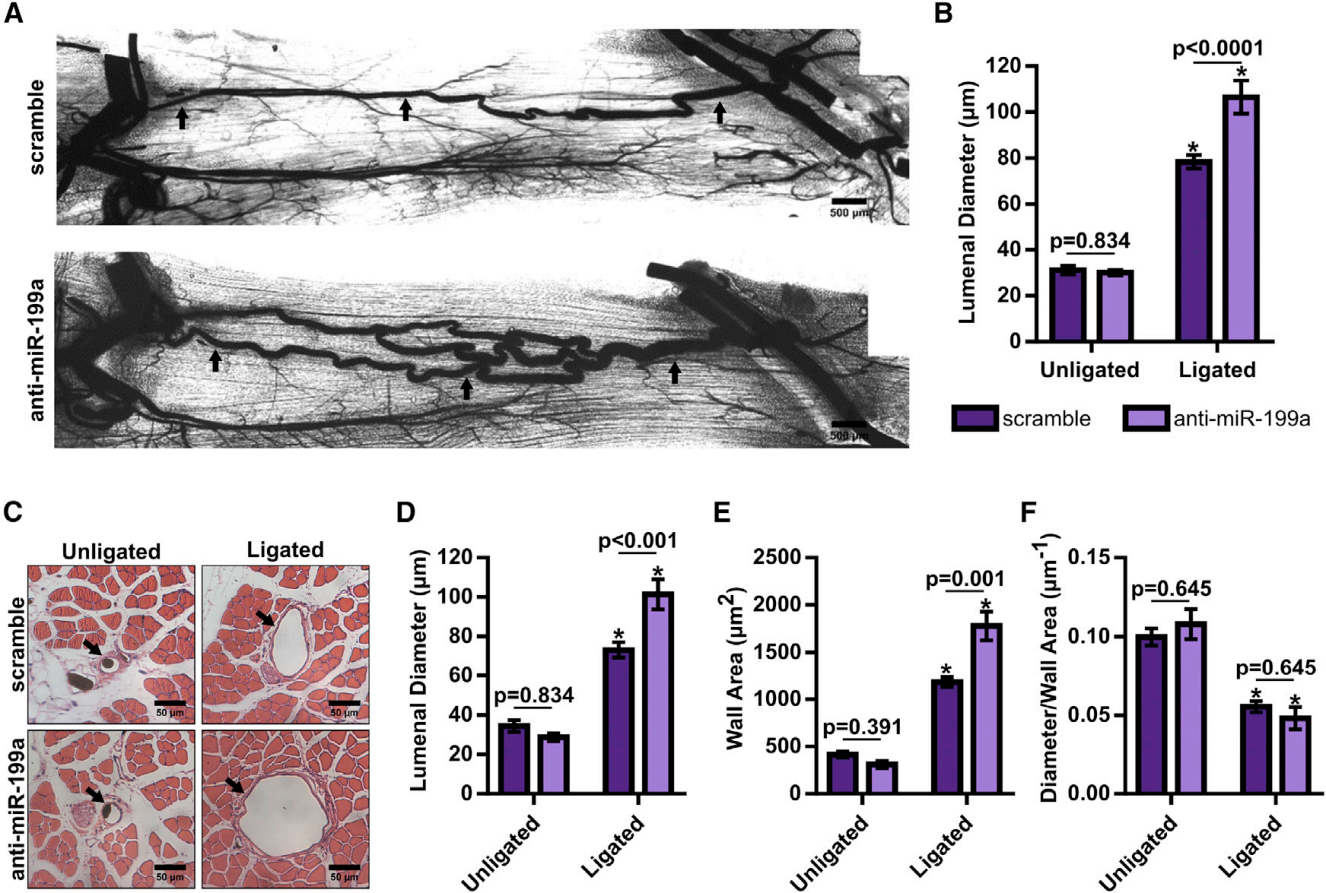
Inhibition of miR-199a Amplifies Arteriogenesis in BALB/c Mice following FAL (A) Representative whole-mount vascular cast images of gracilis collateral arteries 21 days post-FAL in non-targeting scramble- (top) or anti-miR-199a- (bottom) treated BALB/c mice. (B) Bar graph of mean lumenal diameter along collateral artery length for ligated and unligated limbs of anti-miR-199a- and scramble-treated mice (n = 6). *p < 0.001 versus unligated, two-way ANOVA followed by Holm-Sidak test for multiple comparisons. (C) Representative H&E-stained cross sections of collateral arteries from ligated and unligated limbs. (D–F) Bar graphs of lumenal diameter (D), wall area (E), and diameter per wall area (F) from H&E-stained cross sections (n = 6). *p < 0.001 versus unligated, two-way ANOVA followed by Holm-Sidak test for multiple comparisons. Arrows indicate the primary collateral artery in (A) and (C). Data are mean ± SEM.

We next examined whether overexpression of miR-199a affects arteriogenesis by measuring the lumenal diameter of gracilis collateral arteries 21 days post-FAL (Figures 4A and 5A). While both scramble- and miR-199a mimic-treated mice experienced significant (p < 0.0001) arteriogenesis in their ligated limbs compared to sham-operated controls, treatment with an miR-199a mimic significantly (p = 0.0008) reduced collateral artery growth by >25% (Figure 4B). Moreover, this reduction in arteriogenesis occurred at both the muscular (non-reversed) and saphenous (reversed) collateral artery regions (Figure S5). Cross-sectional analysis of these collateral arteries was used to confirm whole-mount diameter measurements and to determine collateral wall area (Figure 4C). Mice treated with miR-199a mimic experienced a lesser degree of enhancement in lumenal diameter (Figure 4D) and wall area (Figure 4E) than scramble controls, while there was no significant difference (p = 0.087) in the diameter-to-wall area ratio 21 days after FAL.

### Inhibition of miR-199a Amplifies Arteriogenesis in BALB/c Mice following FAL

In contrast, when we administered a single intramuscular injection of an anti-miR-199a locked nucleic acid oligonucleotide to mice immediately after FAL, arteriogenesis was significantly amplified (Figure 5A). Inhibition of miR-199a generated a 36% increase in collateral lumenal diameter 21 days post-FAL (p < 0.0001) (Figure 5B). Again, cross-sectional analysis was used to confirm whole-mount diameter measurements and to determine wall area (Figure 5C). Following FAL, intramuscular anti-miR-199a treatment produced increased collateral artery lumenal diameter (Figure 5D) and wall area (Figure 5E) compared to scramble-treated BALB/c mice, while there was no significant difference in the diameter-to-wall area ratio (Figure 5F). The enhancement in lumenal diameter and wall area in response to miR-199a inhibition was evident along the collateral at both the muscular (non-reversed) and saphenous (reversed) regions (Figure S6).

### Pericollateral Macrophage Recruitment Is Modulated by miR-199a

**Figure 6:**
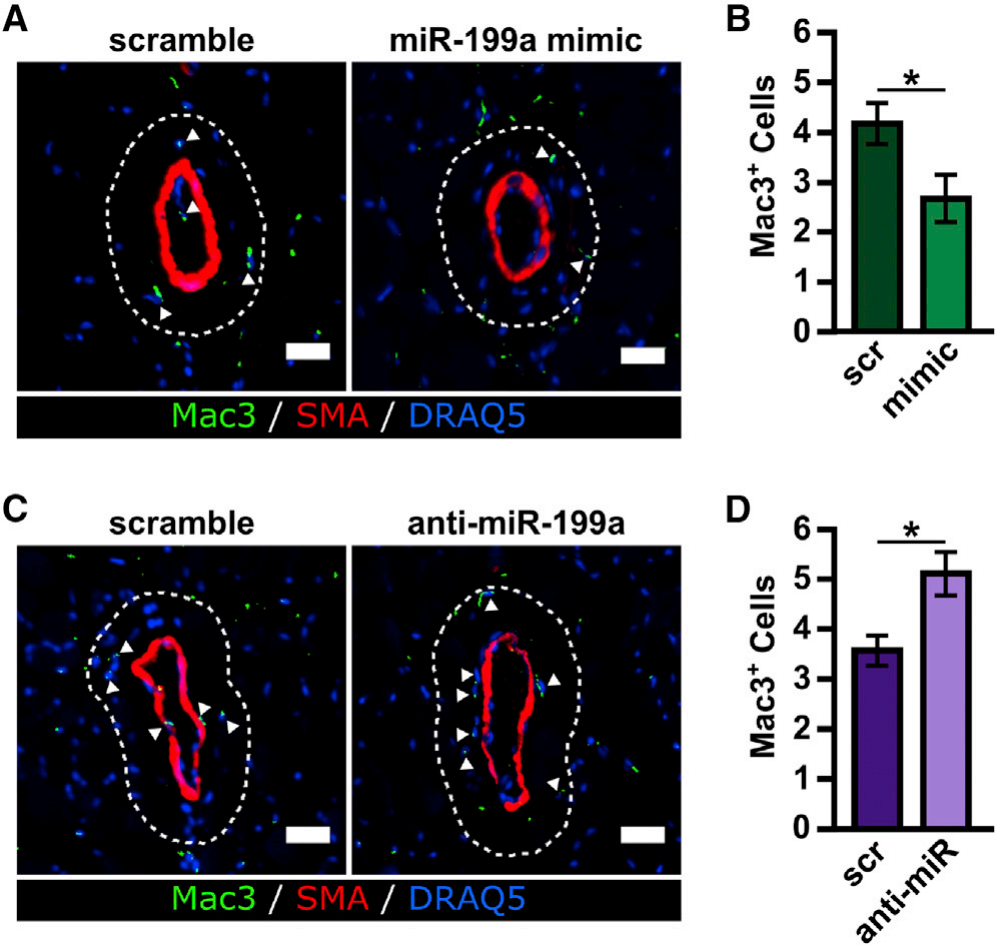
Pericollateral Macrophage Recruitment Is Modulated by miR-199a (A) Representative cross sections of gracilis collateral artery regions immunolabeled for macrophage marker, Mac3 (green), smooth muscle alpha actin (SMαA, red), and nuclei (DRAQ5, blue) in BALB/c mice treated with miR-199a mimic or scramble mimic 7 days post-FAL. Dotted line indicates the pericollateral region (25 μm from vessel wall) used for quantification. Arrowheads indicate Mac3^+^ cells (scale bar, 25 μm). (B) Bar graph of pericollateral Mac3^+^ cells (n = 4–5 for miR-199a mimic and scramble, respectively). *p < 0.05, Student’s t test. (C) Immunolabeled gracilis collateral artery regions, as in (A), in BALB/c mice treated with anti-miR-199a or scramble oligonucleotide 7 days post-FAL. (D) Bar graph of pericollateral Mac3^+^ cells (n = 3). *p < 0.05, Student’s t test. Data are mean ± SEM.

We next sought to determine whether modulating miR-199a expression altered macrophage recruitment, a necessary component of collateral artery growth,^32^, ^33^, ^34^, ^35^, ^36^, ^37^, ^38^ *in vivo*. miR-199a overexpression caused a 36% decrease in pericollateral Mac3^+^ macrophages 7 days post-FAL in BALB/c mice, whereas miR-199a inhibition caused a 43% increase in pericollateral macrophage recruitment (Figure 6). This trend was also observed when the muscular (non-reversed) and saphenous (reversed) collateral artery regions were assessed individually (Figure S7). We observed a similar trend across all groups when using F4/80^+^ as an additional macrophage-specific marker^39^ (Figure S8).

### miRNA-199a Inhibition Improves Gastrocnemius Muscle Composition in FAL-Operated BALB/c Mice

**Figure 7:**
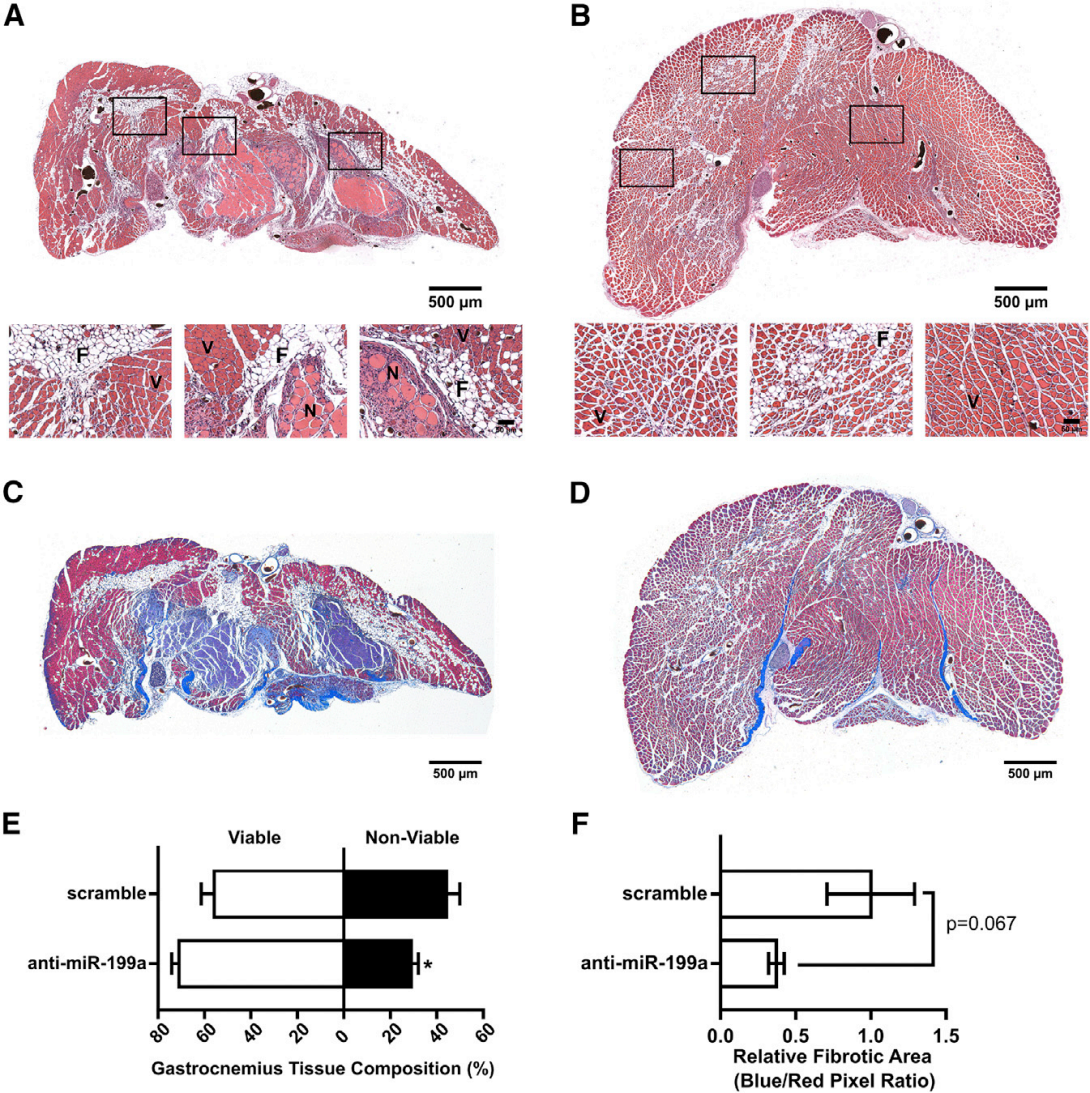
miRNA-199a Inhibition Improves Gastrocnemius Muscle Composition in FAL-Operated BALB/c Mice (A and B) Representative images of H&E staining of gastrocnemius muscle for ligated limb of BALB/c mice treated with scramble (A) or anti-miR-199a (B) locked nucleic acid oligonucleotide immediately after FAL (scale bar, 500 μm; inset scale bar, 50 μm). V, viable muscle; N, necrotic tissue; F, fibro-adipose tissue. (C and D) Representative images of Masson trichrome-stained gastrocnemius muscles from ligated limb of BALB/c mice treated with scramble (C) or anti-miR-199a (D) (scale bar, 500 μm). Blue staining is indicative of collagen and/or fibrotic content whereas red staining indicates healthy tissue. (E) Bar graph of the percentage of gastrocnemius muscle that is viable (white) or non-viable (black) at day 21 post-FAL in each group (n = 5). (F) Bar graph of the relative fibrotic area in gastrocnemius muscle at day 21 post-FAL in each group (n = 5). *p < 0.05 versus scramble, Student’s t test. Data are mean ± SEM.

Next, we examined the effect of altered miR-199a expression on the composition of ischemic muscle tissue downstream of the femoral artery occlusion. Gastrocnemius muscle tissue was categorized as viable (mature and regenerating fibers) or non-viable (necrotic and fibro-adipose tissue) by histological analysis 21 days after FAL (Figures 7A and 7B). Though muscle composition was not significantly altered in miR-199a mimic-treated mice (Figure S9), inhibition of miR-199a resulted in a greater than 34% reduction in the percentage of non-viable tissue in the gastrocnemius muscle (Figure 7E). Masson trichrome staining was used as an additional metric to assess fibrosis in the gastrocnemius muscle 21 days post-FAL.^40^, ^41^ Similar to H&E staining, anti-miR-199a-treated mice exhibited a trend toward decreased fibrotic area compared to scramble controls (Figures 7C, 7D, and 7F). There was no difference in fibrosis between miR-199a mimic- and scramble control-treated mice (Figure S9).

### Plasma miRNA-199a Expression Is Elevated in Patients with PAD

**Figure 8:**
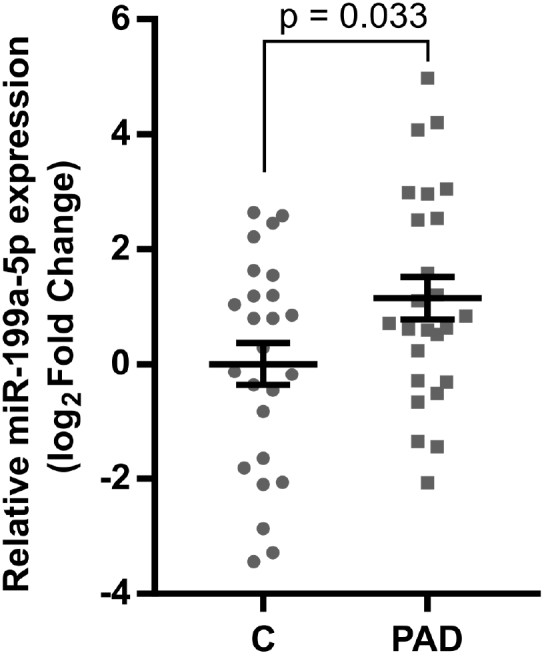
miR-199a-5p Expression Is Elevated in the Plasma of PAD Patients with Intermittent Claudication Log_2_ fold change of miR-199a-5p expression in plasma from human PAD patients with intermittent claudication (PAD) versus a risk factor control population (C). Plasma miR-199a-5p expression was normalized to RNU6 (n = 25). *p < 0.05, Student’s t test. Data are mean ± SEM.

Finally, we sought to determine if miR-199a expression is altered in human PAD patients. A diagnosis of PAD with intermittent claudication was based on having one of the following: (1) an ankle-brachial index (ABI) < 0.9, (2) a previous peripheral vascular intervention, or (3) an abnormal tibial-brachial index (TBI), without any corresponding foot ulcers or resting pain. Plasma was collected from PAD patients with intermittent claudication (minimum ABI = 0.75 ± 0.26) and risk factor-controlled patients (minimum ABI = 1.09 ± 0.12) who otherwise exhibited similar PAD risk factors, such as age, sex, diabetes, hypertension, and hyperlipidemia (Table S2). We found relative plasma miR-199a expression to be significantly elevated (p = 0.033) in PAD patients relative to risk factor control patients (Figure 8).

## Discussion

The primary goal of this study was to determine whether mechanosensitive miRNAs regulate endogenous collateral artery growth and perfusion recovery following femoral arterial occlusion. First, we identified candidate regulators of arteriogenesis by comparing differential miRNA expression in ECs exposed to shear stress waveforms corresponding to moderate and amplified arteriogenesis responses *in vivo*. Among these candidate miRNAs, miR-199a was significantly downregulated by the amplified arteriogenesis (reversed flow) waveform and shown to regulate pro-arteriogenic molecule (IKKβ and Cav1) expression and monocyte adhesion to shear stress-exposed endothelium. *In vivo*, the overexpression of miR-199a limited foot perfusion, arteriogenesis, and monocyte recruitment in the mouse FAL model. In contrast, the inhibition of miR-199a elicited complete foot perfusion recovery, substantially enhanced collateral arteriogenesis via increased pericollateral macrophage recruitment, and considerably improved gastrocnemius muscle tissue composition. Finally, we showed miR-199a expression is elevated in the plasma of PAD patients with intermittent claudication compared to a risk factor control population, further indicating its relevance in human disease. We have, therefore, identified miR-199a as a novel mechanosensitive miRNA that regulates perfusion recovery and arteriogenesis. miRNA-199a inhibition may represent a new target for the therapeutic stimulation of arteriogenesis and the treatment of PAD.

### Identification of miRNA-199a as a Novel Mechanosensitive Regulator of Pro-arteriogenic Gene Expression

Our strategy of interrogating differential mechano-signaling in ECs exposed to a biomimetic amplified arteriogenesis waveform represents a unique, transformative approach for identifying miRNA regulators of vascular growth and perfusion recovery in response to arterial occlusion(s). This strategy was enabled by our group’s previous development of a transillumination laser speckle flowmetry method customized for measuring blood velocities in the collateral arteries of the mouse gracilis adductor muscle.^11^ Using this method, we were able to determine segment-to-segment hemodynamic changes induced by FAL,^11^ correlate these to variations in arteriogenesis,^10^ and directly apply these flow waveforms to ECs *in vitro*. The utility of this approach is supported by our present results, as well as by our approach’s identification of miR-100 as another candidate miRNA (Figure 1C). Inhibition of miR-100 has been previously shown to enhance perfusion recovery following FAL.^20^ When considered in light of our findings, we postulate that the enhancement of shear stress-mediated collateral arteriogenesis may be a probable component of perfusion recovery with anti-miR-100 treatment.

We also identified miR-34a and miR-146a as potential mechanosensitive regulators of arteriogenesis that could be targeted for therapeutic arteriogenesis (Figure 1). Indeed, we have recently shown that miR-146a-5p inhibition improves collateral artery growth following FAL.^42^ However, based on their known downstream targets and functions, we postulate that these miRNAs could also be subject to the so-called Janus phenomenon, wherein pro-arteriogenic therapies also tend to promote atherosclerosis.^43^ To this end, both miR-34a and miR-146a have been found to be significantly upregulated in human atherosclerotic plaques.^44^ Moreover, deletion of miR-146a in the vasculature increases EC activation and atherogenesis, though miR-146a deletion in bone marrow-derived cells reduces atherogenesis despite paradoxically elevating circulating pro-inflammatory cytokines in Ldlr^−/−^ mice on a high-cholesterol diet.^45^

To date, miR-199a has been mostly studied for its roles in cardiac hypertrophy,^46^, ^47^ smooth muscle proliferation,^48^ angiogenesis,^49^, ^50^, ^51^, ^52^, ^53^, ^54^ and as a tumor suppressor in a variety of cancers.^29^, ^50^, ^52^, ^55^, ^56^, ^57^, ^58^, ^59^ However, miR-199a also regulates several genes important for arteriogenesis via direct seed sequence binding to target mRNAs. To this end, it has been shown that miR-199a negatively regulates nuclear factor κB (NF-κB), which is necessary for arteriogenesis,^60^ by directly targeting IKKβ.^29^ Additionally, Cav1, a critical component of caveolae shown to be key in mechanotransduction,^61^ the response to arterial occlusion,^30^, ^31^ and neovascularization,^62^ has been previously shown to be a direct target of miR-199a.^63^, ^64^, ^65^, ^66^, ^67^ Here we found that miR-199a alters both IKKβ and Cav1 expression in ECs exposed to our pro-arteriogenic biomimetic shear stress waveforms. Moreover, Cav1 protein expression was regulated by miR-199a expression in the gracilis muscle *in vivo*, suggesting a potential pathway by which altered miR-199a is able to regulate arteriogenesis. Furthermore, through both loss- and gain-of-function studies, we found miR-199a to regulate monocyte adhesion to ECs, a key aspect of arteriogenesis. Altogether, this made miR-199a a compelling candidate miRNA to study in the context of arteriogenesis *in vivo*.

### Previous Studies Implicating a Role for miRNAs in Regulating Arteriogenesis

Despite a number of previous reports indicating miRNA regulation of perfusion recovery in hindlimb ischemia models,^19^, ^20^, ^21^, ^22^, ^23^, ^24^, ^25^, ^26^, ^27^, ^68^ only a few of these miRNAs (e.g., miR-155, miR-17∼92a, miR-93, miR-487b, and miR-352) have been suggested to have a role in arteriogenesis per se. Inhibition of miR-155 attenuates blood flow recovery and leukocyte recruitment, despite promoting angiogenesis in the ischemic tissue of FAL-treated mice, thereby indirectly implying a role for miR-155 in arteriogenesis. However, collateral diameters were not explicitly reported.^26^ In another study, endothelial-specific deletion of the miR-17∼92 cluster increased foot perfusion 14 days following FAL as well as arteriole density in FAL-operated limbs.^27^ More recently, overexpression of miR-93 was sufficient to rescue the impaired perfusion recovery, capillary density, and arteriolar density observed in miR-106b-93-25^−/−^ mice following femoral artery excision via an miR-93-IRF9-IRG1-itaconic acid pathway leading to increased M2-like macrophage polarization in ischemic tissue.^68^ Additionally, miR-329, miR-487b, miR-494, and miR-495 inhibition all led to a 25%–40% increase in blood flow recovery following FAL, though only miR-487b inhibition increased vessel diameters from PBS-treated controls.^24^ While these studies further corroborate miRNA-mediated regulation of arteriogenesis, it is difficult to directly compare our results due to differences in oligonucleotide dosing, the extent of ischemia in the various animal models, and methods for assessing arteriogenesis.

To our knowledge, only one other previous study has examined flow-responsive miRNAs in the regulation of arteriogenesis. In a rat arteriovenous (AV) shunt model of amplified arteriogenesis due to chronically elevated blood flow, 94 miRNAs were found to be differentially expressed.^69^ Of these, miR-353 was found to be downregulated in the pro-growth, chronically elevated blood flow condition. Subsequent miR-352 inhibition increased the number of collateral arteries, proliferation within the vessel wall, and collateral flow,^69^ though the direct regulation of endothelial miR-352 expression by shear stress was not shown. Interestingly, miR-199a-5p was also downregulated (−2.56-fold in AV shunt [i.e., pro-growth] versus control) in this alternative model of amplified arteriogenesis, providing further support that the inhibition of miR-199a promotes arteriogenesis.

### miRNA-199a as a Regulator of Arteriogenesis Leading to Enhanced Perfusion Recovery

In testing our hypothesis that miR-199a negatively regulates perfusion recovery and arteriogenesis *in vivo*, we chose to perform our FAL model in BALB/c mice to enable the necessary dynamic range for both loss- and gain-of-function studies, as these mice exhibit a more blunted arteriogenic response compared to C57BL/6 mice.^70^, ^71^ Following FAL, scramble-treated mice exhibited incomplete perfusion recovery as expected, though they did recover 80%–90% perfusion in ligated limbs compared to the ∼50%–60% perfusion recovery seen in previous studies.^71^ This greater baseline recovery response was likely due to the more mild nature of our ligation scheme; nonetheless, we were still able to observe significant changes in foot perfusion with the modulation of miR-199a expression, similar to those seen by previous miRNA studies.^20^, ^23^, ^72^ We also emphasize that our FAL model is still severe enough to elicit a substantial transformation of skeletal muscle into necrotic and fibro-adipose tissue in the calf, which is characteristic of PAD in humans.

Corresponding to the observed changes in foot perfusion, we found that miR-199a negatively regulates arteriogenesis. Our results indicating miR-199a modulates monocyte and/or macrophage recruitment are consistent with the known necessary role for leukocyte recruitment in arteriogenesis,^32^, ^35^, ^36^, ^73^ and they suggest that this is the mechanism through which miR-199a regulates arteriogenesis following FAL. Critical in determining the reperfusion response, alterations to endogenous collateral growth can lead to substantial changes in collateral conductance. To this end, miR-199a overexpression attenuated collateral artery growth ∼25% (∼2.4-fold decrease in conductance) and miR-199a inhibition enhanced arteriogenesis by 36% (>3.4-fold conductance increase). Of particular importance for potential clinical translation, collateral arteries in anti-miR-199a-treated mice were morphologically normal, as there was no difference in the lumenal diameter-to-wall area ratio when miR-199a expression was altered (Figure 6F).

In addition to arteriogenesis, perfusion recovery following FAL can also be dependent upon angiogenesis^7^ and/or tissue clearance and skeletal muscle regeneration.^8^ Here we did not observe any significant differences in gastrocnemius capillary density within viable and non-viable tissue regions in anti-miR-199a-treated mice at day 21 post-FAL (Figure S10). However, we did find that a single bolus of anti-miR-199a oligonucleotide was sufficient to improve gastrocnemius muscle tissue composition in FAL-operated mice (Figure 8), though there was no significant change in muscle composition with miR-199a overexpression (Figure S9). While it is probable that this improvement in muscle composition is a consequence of enhanced downstream tissue perfusion due to amplified arteriogenesis with miR-199a inhibition, miR-199a could also play a direct role in tissue clearance and regeneration. Indeed, miR-199a has been previously implicated in tissue fibrosis,^66^ myoblast proliferation and differentiation,^74^ and adipocyte differentiation,^75^ all of which are consistent with the hypothesis that miR-199a can directly regulate skeletal muscle proliferation and/or differentiation in a hypoxic environment. However, in our hands, miR-199a inhibition did not alter cell survival of C2C12 myoblasts after exposure to hypoxia and serum starvation conditions (Figure S11). Though future work could test the cell-specific contribution of miR-199a in response to FAL, our present results indicate that improved muscle composition with anti-miR-199a treatment is secondary to enhanced upstream arteriogenesis.

### Clinical Perspective

Based on both our results and previous studies, we postulate that miRNA-199a inhibition represents a potentially promising treatment for arterial occlusive diseases. Most patients with PAD also have concomitant coronary artery disease (CAD).^76^ Previously, miR-199a-5p was shown to be upregulated in patients with CAD versus matched controls,^77^ though its expression in PAD was unknown. Here we observed that plasma miR-199-5p expression is elevated in PAD patients with intermittent claudication versus a risk factor control population. Moreover, our *in vivo* results indicate that miR-199a inhibition improves limb perfusion by enhancing arteriogenesis and reduces the fibro-adipose composition of ischemic muscle, which is often found in PAD patients.^78^, ^79^, ^80^, ^81^ Together, these data indicate anti-miR-199a-5p delivery strategies may have a therapeutic benefit for PAD patients.

## Materials and Methods

### HUVEC Culture

HUVECs purchased from VEC Technologies (Rensselaer, NY) were thawed and maintained on 0.1% gelatin-coated flasks in M-199 medium (Lonza, Basel, Switzerland), supplemented with 10% fetal bovine serum (Life Technologies, Grand Island, NY), 100 U/mL penicillin-G + 100 μg/mL streptomycin (Life Technologies), 2 mmol/L L-glutamine (Life Technologies), 5 μg/mL EC growth supplement (Biomedical Technologies, Stoughton, MA), and 10 μg/mL heparin (Sigma-Aldrich, St. Louis, MO). For each set of experimental comparisons, cells were used from the same cell line between subculture passages 2 and 3.

### *In Vitro* Exposure of ECs to Biomimetic Shear Stress Waveforms

HUVECs were plated on cell culture grade plastic dishes coated with 0.1% gelatin and grown to confluence. A cone and plate flow apparatus,^82^ which maintains cells at 5% CO_2_ and 37°C, was used to induce a shear stress protocol. The applied shear stress protocol consisted of a 24-hr preconditioning period at a steady 15 dyne/cm^2^, which was then either increased to 30 dynes/cm^2^ (non-reversed flow) or increased to 30 dynes/cm^2^ and reversed in direction (reversed flow) to simulate relative hemodynamics previously quantified in our *in vivo* FAL model.^11^ Fresh culture medium, consisting of M199 with 4% dextran from *Leuconostoc* spp (Sigma-Aldrich, M_r_ ∼500,000), 2% fetal bovine serum, 100 U/mL penicillin-G + 100 μg/mL streptomycin, 2 mmol/L L-glutamine, 5 μg/mL EC growth supplement, and 10 μg/mL heparin, was added to cells before exposure to shear stress, and it was continuously exchanged throughout the duration in the cone and plate apparatus.

### HUVEC Microarray Gene Expression Profiling

Microarray analysis was performed as previously described and is publicly available at GEO: GSE46248 (https://www.ncbi.nlm.nih.gov/geo/). Volcano plots were generated using the expression change and false discovery rates (FDRs) from the reversed versus non-reversed (RvN) and reversed versus control (RvC) datasets, respectively.

### Transfection of miRNA Antagomirs and Mimics of HUVECs

At 24 hr prior to the exposure of HUVECs to flow conditions, HUVECs were plated without antibiotics on 0.1% gelatin-coated plates in serum-free M199 (Life Technologies) supplemented with 10% fetal bovine serum, 2 mmol/L L-glutamine, 5 μg/mL EC growth supplement (Biomedical Technologies, Stoughton, MA), and 10 μg/mL heparin (Sigma-Aldrich). After cells were allowed to adhere for 2 hr after plating, cells were transfected using Lipofectamine RNAiMax (Invitrogen, Carlsbad, CA), according to the manufacturer’s instructions. For inhibitor experiments, HUVECs were transfected with 20 nM scramble or miR-199a-5p locked nucleic acid (LNA) inhibitors (Power Inhibitors, Exiqon, Vedbaek, Denmark). For overexpression experiments, HUVECs were transfected with 20 nM scramble or miR-199a-5p LNA mimic (miRCURY LNA mimics, Exiqon).

### HUVEC RNA Isolation and qRT-PCR

Total RNA was extracted using the PureLink total RNA purification system (Life Technologies) using the on-column DNase protocol (Life Technologies), according to the manufacturer’s instructions. RNA concentration and purity were determined with a NanoDrop spectrophotometer in duplicate.

For qRT-PCR, 500 ng total RNA was reverse transcribed using the miScript II reverse transcription kit (QIAGEN, Hilden, Germany, 218160) with HiFlex buffer mix, according to the manufacturer’s instructions. Following reverse transcription, RT-PCR was performed on 20 ng cDNA using miScript SYBR Green PCR kit (QIAGEN, 218073) and miScript Primer Assays (QIAGEN) for U6 (MS00033740), miR-199a-5p (MS00006741), miR-146a-5p (MS00003535), and miR-29a (MS00003262) on a CFX96 Real Time Detection System (Bio-Rad). Normalized expression to U6 was quantified using the comparative 2^ΔΔCt^ method.

Target mRNA expression was assessed by qRT-PCR on 12.5 ng reverse transcribed cDNA with FastStart SYBR Green (Roche Applied Sciences, Penzberg, Germany) on a CFX96 Real Time Detection System (Bio-Rad, Hercules, CA). Primers used were as follows: CD44 (forward 5′-GCAGCCAACTTCCGAGGCAGC-3′, reverse 5′-CGGAGGACGGGACGAGGATGG-3′), CCND1 (forward 5′-AACTACCTGGACCGCTTC-3′, reverse 5′-GAGTTGTCGGTGTAGATGC-3′), and IKKβ (forward 5′-GCCTGGGAAATGAAAGAGCG-3′, reverse 5′-ATCTGCTCACCTGTTTCCTGA-3′). Expression was normalized to β2-microglobulin (forward 5′-AGCATTCGGGCCGAGATGTCT-3′, reverse 5′-CTGCTGGATGACGTGAGTAAACCT-3′), which is endogenously expressed and is not altered by many stimuli, including shear stress.^83^

Cav1 mRNA expression was assessed by qRT-PCR on 12.5 ng cDNA with SensiMix II (Bioline, London, UK, BIO-83005) using Cav1-FAM (Hs00971716_m1) and B2M-VIC (Hs00187842_m1) TaqMan primer probes, purchased from Thermo Fisher Scientific, on a CFX96 Real Time Detection System. Normalized expression was quantified using the comparative 2^ΔΔCt^ method.

### Monocyte Adhesion Functional Assay

Human-derived monocytes (THP-1 cell line) were purchased from the ATCC. Monocytes were thawed and maintained in RPMI 1640 (11875-093, Life Technologies) + 10% fetal bovine serum (Life Technologies) + 0.05 mM β-mercaptoethanol per ATCC culture instructions. Monocytes sub-cultured once cell density approached 800,000 cells/mL. Cells were used between passages 2 and 6.

Prior to the adhesion assay, cells were counted to obtain 3,000,000 cells/plate of HUVECs. Cells were pelleted, washed with PBS, pelleted, and then re-suspended in serum-free RPMI medium at 1,000,000 cells/mL. Thawed calcein acetoxymethyl (AM) was added at 1 μg/mL and incubated with cells for 15 min at 37°C. After 15 min, the reaction was stopped by adding excess serum-free RPMI to the cell solution then pelleted. Cells were washed once with serum-free M199 medium, pelleted, and then re-suspended in serum-free M199 at 500,000 cells/mL. Immediately following completion of flow exposure to HUVECs, flow medium was removed by aspiration. HUVECs were quickly washed with serum-free M199 medium. This medium was then aspirated off and 6 mL serum-free M199 + monocytes (3,000,000/plate) were added to and incubated with HUVECs for 30 min at 37°C. Following the 30 min, cells were washed twice with PBS to remove unbound monocytes. Adhered monocytes and HUVECs were fixed with 4% paraformaldehyde (PFA) for 10 min followed by two washes with PBS. Coverslips were mounted with Prolong Gold (Life Technologies). Plates were then imaged using a Nikon TE2000 C1 laser-scanning confocal microscope. Nine randomly selected fields of view (FOVs) per condition for 4 independent experiments were obtained. Images were de-identified and randomized in MATLAB. Images were converted to 8-bit images, set to an equivalent threshold, and bound monocytes were quantified using Fiji’s Analyze Particles tool (20 μm^2^ minimum particle size). Results were centered on the mean of all conditions within each independent experiment.

### Mice

All animal protocols were approved by the Institutional Animal Care and Use Committee at the University of Virginia (protocol 3814), and they conformed to all regulations for animal use outlined in the American Heart Association Guidelines for the Use of Animals in Research. BALB/c mice were purchased from Charles River Laboratory (Wilmington, MA). All animals were housed in the animal facilities at the University of Virginia.

### FAL Model

We used a previously detailed FAL scheme^10^, ^84^, ^85^ that produces consistent arteriogenesis in the collateral arteries of the gracilis adductor muscles,^7^, ^8^, ^10^, ^86^, ^87^, ^88^, ^89^ along with minimal heterogeneity in the baseline collateral structure and with known changes in flow direction from baseline. Male mice, 10–12 weeks of age, were anesthetized (intraperitoneally [i.p.], 120 mg/kg ketamine, 12 mg/kg xylazine, and 0.08 mg/kg atropine), depilated, and prepped for aseptic surgery. On the left leg, an incision was made directly above and along the femoral artery, which was gently dissected from the femoral vein and nerve between the bifurcation of the superior epigastric artery and popliteal artery. Two 6.0 silk sutures were placed immediately distal to the epigastric artery, which served as the origin of the muscular branch artery in all mice, and the artery segment between the two ligatures was then severed with micro-dissecting scissors. The surgical site was then closed with 5.0 prolene sutures. A sham surgery, wherein the femoral artery was exposed, but not ligated, was performed on the right hindlimb (i.e., on the other leg). Animals received one injection of buprenorphine for analgesia at the time of surgery and a second dose 8–12 hr later.

### *In Vivo* miR-199a Antagomir Treatment

Anti-miR-199a-5p (5′-TAGTCTGAACACTGGG-3′) and scramble control (5′-ACGTCTATACGCCCA-3′) LNA oligonucleotides were purchased from Exiqon. Oligonucleotides were reconstituted in sterile TE buffer and stored at 1.2 nmol/μL at −20°C. Prior to use, aliquots of oligonucleotides were thawed and diluted in sterile saline to a final concentration of 0.25 nmol/μL. Immediately following FAL, 7.5 nmol oligonucleotide was injected into each (ligated and sham-operated) gracilis muscles.

### *In Vivo* miR-199a Mimic Treatment

*In vivo* ready miRVana miR-199a-5p (4464070, MC10893) mimic and scramble control (4464061) mimic were purchased from Ambion. Mimics (250 nmol) were reconstituted in sterile nuclease-free water and stored at 0.5 nmol/μL at −20°C. Prior to use, aliquots of oligonucleotides were thawed and diluted in sterile saline to a final concentration of 0.25 nmol/μL. Immediately following FAL, 7.5 nmol oligonucleotide was injected into both ligated and sham-operated gracilis muscles.

### Laser Doppler Perfusion Imaging

For monitoring blood flow recovery and post-surgical ischemia, mice were anesthetized via 1.5% isoflurane under constant oxygen. Mice were placed in a prone position and the soles of the feet were scanned (PeriCam PSI, PeriMed, Stockholm, Sweden). Mean foot perfusion was used to calculate relative perfusion ratio (ratio of ligated over unligated).

### Tissue Harvesting for miRNA Expression

At 7 days after FAL, mice were anesthetized (i.p., 120 mg/kg ketamine, 12 mg/kg xylazine, and 0.08 mg/kg atropine) and euthanized via an overdose of pentobarbital sodium and phenytoin sodium (Euthasol, Virbac, Fort Worth, TX). The left ventricle was cannulated with a 23G catheter (right ventricle was carefully opened to act as a sink for perfusate), and the entire body was perfused with 7 mL Tris-CaCl_2_ (0.1 g/L CaCl_2_) containing 2% heparin, 2 mmol/L adenosine (16404, Fisher Scientific, Pittsburgh, PA), and 0.1 mmol/L papaverine (P3510, Sigma-Aldrich, St. Louis, MO) to clear and vasodilate the downstream vasculature at a constant rate of 1.5 mL/min (PHD2000, Harvard Apparatus). Once perfused, we waited 5 min to enable vasodilation. The entire body was then perfused with 14 mL Tris-CaCl_2_, and both gracilis muscles were dissected free, placed in RNAlater (Ambion), and stored at −20°C.

### RNA Isolation from Tissues and qRT-PCR

Excess RNAlater was removed from tissues. Tissues were then incubated in 450 μL TRIzol reagent for 5 min at room temperature. Tissues were then placed on ice and homogenized using a power homogenizer (Omni International, Kennesaw, GA) in short bursts to avoid overheating. Following homogenization, an additional 550 μL TRIzol reagent was added. Samples were incubated for another 5 min at room temperature to ensure complete lysis. 200 μL chloroform was added to each sample. Samples were then shaken vigorously for 15 s and incubated for 3 min at room temperature. Following this incubation, samples were centrifuged for 10 min at 12,000 × *g* at 4°C. The resulting aqueous layer was carefully removed, placed in a new RNA and DNase-free tube, and an equal volume of 70% ethanol was added to save the aqueous layer. RNA isolation then proceeded using the PureLink total RNA purification system (Life Technologies) with the on-column DNase protocol (Life Technologies), according to the manufacturer’s instructions. RNA concentration and purity were determined with a NanoDrop spectrophotometer in duplicate.

For reverse transcription (Applied Biosystems, 4366596) and miR-199a-5p detection, TaqMan miRNA assays (Applied Biosystems, 4427975) were used. Real-time qPCRs were done on an ABI Prism 7900 HT detection system (Applied Biosystems). Gene expression was normalized to RNU6 (Applied Biosystems, 4427975), and the relative expression was determined with the comparative 2^ΔΔCt^ method.

### Protein Expression of miR-199a-5p Targets

HUVECs were directly lysed in ice-cold radioimmunoprecipitation assay (RIPA) buffer (Sigma, R0278) with protease inhibitor (Sigma, 1:100, P8340). For mouse tissues, half of the entire gracilis muscle was placed in 1 mL RIPA buffer supplemented with protease inhibitor (Sigma, 1:100, P8340) on ice, and then it was homogenized using a power homogenizer (Omni International) in short bursts to avoid overheating. Lysed samples (both HUVEC and gracilis muscle) were then cleared for 30 min at 4°C under constant agitation. Samples were centrifuged for 1 min at 10,000 × *g*, the supernatant was collected, and a Pierce bicinchoninic acid (BCA) assay (Thermo Fisher Scientific, 23225) was used to determine total protein concentration. Samples were diluted 1:1 in 4× XT sample buffer (Bio-Rad, 161-0791) with XT reducing reagent (Bio-Rad, 161-0792, 1× final concentration) and boiled for 10 min. Equal protein was loaded onto a 4%–12% Bis-Tris Criterion XT pre-cast gel (Bio-Rad, 3450123) and run at constant voltage in XT 3-(N-morpholino)propanesulfonic acid (MOPS) running buffer (Bio-Rad, 161-0791). Protein was then transferred for 1 hr at 1 A constant current onto a nitrocellulose membrane. After transfer, membranes were stained with Ponceau S for 15 min at room temperature to determine total protein. Blots were then de-stained in PBS and blocked for 1 hr at room temperature in Tris-buffered saline with Tween (TBST) + 5% BSA and then incubated with primary antibodies overnight at 4°C. Western blots were performed by using primary antibodies directed against IKKβ (Abcam, 1:1,000, ab124957) and Cav1 (Abcam, 1:1,000, ab2910). Following overnight incubation, blots were washed 5 times for 5 min in TBST before being incubated in secondary antibody for 1 hr at room temperature. Goat anti-rabbit horseradish peroxidase (HRP)-conjugated secondary antibodies were purchased from Cell Signaling Technology (Danvers, MA, 7074) and used at a 1:5,000 dilution. Blots were again washed 5 times in TBST. Bands were then developed using Clarity Western ECL Substrate (Bio-Rad, 170-5061), followed by detection using a ChemiDoc Touch Imaging System (Bio-Rad). Images were quantified using Image Studio Lite software program (LI-COR Biosciences, Lincoln, NB). Total IKKβ and Cav1 expression was normalized to total protein, as determined by Ponceau staining. Each set of HUVEC biological replicates was mean centered to account for variation between gels.

### Tissue Harvesting for Whole-Mount Vascular Casting and Cross-Sectional Analysis

For the analysis of lumen diameters in the gracilis collateral arteries and to enable sectioning at specific regions, vascular casting was performed using an opaque polymer that allows for accurate lumenal diameter measurements.^88^ At 21 days after FAL, mice were anesthetized (i.p., 120 mg/kg ketamine, 12 mg/kg xylazine, and 0.08 mg/kg atropine), euthanized via an overdose of pentobarbital sodium and phenytoin sodium (Euthasol), and then the abdominal aorta was cannulated. The lower body was then perfused with 7 mL 2% heparinized saline with 2 mmol/L adenosine (16404, Thermo Fisher Scientific) and 0.1 mmol/L papaverine (P3510, Sigma-Aldrich) to clear and vasodilate the downstream vasculature at a constant rate of 1 mL/min (PHD2000, Harvard Apparatus). Once perfused, we waited 5 min to enable vasodilation. Tissues were then perfused with 3 mL 4% PFA solution (19943, Affymetrix, Cleveland, OH) at 1 mL/min and allowed to fix for 10 min. The lower body was then perfused with 0.8 mL MICROFIL casting agent (Flow Tech, Carver, MA) at a constant speed of 0.15 mL/min. The viscosity of MICROFIL was adjusted to minimize transport across capillaries. After curing for 1.5 hr at room temperature, gracilis muscles were dissected free and then cleared in 50% glycerol in PBS overnight. Cleared tissues were mounted between two coverslips using 500-μm-thick spacers (645501, Grace Bio-Labs) to keep constant thickness between muscles. Muscles were imaged using transmitted light at 4× magnification on a Nikon TE200 inverted microscope with a charge coupled device (CCD) camera (Quantifier, Optronics). Individual FOV were montaged together (Photoshop CS2, Adobe Systems).

For analysis of lumen diameters from intact gracilis collateral whole mounts (i.e., vascular casting), collateral entrance regions were defined according to the following method. A cropped portion (560 × 560 μm) of the montaged image (previously randomized and de-identified) was taken of the collateral artery at the first visible branchpoint of a terminal arteriole from the primary collateral as it extended from either the muscular branch or saphenous artery, as previously described.^10^ After each cropped image region was taken, all images were randomized and de-identified. The mean diameter was then taken from 4–5 separate diameter measurements along the length of cropped portion of the collateral artery.

After imaging, muscles were rehydrated, cut, and then paraffin embedded for cross-sectional analysis at the muscular branch and saphenous artery entrance regions to the collateral arteries. Resulting cross sections were rehydrated and immunolabeled for the macrophage marker Mac3 or F4/80 (day 7 post-FAL) and H&E stained for collateral artery structure analysis (day 21 post-FAL).

### Cross-Sectional Analysis of Collateral Artery Structure

Sections (5-μm thickness) of paraffin-embedded muscle from the muscular and saphenous regions were labeled for H&E. Individual FOV encompassing the collateral vessels were imaged with a 40× water objective on a Zeiss inverted microscope (Zeiss Axioskop, Thornwood, NY) with a CCD camera (Quantifier, Optronics). All images were randomized and de-identified prior to analysis. Lumenal diameter, wall area, and wall thickness were determined using Fiji.^90^

### Immunofluorescence Labeling of Pericollateral Macrophages

Sections (5-μm thickness) of paraffin-embedded muscle from the muscular and saphenous regions were rehydrated and subjected to heat-mediated antigen retrieval for 10 min in a citrate-based antigen retrieval buffer (Vector Laboratories, Burlingame, CA; H-3300). Slides were then quenched of endogenous peroxidase activity with a 30-min incubation in 3% H_2_O_2_, blocked, and labeled with rat-anti-Mac3 (1:100, M3/4 clone, 550292; BD Biosciences, San Jose, CA) overnight at 4°C. Slides were washed and incubated with a biotinylated sheep-anti-rat secondary antibody (Abcam, Cambridge, MA; ab6851, 1:500) for 1 hr at room temperature. Slides were washed and incubated with an avidin-biotin complex (Vectastain ABC solution, Vector Laboratories) for 30 min at room temperature. Slides were washed and incubated with a tyramide signal amplification (TSA) reagent (PerkinElmer, Waltham, MA; 1:50) for 10 min at room temperature. Slides were washed and incubated with streptavidin-488 (Life Technologies, 1:500), Cy3-anti-SMA (1A4 clone, Sigma, 1:1,000), and DRAQ5 (Thermo Fisher Scientific, 1:1,000) for 1 hr at room temperature. Slides were then mounted with Prolong Gold (Life Technologies) to minimize photobleaching, allowed to cure overnight, and imaged using a Nikon TE2000 C1 laser-scanning confocal microscope with a 20× oil objective. Cropped FOV (512 × 512 pixels) encompassing the collaterals in each region were randomized and de-identified. The pericollateral region was outlined (25 μm around the vessel), and pericollateral Mac3^+^ nuclei were counted in Fiji.^90^

F4/80 staining was performed similarly as previously described.^39^ Sections (5-μm thickness) of paraffin-embedded muscle from the muscular and saphenous regions were rehydrated and subjected to enzyme-mediated antigen retrieval for 15 min in 4-(2-hydroxyethyl)-1-piperazineethanesulfonic acid (HEPES)-buffered saline containing 0.05% trypsin and 10 μg/mL proteinase K. Sections were then quenched of endogenous peroxidase activity with a 30-min incubation in 3% H_2_O_2_. Slides were washed and incubated with anti-mouse CD16/CD32 Fc blocker (BD Biosciences, San Jose, CA; 1:100) for 30 min, and then blocked in PBS with 2% BSA and 5% normal goat serum for 1 hr. Endogenous biotin was also blocked, using an avidin-biotin-blocking kit as per the manufacturer’s instructions (Thermo Fisher Scientific, 004303). Slides were then incubated overnight at 4°C with a rat anti-F4/80 antibody (CI:A3-1 clone, Bio-Rad, 1:100). Following overnight incubation, slides were washed and incubated with a biotinylated sheep-anti-rat antibody (Abcam, ab6851, 1:200) for 1 hr at room temperature. Following secondary antibody incubation, slides were incubated in streptavidin-conjugated horseradish peroxidase (Tyramide Signal Amplification kit, PerkinElmer, Waltham, MA; 1:200) for 1 hr at room temperature. Slides were washed and incubated with a TSA reagent (PerkinElmer, Waltham, MA; 1:50) for 10 min at room temperature. Slides were washed and incubated with streptavidin-647 (Thermo Scientific, 1:200), Cy3-anti-SMA (1A4 clone, Sigma, 1:1,000), and Sytox Green (Thermo Scientific, 10 nM). Slides were then mounted with Prolong Diamond (Life Technologies) to minimize photobleaching, allowed to cure overnight, and imaged using a Nikon TE2000 C1 laser-scanning confocal microscope with a 20× oil objective. Cropped FOV (512 × 512 pixels) encompassing the collaterals in each region were randomized and de-identified. The pericollateral region was outlined (25 μm around the vessel), and pericollateral F4/80^+^ nuclei were counted in Fiji.

### Cross-Sectional Analysis for Gastrocnemius Muscle Morphology

For H&E analysis, sections (5-μm thickness) of paraffin-embedded muscle from the gastrocnemius muscle were H&E labeled. Individual FOV were imaged with a 10× objective on a Zeiss inverted microscope (Zeiss Axioskop) with a CCD camera (Quantifier, Optronics). Individual FOV were montaged together (Photoshop CS2, Adobe Systems). All montaged images were randomized and de-identified prior to analysis. Muscle areas were manually outlined using Fiji. Tissue composition was classified into viable and non-viable, which were defined as follows.

Viable: fibers are present and have centrally located nuclei (regenerating) or fibers are comparable in size, organization, and structure to unligated control with peripheral nuclei (mature).^8^
Non-viable: fibers lack nuclei, are rounded and dilated in appearance, have weak eosinophilic cytoplasm (necrotic), or there is a minimal presence of myoblasts and dominant fibrous matrix and adipose tissue (fibro-adipose).^8^

For Masson trichrome analysis, sections (5-μm thickness) of paraffin-embedded muscle from the muscular and saphenous regions were stained with Masson trichrome (iron hematoxylin, aniline blue, and Biebrich scarlet-acid fuchsin). Individual FOV were imaged using a Zeiss Axioskop transmitted light microscope with a 4× objective and a Jenoptik Gryphax camera, and then they were montaged together (Photoshop CS2, Adobe Systems). The tissue sections were outlined in the resulting images, and the pixels within the outline were analyzed with a R script written by the authors to assess the number of pixels with a significantly greater (>170%) blue than red intensity (indicating fibrotic tissue)^91^ in order to semiquantitatively assess the extent of fibrosis in each section.

### Cross-Sectional Analysis for Gastrocnemius Capillary Density

Sections (5-μm thickness) of paraffin-embedded gastrocnemius muscles were deparaffinized, rehydrated, then blocked in Carbofree blocking solution (1:10, Vector Laboratories). Slides were then incubated with fluorophore-conjugated primary antibody (isolectin-IB4-AlexFluor-647, 1:200, Life Technologies) overnight at 4°C. Nuclei were counterstained with Sytox green (500 μM, Life Technologies). Slides were washed and sealed with Prolong Gold (Life Technologies) to minimize photobleaching. Individual FOV were imaged with a Nikon TE2000 C1 laser-scanning confocal microscope with a 10× objective and the same imaging parameters for all FOV. FOV were then montaged together using Photoshop CS2 (Adobe Systems). Muscle areas were manually outlined using Fiji and classified as either viable or non-viable tissue. The numbers of capillaries (Isolectin-B4^+^ vessels <25 μm^2^ in diameter) and muscle area (identified from autofluorescence) were determined in each montaged image view using a semi-automated Fiji image analysis.

### Human Patient Population and Plasma Collection

All protocols were approved by the Institutional Review Board at the University of Virginia. Plasma was collected from an equal number of patients exhibiting intermittent claudication or as risk factor (e.g., diabetic, hypertensive, hyperlipidemic, and smoker) controls (n = 25/group). A diagnosis of PAD with intermittent claudication was based on having one of the following: (1) an ABI < 0.9; (2) a previous peripheral vascular intervention; or (3) an abnormal TBI, without any corresponding foot ulcers or resting pain. Total RNA was purified from 200 μL plasma using the miRCURY Biofluids RNA isolation kit (Exiqon, 300112). For reverse transcription (Applied Biosystems, 4366596) and miR-199a-5p detection, TaqMan miRNA assays (Applied Biosystems, 4427975) were used. RT-PCR was performed using the SensiMix II probe kit (Bioline) on an CFX96 detection system (Bio-Rad). Gene expression was normalized to RNU6 (Applied Biosystems, 4427975). Relative expression was determined with the comparative 2^ΔΔCt^ method and is represented as the log_2_ fold change. The log_2_ fold change data were normally distributed as determined by a Shapiro-Wilk test was tested for statistical significance using a two-sided Student’s t test.

### C2C12 Viability Assay

C2C12 myoblasts were plated at 1 × 10^4^ cells/well in a 96-well plate, and they were transfected with 20 nM scramble or miR-199a-5p LNA inhibitors (Exiqon) using siPORT NeoFX transfection agent (AM4510, Thermo Fisher Scientific), according to the manufacturer’s instructions. At 24 hr after transfection, cell culture medium was changed to serum starvation medium (209-250, Cell Applications) supplemented with tetrazolium salt WST-1/ECS solution (K301, BioVision Technologies, Milipitas, CA). Cells were incubated in a 2% oxygen chamber (BioSpherix, Lacona, NY) under hypoxia serum starvation (HSS) conditions for 6 hr. This time point was chosen as previous studies have shown significant C2C12 cell death under HSS conditions at later time points.^23^ After 6 hr, cell viability was assessed as the resulting tetrazolium salt cleavage to formazan by mitochondrial dehydrogenases by measuring absorbance at 450 nm.

### Statistical Analyses

All results are reported as mean ± SEM, unless otherwise noted. All data were first tested for normality using a Shapiro-Wilk test followed by equal variance. Statistical significance was then assessed by a Student’s t test or a two-way ANOVA followed by a Holm-Sidak multiple comparisons test, unless otherwise noted (SigmaStat 3.5, Systat). Significance was assessed at p < 0.05.

## Author Contributions

Conceptualization, J.L.H. and R.J.P.; Methodology, J.L.H. and R.J.P.; Investigation, J.L.H., C.M.G., S.P.M., and J.S.; Formal Analysis, J.L.H., C.M.G., and R.J.P.; Resources, B.H.A.; Writing – Original Draft, J.L.H. and R.J.P.; Writing – Review & Editing, J.L.H., C.M.G., S.P.M., J.S., B.H.A., and R.J.P.; Visualization, J.L.H. and R.J.P.; Supervision, B.H.A. and R.J.P.; Funding Acquisition, J.L.H., B.H.A., and R.J.P.

## Conflicts of Interest

The authors declare no competing interests.

## Supplemental Information

Document S1. Figures S1–S11 and Tables S1 and S2

Document S2. Article plus Supplemental Information
