# Supplementary material for: Exposure of Endothelium to Biomimetic Flow Waveforms Yields Identification of miR-199a-5p as a Potent Regulator of Arteriogenesis
Source: Mol Ther Nucleic Acids. 2018 Aug 8;12:829–44. doi: 10.1016/j.omtn.2018.08.001 (PMC6118158; doi:10.1016/j.omtn.2018.08.001)
Supplement: Document S1. Figures S1–S11 and Tables S1 and S2 [file mmc1.pdf]

OMTN, Volume 12

Supplemental Information

Exposure of Endothelium to Biomimetic Flow Waveforms Yields Identification of miR-199a-5p as a Potent Regulator of Arteriogenesis

Joshua L. Heuslein, Catherine M. Gorick, Stephanie P. McDonnell, Ji Song, Brian H. Annex, and Richard J. Price

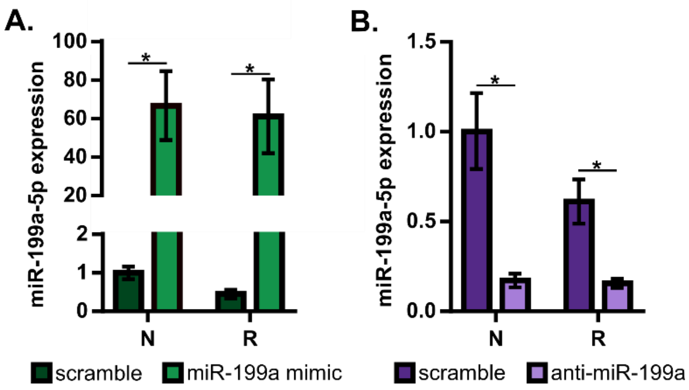

Figure S1. Validation of altered miR-199a-5p expression in transfected HUVECs.
A, B Bar graph of relative miR-199a expression 6-hours after simulated FAL in HUVECs transfected with (A) miR-199a mimic, (B) anti-miR-199a or the respective scramble controls and subjected to non-reversed (N) or reversed (R) flow waveforms as outlined in Figure 1*B* (n=4). *p<0.05, Student's *t*-test. Data are mean ± SEM.

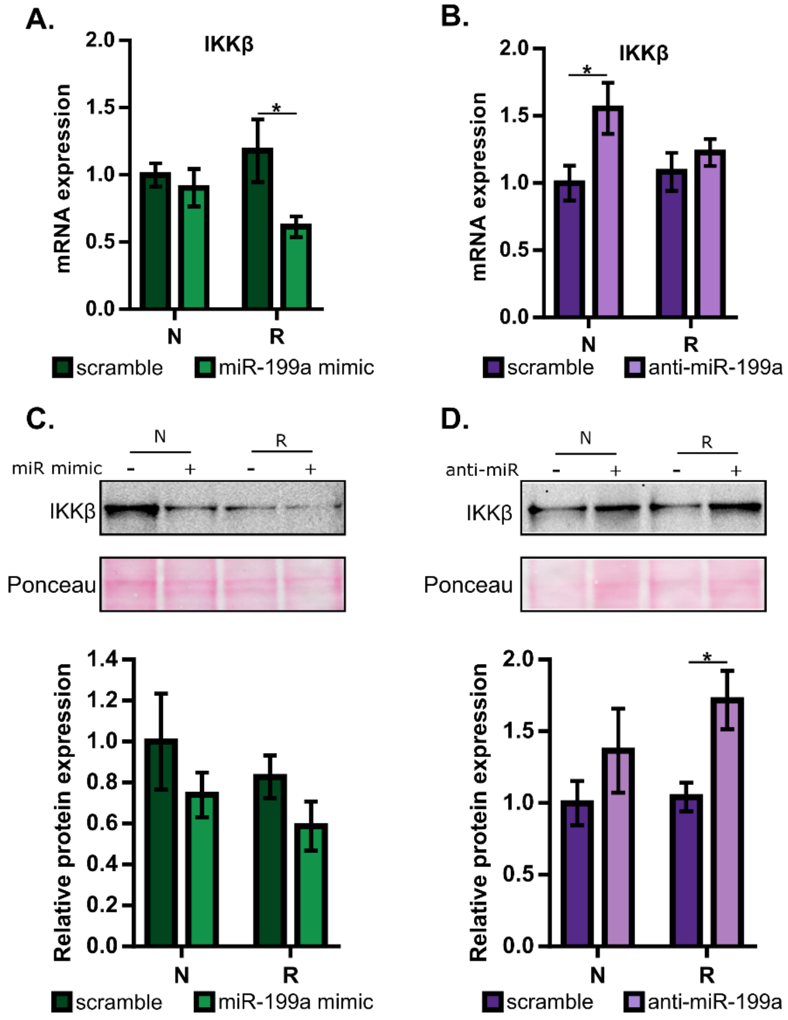

Figure S2. MicroRNA-199a modulation alters IKKβ expression .

A, B Bar graphs of relative IKKβ mRNA expression in transfected HUVECs subjected to non-reversed (N) or reversed (R) flow waveforms, 6-hr after simulated FAL. (n=4). *p<0.05, Student's *t*-test. **C**, **D** Bar graphs of relative IKKβ protein expression (MW~ 85kDa) expression in transfected HUVECs 6-hr after simulated FAL. (n=4). Ponceau staining was used for normalization of total protein. *p<0.05, Student's *t*-test. Data are mean ± SEM.

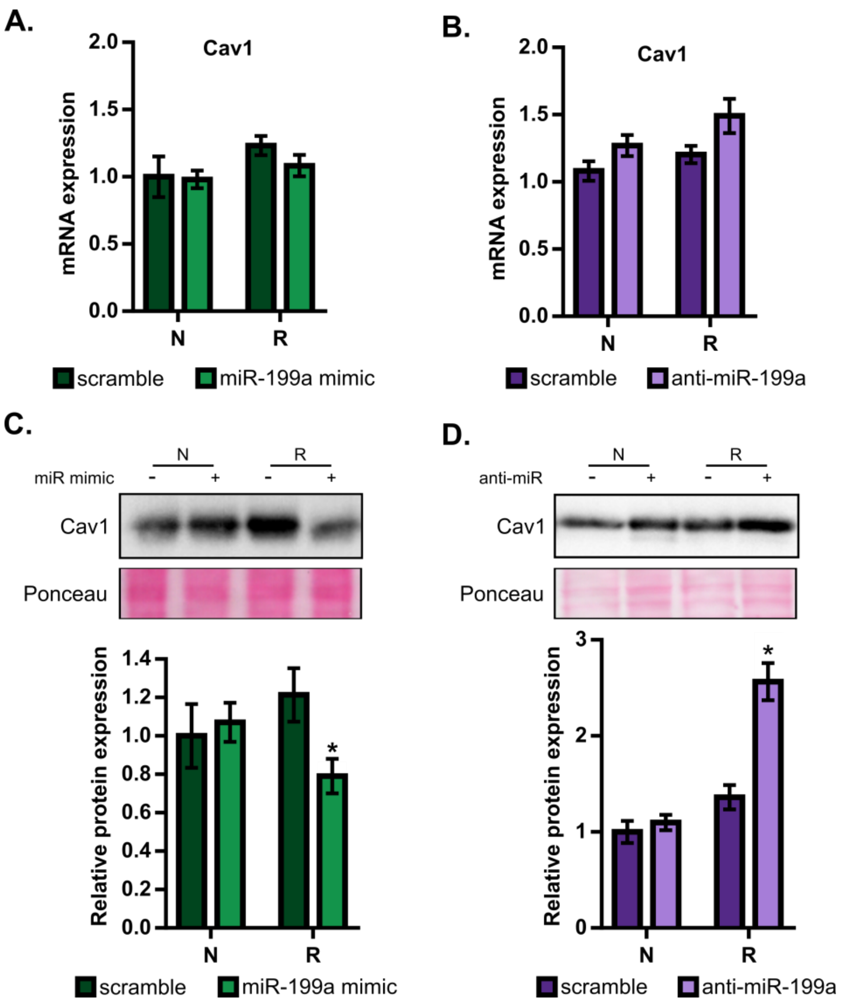

Figure S3. Altered miR-199a expression regulates Cav1 protein expression.
A, B Bar graphs of relative caveolin-1 (Cav1) mRNA expression in transfected HUVECs subjected to non-reversed (N) or reversed (R) flow waveforms, 6-hr after simulated FAL. (n=4). Student's *t*-test. **C**, **D** Bar graphs of relative Cav1 protein expression (MW~ 22kDa) expression in transfected HUVECs 6-hr after simulated FAL. (n=4). Ponceau staining was used for normalization of total protein. *p<0.05, Student's *t*-test. Data are mean ± SEM.

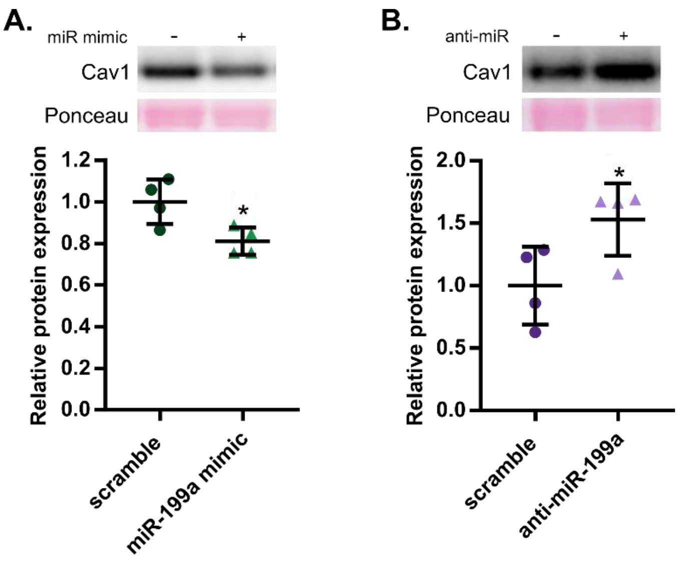

Figure S4. MicroRNA-199a negatively regulates the expression of caveolin-1 in-vivo.
A, B Bar graphs of relative caveolin-1 (Cav1, MW~22kDa) expression in the gracilis muscle 7 days post FAL in Balb/c mice injected with (A) miR-199a-5p mimic, (B) anti-miR-199a-5p, or the respective scramble control. (n=4). Ponceau staining was used for normalization of total protein.. *$p<0.05$, Student's *t*-test. Data are mean ± SEM.

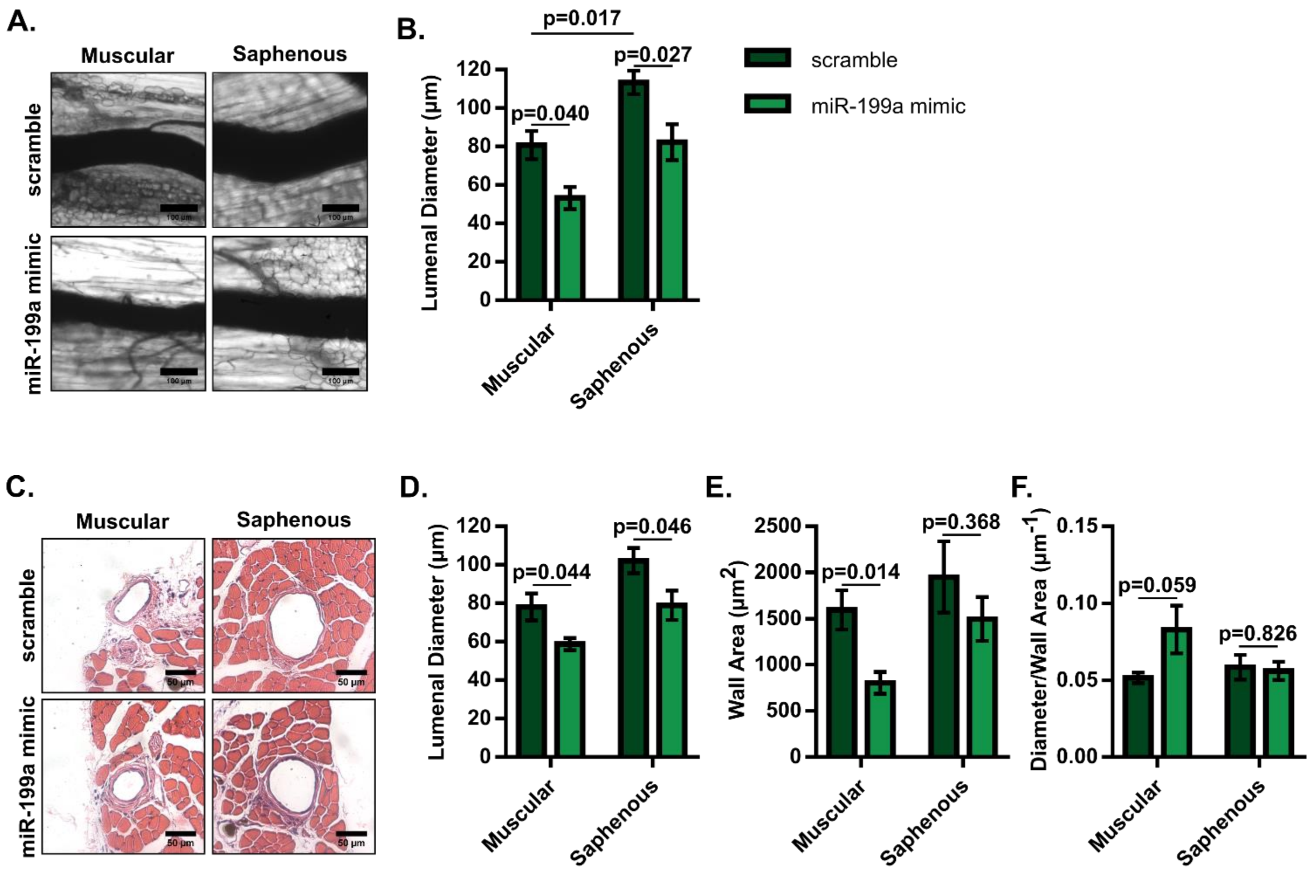

Figure S5. Regional analysis of Balb/c mice treated with miR-199a mimic after FAL.

A, Representative whole mount vascular cast images of collateral artery regions in scramble or miR-199a mimic treated Balb/c mice 21 days post-FAL (Scale bar = 100µm**)**. **B,** Bar graph of lumenal diameter for each group.*p<0.001 vs. unligated, two-way ANOVA followed by Holm-Sidak test for multiple comparisons. **C,** Representative H&E stained cross-sections of collateral arteries regions (n=5-6 for mimic and scramble groups respectively). **D-F,** Bar graphs of lumenal diameter, wall area, and diameter per wall area ratio from H&E stained cross-sections (n=5-6 for mimic and scramble groups respectively); Student's t-test. Data are mean ± SEM.

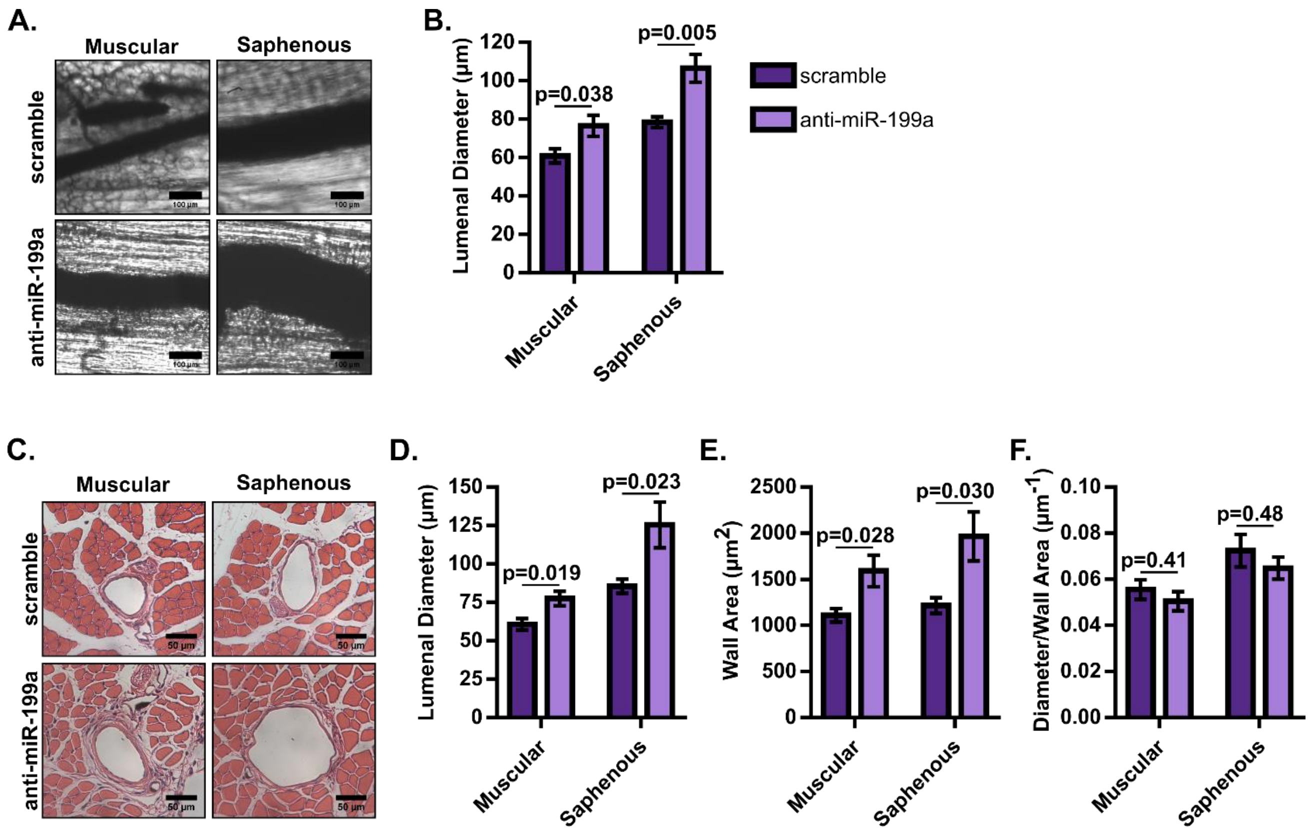

Figure S6. Regional analysis of Balb/c mice treated with anti-miR-199a after FAL.
A, Representative whole mount vascular cast images of collateral artery regions in scramble or anti-miR-199a treated Balb/c mice 21 days post-FAL (Scale bar=100µm). **B,** Bar graph of lumenal diameter for each group (n=6). *p<0.001 vs. unligated, two-way ANOVA followed by Holm-Sidak test for multiple comparisons. **C,** Representative H&E stained cross-sections of collateral arteries regions (n=6). **D-F,** Bar graphs of lumenal diameter, wall area, and diameter per wall area ratio from H&E stained cross-sections (n=6); Student's t-test. Data are mean ± SEM.

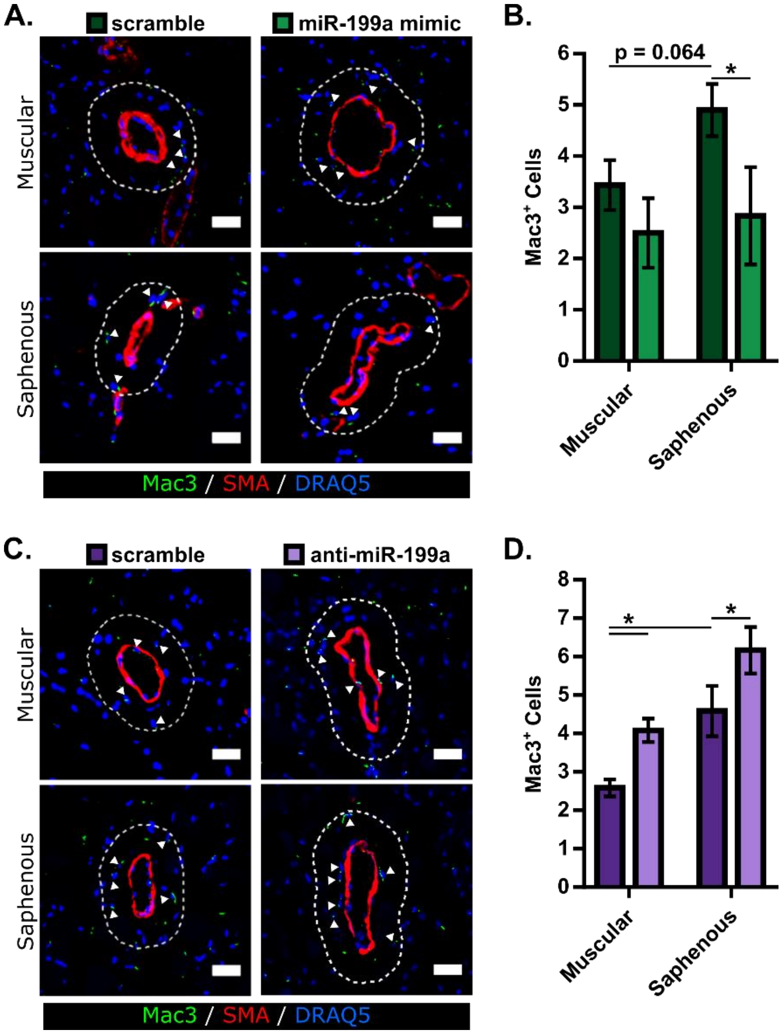

Figure S7. Regional pericollateral macrophage recruitment is modulated by miR-199a.
A, Representative cross-sections of gracilis collateral artery regions 7 days post-FAL immunolabeled for macrophage marker, Mac3 (green), smooth muscle alpha actin (SMA, red), and nuclei (DRAQ5, blue) in Balb/c mice treated with miR-199a mimic or scramble mimic. Dotted line indicates the pericollateral region (25μm from vessel wall) used for quantification. Arrowheads indicate Mac3$^+$ cells (Scale bar=25μm). **B,** Bar graph of pericollateral Mac3$^+$ cells (n=4-5 for miR-199a mimic and scramble, respectively). *p<0.05, two-way ANOVA followed by a Holm-Sidak test for multiple comparisons. **C,** Immunolabeled gracilis collateral artery regions, as in (A), in Balb/c mice treated with anti-miR-199a or scramble oligonucleotide. **D,** Bar graph of pericollateral Mac3$^+$ cells (n=3). *p<0.05, two-way ANOVA followed by a Holm-Sidak test for multiple comparisons. Data are mean ± SEM.

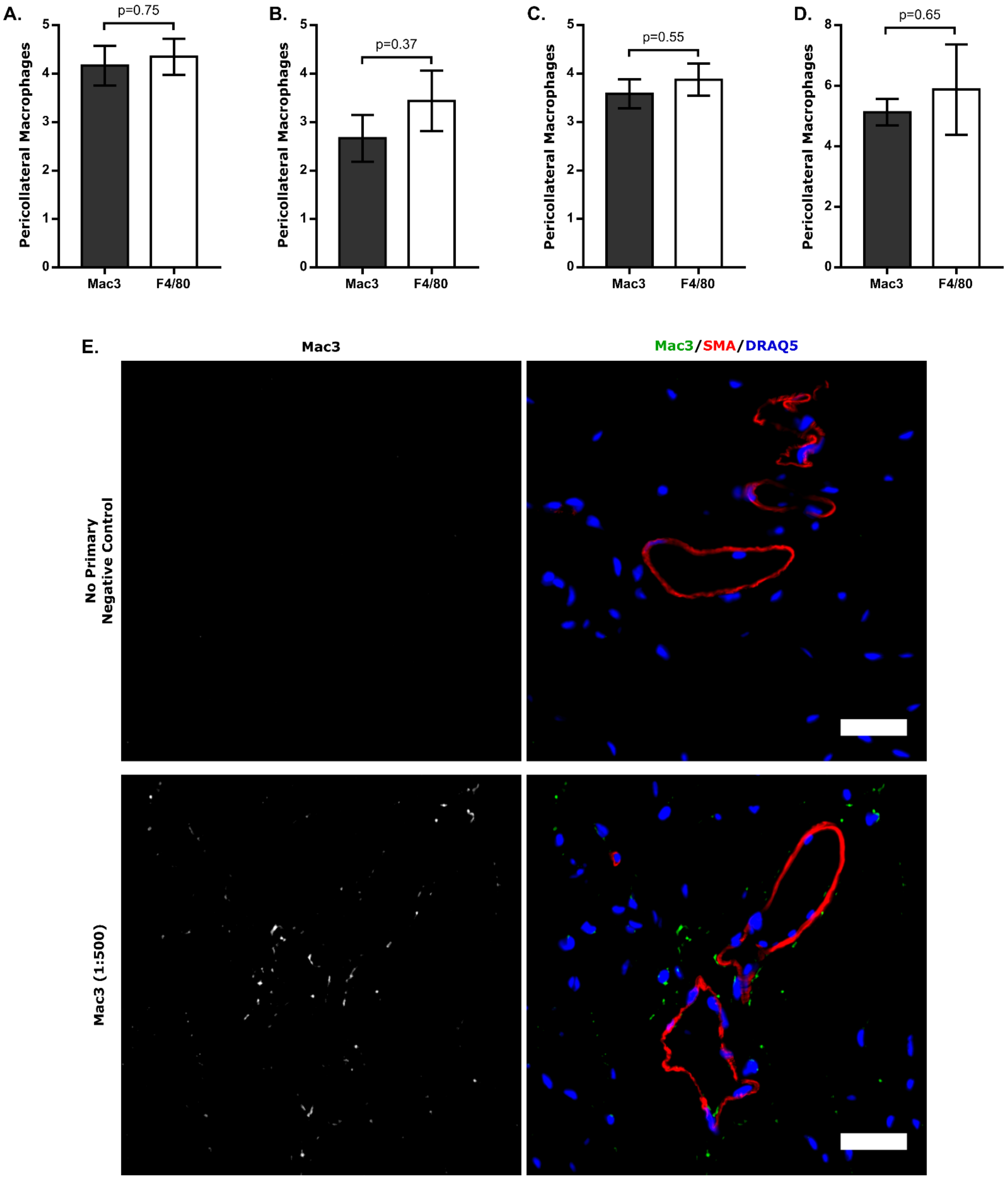

Figure S8. F4/80+ pericollateral macrophages exhibit similar trend as Mac3

A-D, Counts of $Mac3^{+}$ or $F4/80^{+}$ pericollateral macrophages in mice treated with (A) scramble mimic (n=5) (B) miR-199a mimic (n=4) (C) scramble anti-miR (n=3) or (D) anti-miR-199a (n=3) oligonucleotides, day 7 post-FAL. There is no statistical difference between Mac3 or F4/80 in any of the groups. Student's t-test. Data are mean ± SEM. **E,** Cross-sections of gracilis collateral artery regions 7 days post-FAL immunolabeled without primary antibody (negative control) or Mac3 (green, 1:500). Sections were counterstained with smooth muscle alpha actin (SMA, red) and DRAQ5 (nuclei, blue) (Scale bar=25μm).

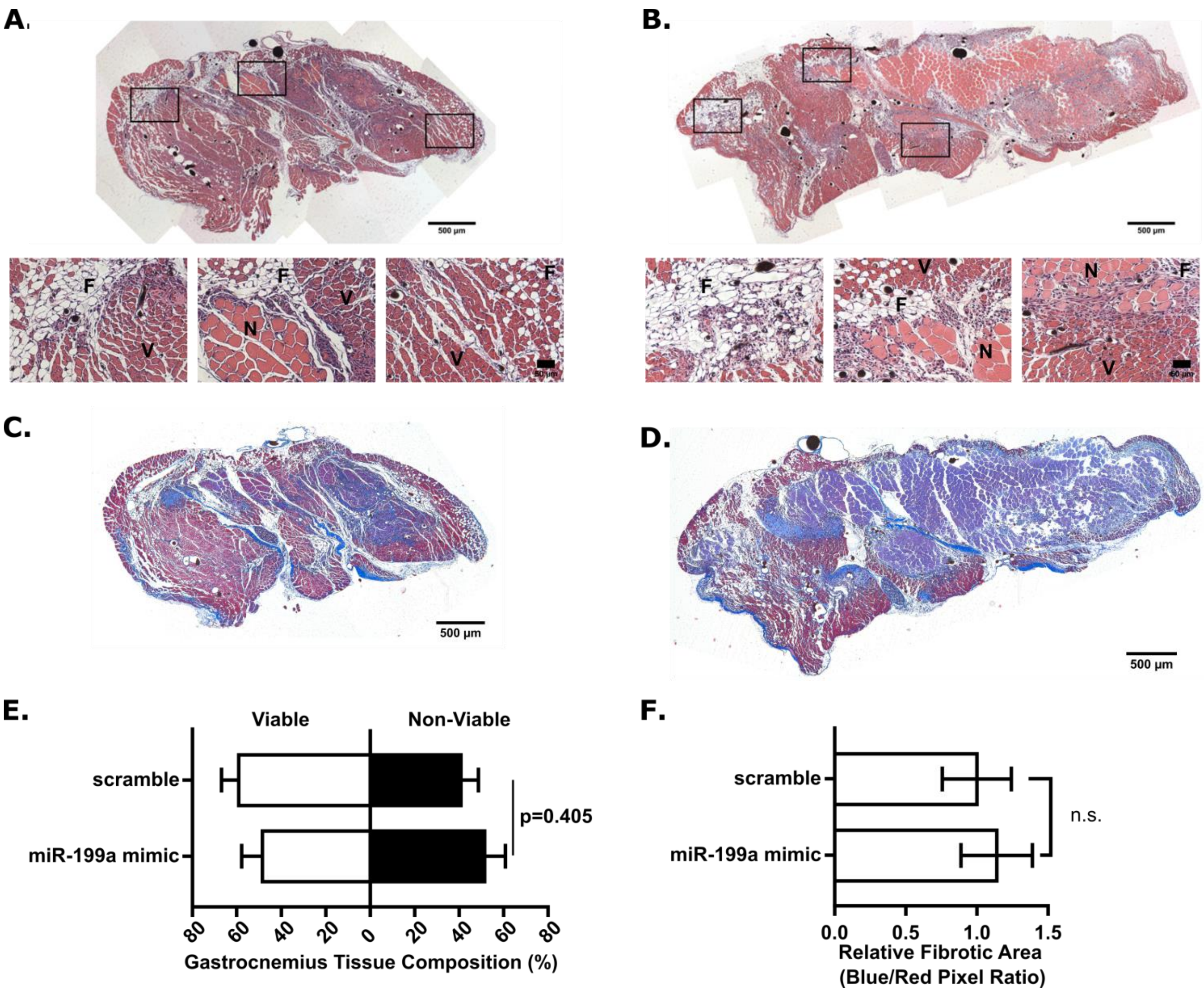

Figure S9. Gastrocnemius muscle composition is not further impaired with miR-199a overexpression following FAL

A-B, Representative images of H&E staining of gastrocnemius muscle for ligated limb of Balb/c mice treated with scramble (A) or miR-199a mimic (B) (Scale bar=500µm, inset scale bar=50µm). V=viable muscle, N=necrotic tissue, and F=fibro-adipose tissue. **C-D,** Representative images of Masson Trichrome stained gastrocnemius muscles from ligated limb of Balb/c mice treated with scramble (C) or miR-199a mimic (D) (Scale bar=500µm). Blue staining is indicative of collagen/fibrotic content whereas red staining indicates healthy tissue. **E,** Bar graph of the percentage of gastrocnemius muscle that is viable (white) or non-viable (black) at day 21 post-FAL in each group (n=6). **F,** Bar graph of the relative fibrotic area in gastrocnemius muscle at day 21 post-FAL in each group (n=6). n.s. = not significant (p>0.05), Student's *t*-test. Data are mean ± SEM.

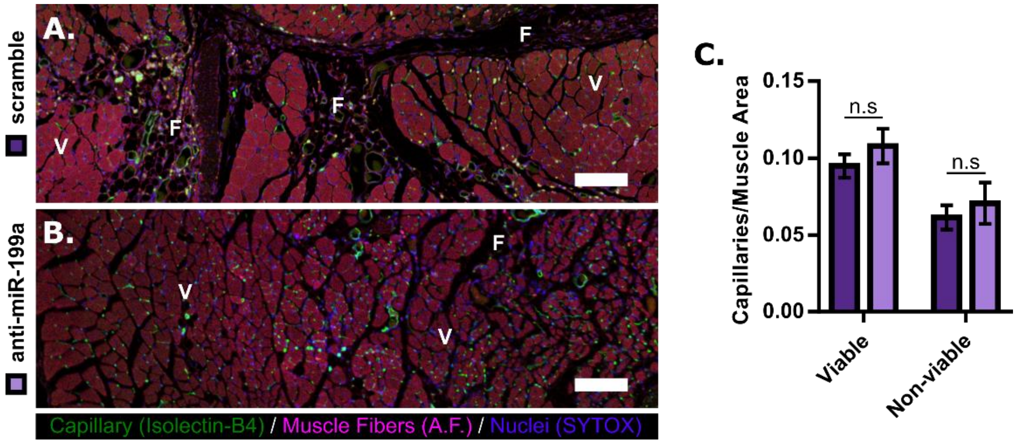

Figure S10. MicroRNA-199a inhibition does not affect capillary density in the gastrocnemius muscle of FAL-operated Balb/c mice.

A-B, Representative photomerged images of gastrocnemius muscle for ligated limb of Balb/c mice treated with scramble (A) or anti-miR-199a (B) locked nucleic acid oligonucleotides immediately after FAL (Scale bar=500μm). Tissues are immunolabeled for capillaries (green, isolectin-B4), muscle fibers (red, autofluorescence [A.F]), and nuclei (blue, SYTOX). **C,** Capillary density was determined for both groups in viable tissue (mature and regenerating muscle, V) and non-viable tissue (necrotic and fibro-adipose [F]) (n=4-5, for ctrl and anti-miR-199a, respectively). n.s = not significant, Student's t-test. Data are mean ± SEM.

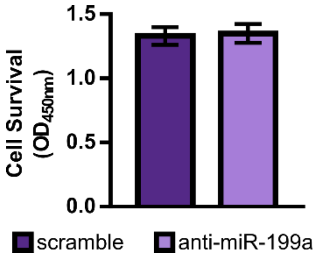

Figure S11. miR-199a inhibition does not improve C2C12 survival under hypoxic conditions in-vitro. Twenty-four hours after transfection with 30nM scramble or anti-miR-199a-5p oligonucleotides, C2C12 myoblasts were exposed to 6 hours of hypoxia (2% O2) and serum starvation conditions and assess for cell viability (n=4). Student's *t*-test. Data are mean ± SEM.

Table S1. *Differential microRNA gene expression in ECs exposed to arteriogenesis-biomimetic shear stress waveforms.*

Symbol	Name	NvC FC	RvC FC	RvN FC	-log$_{10}$(FDR)
MIR146A	microRNA 146a	0.99	0.52	0.52	2.00
MIR199A2	microRNA 199a-2	0.95	0.72	0.76	1.89
MIR34A	microRNA 34a	0.92	1.00	1.09	1.50
MIR100	microRNA 100	0.91	0.69	0.76	1.49
MIR216A	microRNA 216a	0.93	0.74	0.79	1.26
MIR93	microRNA 93	1.05	0.84	0.80	1.19
MIR17HG	miR-17-92 cluster host gene	1.01	0.81	0.80	0.97
MIR126	microRNA 126	0.85	0.70	0.82	0.87
MIR224	microRNA 224	1.07	0.73	0.68	0.87
MIR382	microRNA 382	0.95	1.22	1.28	0.87
MIR181B1	microRNA 181b-1	0.99	0.81	0.82	0.83
MIR155	microRNA 155	1.23	0.77	0.63	0.76
MIR145	microRNA 145	1.07	1.18	1.11	0.70
MIR103A1	microRNA 103a-1	0.87	0.90	1.04	0.70
MIR105-2	microRNA 105-2	1.12	1.23	1.10	0.69
MIR32	microRNA 32	1.00	0.76	0.76	0.64
MIR21	microRNA 21	1.05	0.77	0.73	0.59
MIR219-2	microRNA 219-2	0.97	1.10	1.13	0.58
MIR22HG	MIR22 host gene	1.07	1.12	1.05	0.58
MIR181B2	microRNA 181b-2	0.96	0.84	0.87	0.57
MIR30E	microRNA 30e	0.93	0.88	0.94	0.56
MIR485	microRNA 485	0.99	1.12	1.13	0.55
MIR127	microRNA 127	0.96	0.90	0.93	0.54
MIR10A	microRNA 10a	0.81	0.78	0.97	0.51
MIRLET7C	microRNA let-7c	0.96	0.82	0.85	0.51
MIR429	microRNA 429	1.01	1.11	1.11	0.50
MIR23A	microRNA 23a	1.02	0.84	0.83	0.48
MIR199A1	microRNA 199a-1	1.12	0.89	0.79	0.45
MIR106B	microRNA 106b	0.98	0.91	0.94	0.41
MIRLET7I	microRNA let-7i	1.03	1.11	1.08	0.39
MIR133A1	microRNA 133a-1	0.97	1.11	1.14	0.38
MIR29A	microRNA 29a	1.13	0.78	0.69	0.37
MIR27A	microRNA 27a	0.85	0.85	1.01	0.37
MIR622	microRNA 622	0.90	1.21	1.36	0.36
MIR29B2	microRNA 29b-2	1.07	0.86	0.81	0.35
MIR143	microRNA 143	1.05	0.92	0.88	0.35
MIR30C1	microRNA 30c-1	1.00	0.89	0.90	0.32
MIR9-2	microRNA 9-2	1.00	1.07	1.07	0.31
MIRLET7F1	microRNA let-7f-1	1.07	1.16	1.09	0.30
MIR24-2	microRNA 24-2	0.93	0.88	0.95	0.29
MIR154	microRNA 154	0.97	1.24	1.27	0.28
MIR26A1	microRNA 26a-1	0.94	0.90	0.96	0.26
MIR25	microRNA 25	0.85	0.91	1.08	0.26
MIR15B	microRNA 15b	0.77	0.91	1.18	0.25
MIR320C1	microRNA 320c-1	1.11	1.08	0.97	0.25
MIR124-2	microRNA 124-2	1.00	0.95	0.95	0.25

MIR214	microRNA 214	0.93	0.93	1.00	0.24
MIR206	microRNA 206	1.04	1.06	1.02	0.24
MIR186	microRNA 186	0.94	1.11	1.18	0.24
MIR365A	microRNA 365a	0.78	0.91	1.18	0.24
MIR34C	microRNA 34c	0.86	0.93	1.08	0.24
MIR181A1	microRNA 181a-1	0.99	0.92	0.93	0.23
MIR195	microRNA 195	1.06	0.93	0.88	0.22
MIR221	microRNA 221	0.95	1.14	1.21	0.22
MIR99B	microRNA 99b	0.96	0.93	0.96	0.21
MIR122	microRNA 122	1.09	1.07	0.97	0.21
MIR377	microRNA 377	0.98	1.07	1.10	0.20
MIR137HG	MIR137 host gene	1.00	1.08	1.08	0.20
MIR194-1	microRNA 194-1	0.92	1.10	1.20	0.20
MIR24-1	microRNA 24-1	1.14	0.90	0.80	0.19
MIR210	microRNA 210	1.07	0.95	0.89	0.18
MIR184	microRNA 184	0.99	0.94	0.94	0.17
MIR105-1	microRNA 105-1	1.24	1.24	0.99	0.17
MIR520C	microRNA 520c	0.99	0.91	0.91	0.16
MIR15A	microRNA 15a	0.80	0.87	1.10	0.16
MIR23B	microRNA 23b	0.85	0.91	1.07	0.16
MIR376C	microRNA 376c	0.92	1.05	1.15	0.16
MIR218-1	microRNA 218-1	0.85	0.91	1.07	0.16
MIR492	microRNA 492	1.03	1.08	1.05	0.15
MIR101-2	microRNA 101-2	1.03	1.05	1.02	0.15
MIR28	microRNA 28	1.01	1.09	1.08	0.14
MIR200A	microRNA 200a	0.98	1.08	1.10	0.14
MIR320A	microRNA 320a	0.92	1.06	1.15	0.14
MIR323A	microRNA 323a	1.08	1.06	0.98	0.14
MIR147A	microRNA 147a	0.71	0.94	1.33	0.14
MIR26B	microRNA 26b	0.94	0.96	1.01	0.13
MIR16-1	microRNA 16-1	0.82	0.96	1.17	0.13
MIRLET7A1	microRNA let-7a-1	1.01	0.93	0.93	0.13
MIR29C	microRNA 29c	1.20	1.10	0.92	0.13
MIR136	microRNA 136	1.03	1.08	1.04	0.12
MIR135A1	microRNA 135a-1	1.00	0.93	0.94	0.12
MIR7-3HG	MIR7-3 host gene	0.89	0.95	1.06	0.12
MIR219-1	microRNA 219-1	0.96	1.07	1.12	0.11
MIR208A	microRNA 208a	0.97	1.06	1.09	0.11
MIR153-1	microRNA 153-1	1.01	0.95	0.94	0.11
MIR183	microRNA 183	0.98	0.95	0.97	0.11
MIR199B	microRNA 199b	0.87	0.93	1.06	0.11
MIR519A2	microRNA 519a-2	1.00	0.96	0.96	0.10
MIR99A	microRNA 99a	0.93	0.95	1.02	0.10
MIR7-2	microRNA 7-2	0.61	1.04	1.71	0.10
MIRLET7A3	microRNA let-7a-3	1.02	0.93	0.91	0.10
MIR217	microRNA 217	0.98	0.88	0.90	0.10
MIR144	microRNA 144	0.91	1.05	1.16	0.10
MIR139	microRNA 139	1.02	1.04	1.02	0.09
MIRLET7F2	microRNA let-7f-2	1.00	1.08	1.09	0.09
MIR410	microRNA 410	0.96	0.95	0.99	0.09

MIR211	microRNA 211	0.77	0.94	1.23	0.09
MIR185	microRNA 185	0.98	0.96	0.98	0.09
MIR412	microRNA 412	1.01	1.05	1.04	0.09
MIR197	microRNA 197	0.93	0.95	1.02	0.09
MIR188	microRNA 188	1.08	1.03	0.95	0.09
MIR33A	microRNA 33a	0.93	0.93	1.00	0.09
MIR519A2	microRNA 519a-2	1.00	0.92	0.92	0.08
MIR302A	microRNA 302a	0.94	0.97	1.02	0.08
MIR30D	microRNA 30d	0.95	1.04	1.10	0.08
MIRLET7E	microRNA let-7e	0.89	0.93	1.04	0.08
MIR96	microRNA 96	0.98	1.05	1.07	0.08
MIR16-2	microRNA 16-2	0.95	0.96	1.01	0.08
MIR181A2	microRNA 181a-2	0.86	0.93	1.09	0.08
MIR134	microRNA 134	1.00	1.04	1.04	0.07
MIR495	microRNA 495	1.16	0.96	0.83	0.07
MIR516B2	microRNA 516b-2	0.97	0.94	0.97	0.07
MIR30A	microRNA 30a	0.91	1.06	1.16	0.07
MIRLET7D	microRNA let-7d	0.94	1.04	1.11	0.07
MIR223	microRNA 223	1.27	0.97	0.77	0.07
MIR30C2	microRNA 30c-2	0.99	0.92	0.93	0.07
MIR124-1	microRNA 124-1	1.04	1.05	1.00	0.07
MIR193A	microRNA 193a	0.96	1.04	1.08	0.07
MIR137	microRNA 137	1.18	1.04	0.88	0.07
MIR600HG	MIR600 host gene	1.14	0.96	0.85	0.07
MIR218-2	microRNA 218-2	0.88	0.95	1.09	0.07
MIR9-3	microRNA 9-3	0.91	0.96	1.05	0.07
MIR187	microRNA 187	0.96	0.97	1.00	0.06
MIR202	microRNA 202	1.00	1.04	1.04	0.06
MIR1-1	microRNA 1-1	0.98	1.03	1.06	0.06
MIR203	microRNA 203	0.94	1.03	1.09	0.06
MIR339	microRNA 339	1.03	1.04	1.01	0.06
MIRLET7B	microRNA let-7b	1.00	0.97	0.97	0.06
MIR509-1	microRNA 509-1	1.02	1.03	1.01	0.06
MIR103A2	microRNA 103a-2	0.87	1.05	1.21	0.06
MIR26A2	microRNA 26a-2	1.04	1.06	1.01	0.06
MIR487A	microRNA 487a	1.12	1.04	0.93	0.06
MIR301A	microRNA 301a	1.09	0.96	0.89	0.06
MIR124-3	microRNA 124-3	1.03	0.95	0.93	0.06
MIR140	microRNA 140	0.98	0.97	0.98	0.06
MIR451A	microRNA 451a	0.98	1.03	1.05	0.06
MIR128-1	microRNA 128-1	0.98	1.02	1.05	0.06
MIR182	microRNA 182	0.92	0.96	1.05	0.06
MIR212	microRNA 212	0.90	0.97	1.07	0.06
MIR148A	microRNA 148a	0.92	1.03	1.12	0.05
MIR153-2	microRNA 153-2	1.01	0.98	0.97	0.05
MIR1247	microRNA 1247	0.95	0.98	1.03	0.05
MIR130B	microRNA 130b	1.00	1.03	1.03	0.05
MIR503	microRNA 503	1.07	0.97	0.91	0.05
MIR7-3	microRNA 7-3	0.97	0.97	1.00	0.05
MIR331	microRNA 331	1.03	1.04	1.01	0.05

MIR204	microRNA 204	0.96	1.03	1.08	0.04
MIR125A	microRNA 125a	0.90	0.97	1.07	0.04
MIR129-2	microRNA 129-2	1.11	0.97	0.87	0.04
MIR34B	microRNA 34b	0.94	1.03	1.10	0.04
MIR504	microRNA 504	1.20	1.03	0.86	0.04
MIR190A	microRNA 190a	0.90	1.02	1.14	0.04
MIRLET7A2	microRNA let-7a-2	1.03	0.96	0.93	0.04
MIR425	microRNA 425	1.28	0.97	0.76	0.03
MIR132	microRNA 132	0.92	0.97	1.06	0.03
MIR192	microRNA 192	1.06	1.03	0.97	0.03
MIR95	microRNA 95	0.97	1.02	1.05	0.03
MIR133B	microRNA 133b	1.04	1.02	0.98	0.03
MIR148B	microRNA 148b	1.05	0.97	0.92	0.03
MIR455	microRNA 455	0.95	0.98	1.04	0.03
MIRLET7BHG	MIRLET7B host gene	1.01	0.98	0.97	0.03
MIR215	microRNA 215	0.94	1.02	1.08	0.03
MIR194-2	microRNA 194-2	0.98	1.02	1.04	0.03
MIR19B2	microRNA 19b-2	0.72	1.02	1.42	0.03
MIR92A2	microRNA 92a-2	1.12	0.99	0.88	0.03
MIR30B	microRNA 30b	1.06	1.03	0.97	0.03
MIR27B	microRNA 27b	0.94	1.03	1.10	0.02
MIR130A	microRNA 130a	0.98	1.02	1.04	0.02
MIR149	microRNA 149	0.89	1.02	1.14	0.02
MIR138-1	microRNA 138-1	1.05	1.02	0.97	0.02
MIR135A2	microRNA 135a-2	0.96	0.98	1.03	0.02
MIR1-2	microRNA 1-2	1.06	0.98	0.93	0.02
MIR141	microRNA 141	1.01	0.98	0.97	0.02
MIR196A2	microRNA 196a-2	0.98	1.01	1.04	0.02
MIR423	microRNA 423	1.04	0.98	0.94	0.02
MIR516B1	microRNA 516b-1	1.06	0.98	0.92	0.02
MIR191	microRNA 191	1.01	0.99	0.97	0.02
MIR222	microRNA 222	1.03	0.98	0.95	0.02
MIR375	microRNA 375	0.96	1.01	1.06	0.01
MIR181C	microRNA 181c	1.10	1.01	0.92	0.01
MIR9-1	microRNA 9-1	1.02	1.01	0.99	0.01
MIR31HG	MIR31 host gene	0.99	1.01	1.02	0.01
MIRLET7G	microRNA let-7g	0.91	0.98	1.08	0.01
MIR196A1	microRNA 196a-1	0.93	1.01	1.09	0.01
MIR129-1	microRNA 129-1	0.97	0.99	1.02	0.01
MIR125B2	microRNA 125b-2	1.08	0.99	0.92	0.01
MIR31	microRNA 31	1.03	1.02	0.99	0.01
MIR326	microRNA 326	0.85	1.01	1.19	0.01
MIR296	microRNA 296	1.00	1.01	1.01	0.01
MIR142	microRNA 142	0.96	1.01	1.05	0.01
MIR138-2	microRNA 138-2	0.93	1.01	1.08	0.01
MIR152	microRNA 152	0.98	1.01	1.03	0.01
MIR125B1	microRNA 125b-1	0.97	0.99	1.02	0.01
MIR106A	microRNA 106a	0.99	1.01	1.02	0.01
MIR10B	microRNA 10b	1.01	1.01	1.00	0.01
MIR101-1	microRNA 101-1	0.89	0.99	1.12	0.01

MIR330	microRNA 330	1.01	1.01	1.00	0.01
MIR302B	microRNA 302b	1.11	1.01	0.91	0.01
MIR200B	microRNA 200b	1.03	1.00	0.97	0.01
MIR150	microRNA 150	0.99	1.01	1.01	0.01
MIR200C	microRNA 200c	0.99	1.01	1.01	0.01
MIR128-2	microRNA 128-2	0.97	0.99	1.03	0.00
MIR34A	microRNA 34a	0.99	0.72	0.73	0.00
MIR98	microRNA 98	1.05	1.00	0.96	0.00
MIR29B1	microRNA 29b-1	0.91	1.00	1.10	0.00
MIR299	microRNA 299	0.91	1.00	1.11	0.00
MIR107	microRNA 107	1.02	1.00	0.99	0.00
MIR494	microRNA 494	1.00	1.00	1.00	0.00
MIR205	microRNA 205	0.99	1.00	1.01	0.00

*FC = fold change. FDR = false discovery rate of RvC dataset.
N = non-reversed, R = reversed, C= control shear stress waveforms from Heuslein and Meisner et al.

Table S2. Patient demographics of human plasma samples.

Patient Information	Control (n=25)	PAD (n=25)	p-value
Minimum ABI	1.09 ± 0.12	0.75 ± 0.26	<0.0001
Male / Female	60% / 40%	52% / 48%	0.776
Age (years)	64.2 ± 7.7	64.8 ± 11.3	0.977
Smoker	68%	92%	0.074
Diabetic	40%	36%	0.999
Hypertensive	88%	96%	0.609
Hyperlipidemic	80%	85%	0.702

Data are mean ± SD. A Mann-Whitney U-test was used for minimum ankle-brachial index (ABI) and age data whereas categorical data was subjected to a Fisher's exact test to determine statistical differences between groups. $p<0.05$ is considered statistically significant.
