# Supplementary material for: Exposure of Endothelium to Biomimetic Flow Waveforms Yields Identification of miR-199a-5p as a Potent Regulator of Arteriogenesis
Source: Mol Ther Nucleic Acids. 2018 Aug 8;12:829–44. doi: 10.1016/j.omtn.2018.08.001 (PMC6118158; doi:10.1016/j.omtn.2018.08.001)
Supplement: Document S2. Article plus Supplemental Information [file mmc2.pdf]

Molecular Therapy
Nucleic Acids
Original Article

Exposure of Endothelium to Biomimetic Flow Waveforms Yields Identification of miR-199a-5p as a Potent Regulator of Arteriogenesis

Joshua L. Heuslein,[1,2] Catherine M. Gorick,[1] Stephanie P. McDonnell,[2] Ji Song,[1] Brian H. Annex,[2] and Richard J. Price[1,2]

[1]Department of Biomedical Engineering, University of Virginia, Charlottesville, VA, USA; [2]Robert M. Berne Cardiovascular Research Center, University of Virginia, Charlottesville, VA, USA

Arteriogenesis, the growth of endogenous collateral arteries bypassing arterial occlusion(s), is a fundamental shear stress-induced adaptation with implications for treating peripheral arterial disease (PAD). Nonetheless, endothelial mechano-signaling during arteriogenesis is incompletely understood. Here we tested the hypothesis that a mechanosensitive microRNA, miR-199a-5p, regulates perfusion recovery and collateral arteriogenesis following femoral arterial ligation (FAL) via control of monocyte recruitment and pro-arteriogenic gene expression. We have previously shown that collateral artery segments exhibit distinctly amplified arteriogenesis if they are exposed to reversed flow following FAL in the mouse. We performed a genome-wide analysis of endothelial cells exposed to a biomimetic reversed flow waveform. From this analysis, we identified mechanosensitive miR-199a-5p as a novel candidate regulator of collateral arteriogenesis. *In vitro*, miR-199a-5p inhibited pro-arteriogenic gene expression (IKKβ, Cav1) and monocyte adhesion to endothelium. *In vivo*, following FAL in mice, miR-199a-5p overexpression impaired foot perfusion and arteriogenesis. In contrast, a single intramuscular anti-miR-199a-5p injection elicited a robust therapeutic response, including complete foot perfusion recovery, markedly augmented arteriogenesis (>3.4-fold increase in segment conductance), and improved gastrocnemius tissue composition. Finally, we found plasma miR-199a-5p to be elevated in human PAD patients with intermittent claudication compared to a risk factor control population. Through our transformative analysis of endothelial mechano-signaling in response to a biomimetic amplified arteriogenesis flow waveform, we have identified miR-199a-5p as both a potent regulator of arteriogenesis and a putative target for treating PAD.

INTRODUCTION

Peripheral arterial disease (PAD) has become a global problem: it is estimated that over 202 million people worldwide have PAD.[1] PAD arises when atherosclerotic plaques block arteries in the lower limbs, thereby limiting blood flow to the distal tissue, ultimately leading to intermittent claudication or critical limb ischemia in severe cases. Many PAD patients are either not amenable to surgical intervention or receive little long-term benefit from surgery.[2] Revascularization strategies to stimulate the growth of new capillaries from preexisting vessels (i.e., angiogenesis) or lumenal expansion of pre-existing arteries (i.e., arteriogenesis) remain promising therapeutic options, despite their limited success to date.[3] The stimulation of angiogenesis is important in PAD, as capillary density is reduced in these patients;[2,4,5] however, it is also imperative to restore the driving pressure to the distal tissue via lumenal expansion (i.e., arteriogenesis) of collateral arteries bypassing the occlusion(s).[6–8] Arteriogenesis is well known to be stimulated by altered shear stress,[9] though endothelial mechano-signaling in response to this altered shear stress is incompletely understood.

We recently reported that a subset of collateral segments in the mouse hindlimb display remarkably permanent, amplified arteriogenesis after femoral arterial ligation (FAL).[10] Specifically, we showed that collateral artery segments exposed to both a 2-fold increase in shear stress magnitude and reversed flow direction (reversed flow) following FAL exhibit an ∼30% increase in lumenal diameter 12 weeks post-FAL compared to segments experiencing just a 2-fold increase in shear stress magnitude (non-reversed flow) (Figure 1A).[11] Moreover, by applying shear stress waveforms biomimetic of these *in vivo* hemodynamics to endothelial cells (ECs) *in vitro*, we were able to generate a direct, comprehensive mapping of EC mechanosensitive signaling to differential arteriogenesis responses.[11] Comparative analysis of EC mechano-signaling corresponding to these differential responses may enable the discovery of novel regulators of arteriogenesis.

To this end, mature microRNAs (miRNAs) are now well recognized as key regulators of vascular remodeling.[12,13] miRNAs are ∼22-nt,

Received 21 September 2017; accepted 1 August 2018;
https://doi.org/10.1016/j.omtn.2018.08.001.

Correspondence: Richard J. Price, PhD, Department of Biomedical Engineering, University of Virginia, Box 800759, Health System, Charlottesville, VA 22903, USA.
E-mail: rprice@virginia.edu

This is an open access article under the CC BY license (http://creativecommons.org/licenses/by/4.0/).

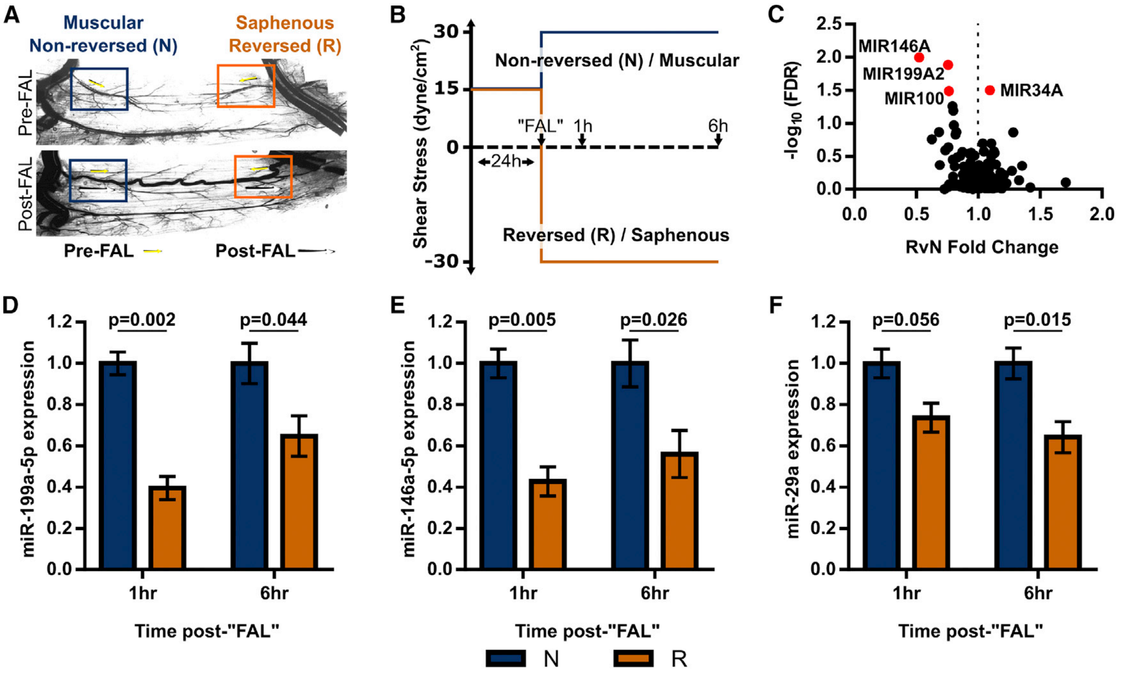

Figure 1. Endothelial Cell miRNA Expression Is Differentially Regulated by Shear Stress Waveforms Biomimetic of Collateral Artery Segments Exhibiting Varied Arteriogenic Responses

(A) Representative vascular cast images of gracilis adductor collaterals in BALB/c mice taken from unligated sham control (top) and day 21 post-femoral arterial ligation (FAL, bottom) groups. Arrows indicate direction and magnitude of blood flow pre- (yellow) and post- (white) FAL. The femoral artery is ligated just distal to the epigastric artery such that some collateral segments (muscular) experience a 2-fold increase in shear stress magnitude (non-reversed flow), while other segments (saphenous) are exposed to both a 2-fold increase in shear stress magnitude and reversed flow direction (reversed flow). Arteriogenesis is amplified in the flow-reversed, saphenous regions of collateral arteries. (B) Schematic depicting biomimetic waveforms applied to HUVECs to simulate saphenous (reversed flow, R) and muscular (non-reversed flow, N) regions. (C) Volcano plot of all microRNAs (miRNAs) on the Affymetrix ST 1.0 human microarray dataset (n = 4) from Heuslein et al.[9] HUVECs were exposed to the flow waveforms in (B). Gene expression was determined at 6 hr after simulated FAL. FDR, false discovery rate. Red dots indicate miRNAs with FDR < 0.05. (D–F) Bar graphs of mature miR-199a-5p (D), miR-146a-5p (E), and miR-29a (F) expression in HUVECs exposed to biomimetic shear stress conditions from (B) (n = 3–5). Student's t test. Data are mean ± SEM.

non-coding RNAs that are endogenous regulators of gene expression.[14] Mature miRNA incorporates into the RNA-induced silencing complex (RISC) and then binds to a target mRNA, usually in the 3′ UTR of the mRNA.[15] miRNA binding acts to suppress target gene expression by inhibiting mRNA translation to protein or by promoting mRNA degradation,[16] depending on miRNA-target complementarity.[17] Additionally, miRNAs are attractive as potential therapeutic targets as they are short, highly conserved, and can negatively regulate gene expression of multiple mRNA targets.[18] Several miRNAs have been implicated as regulators of vascular growth, primarily angiogenesis in ischemic tissue, in response to arterial occlusion.[19–27] However, the explicit role of mechanosensitive miRNAs in collateral arteriogenesis is poorly understood. Here, using a genome-wide approach on cultured ECs exposed to flow waveforms biomimetic of collateral segments exhibiting moderate and amplified arteriogenesis *in vivo*, we first identified mechanosensitive miRNA-199a as a potential regulator of collateral arteriogenesis. We then tested the hypothesis that mechanosensitive miRNA-199a regulates perfusion recovery and collateral arteriogenesis following FAL via the control of monocyte recruitment and pro-arteriogenic gene expression.

RESULTS

EC miRNA Expression Is Regulated by Shear Stress Waveforms Biomimetic of Collateral Artery Segments Exhibiting Differential Arteriogenic Responses

To identify candidate miRNAs, we examined previously generated genome-wide cDNA microarray data (GEO: GSE46248)[10] from ECs exposed to shear stress waveforms biomimetic of those measured *in vivo*[11] in collateral artery segments exhibiting either moderate (muscular) or amplified (saphenous) arteriogenesis responses (Figure 1A). Briefly, human umbilical vein ECs (HUVECs) were preconditioned for 24 hr at a baseline arterial shear stress (15 dynes/cm^2)[28] to establish EC alignment, planar cell polarity, and steady-state signaling, thereby mimicking the *in vivo* baseline state. A FAL was then simulated by a stepwise 100% increase in shear stress, in either the same direction or in the opposite direction, to mimic shear stress changes occurring in the muscular branch (non-reversed flow) and saphenous artery (reversed flow) entrance regions, respectively (Figures 1A and 1B). At 6 hr after our simulated FAL, we isolated total RNA and performed genome-wide analysis using Affymetrix cDNA 1.0 ST microarrays. Genome-wide analysis identified a small subset of miRNA genes differentially regulated between reversed and non-reversed flow conditions

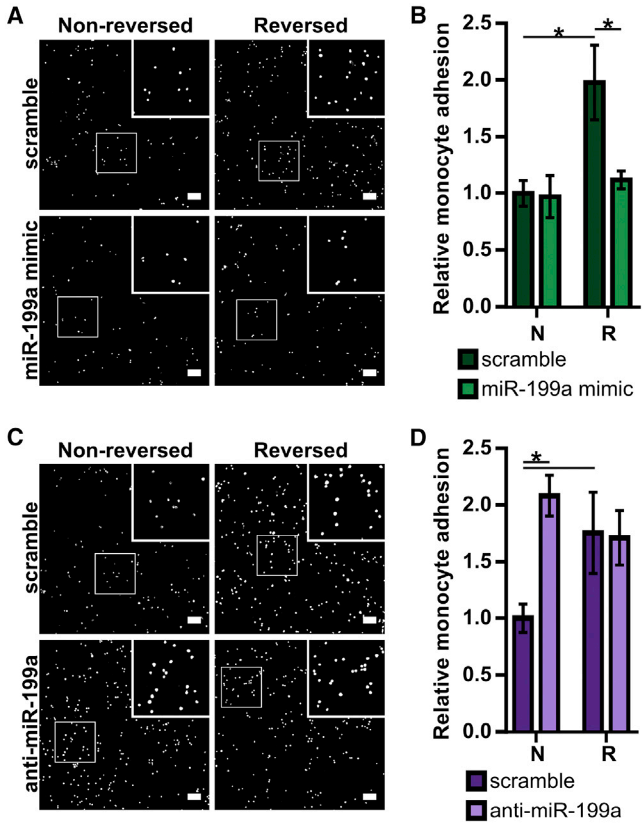

Figure 2. Monocyte Adhesion to HUVECs Exposed to Shear Stress Waveforms Biomimetic of Arteriogenic Collaterals Is Modulated by miR-199a

(A) Representative confocal microscopy images of fluorescently labeled THP-1 monocytes adhered to HUVECs transfected with miR-199a or scrambled mimic, then subjected to non-reversed (N) or reversed (R) flow waveforms outlined in Figure 1B, 6 hr after simulated FAL (scale bar, 100 μm). Insets are the magnified 300 × 300-μm regions outlined by white boxes. (B) Bar graph quantifying relative number of adhered monocytes in each condition (n = 4). *$p < 0.05$, two-way ANOVA followed by a Holm-Sidak multiple comparisons test. (C) Representative images of fluorescently labeled THP-1 monocytes adhered to flow-exposed HUVECs transfected with anti-miR-199a or scrambled locked nucleic acid oligonucleotides, 6 hr after simulated FAL (scale bar, 100 μm). (D) Bar graph quantifying relative number of adhered monocytes in each condition (n = 4). *$p < 0.05$, two-way ANOVA followed by a Holm-Sidak multiple comparisons test. Data are mean ± SEM.

(Figure 1C; Table S1). The expression of several candidate miRNAs (miR-199a-5p, miR-146a-5p, and miR-29a) was assessed by qRT-PCR both at 1 and 6 hr after simulated FAL to confirm microarray results. We found all these candidate miRNAs to be significantly down-regulated (~40%) in HUVECs exposed to the reversed flow waveform 6 hr after simulated FAL (Figures 1D–1F).

miRNA-199a Negatively Regulates Pro-arteriogenic Endothelial Gene Expression and Monocyte Adhesion to Flow-Exposed ECs *In Vitro*

Of these candidate miRNAs, miR-199a-5p (henceforth denoted as miR-199a) was of particular interest as it is known to regulate mRNAs of several pathways necessary for arteriogenesis, including inhibitor of nuclear factor kappa-B kinases subunit beta (IKKβ)[29] and caveolin-1 (Cav1).[30,31] We therefore sought to determine if miR-199a regulates the expression of these pro-arteriogenic genes in ECs exposed to the biomimetic shear stress waveforms. HUVECs were transfected with 20 nM miR-199a mimic, anti-miR-199a locked nucleic acid oligonucleotides, or respective scramble controls, 24 hr prior to shear stress exposure. At 6 hr after simulated FAL, in scramble-transfected cells, the application of a reversed flow waveform resulted in an ~50% reduction in miR-199a expression compared to non-reversed flow conditions (Figure S2), similar to previous results seen in Figure 1D. Transfection with miR-199a mimics increased relative miR-199a expression >60-fold (Figure S1A), while anti-miR-199a reduced miR-199a expression by ~80% (Figure S1B), compared to their respective scramble controls in HUVECs exposed to the biomimetic waveforms. Overexpression of miR-199a significantly decreased the relative mRNA expression of IKKβ in HUVECs exposed to reversed flow conditions, while there was only a trend toward decreased IKKβ at the protein level (Figure S2). Inhibition of miR-199a did, however, lead to a significant increase in IKKβ expression at both the mRNA and protein levels under reversed flow conditions (Figure S2). Interestingly, Cav1 expression was significantly altered only at the protein level with miR-199a modulation, suggesting miR-199a targets Cav1 by inhibiting translation in this context. Cav1 protein expression was decreased with miR-199a overexpression, whereas it was increased with miR-199a inhibition in HUVECs exposed to a reversed flow (pro-arteriogenic) waveform (Figure S3).

Monocyte adhesion to activated endothelium is a required step in the arteriogenesis cascade.[32–37] We sought to determine whether altered miR-199a expression modulates this critical process. In control conditions, HUVECs exposed to the reversed flow waveform experienced increased monocyte adhesion compared to those exposed to the non-reversed waveform (Figure 2), consistent with previous results.[10] Overexpression of miR-199a attenuated this enhanced functional response, leading to a 44% decrease in monocyte adhesion in reversed flow conditions (Figures 2A and 2B). In non-reversed flow conditions, miR-199a overexpression did not further reduce monocyte adhesion. Conversely, the inhibition of miR-199a led to enhanced pro-arteriogenic function *in vitro*, as seen by a 90% increase in monocyte adhesion to non-reversed flow-exposed HUVECs, while there was no further increase with reversed flow (Figures 2C and 2D).

Overexpression of miR-199a Limits Foot Reperfusion following FAL, while miR-199a Inhibition Elicits Complete Perfusion Recovery in BALB/c Mice

To test the hypothesis that miR-199a regulates perfusion recovery and/or arteriogenesis *in vivo*, we performed FALs on BALB/c mice, and we modulated miR-199a expression via intramuscular injection of miR-199a mimic, anti-miR-199a locked nucleic acid oligonucleotides, or respective scramble controls directly into the gracilis muscle immediately following FAL. Local intramuscular injection of miR-199a mimic led to an ~5-fold increase in relative miR-199a expression (Figure 3A), while anti-miR-199a decreased miR-199a expression ~2.6-fold in the gracilis muscle 7 days post-FAL

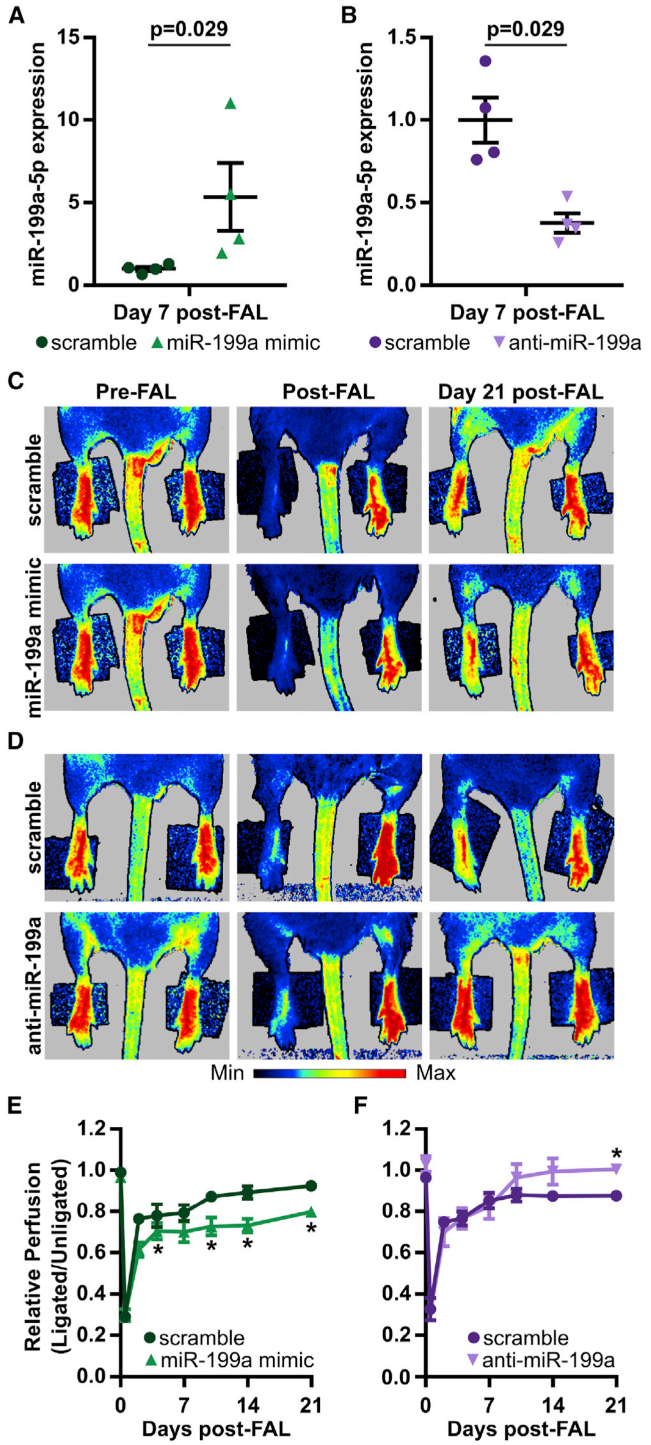

Figure 3. Overexpression of miR-199a Limits Foot Reperfusion following FAL, while miR-199a Inhibition Leads to Complete Perfusion Recovery in BALB/c Mice

(A and B) Relative miR-199a-5p expression in the gracilis muscle 7 days post-FAL in BALB/c mice. Mice were treated with a single intramuscular (i.m.) injection of 7.5 nmol miR-199a mimic (A), anti-miR-199a (B), or respective scramble oligonucleotides immediately post-FAL (n = 4). Mann-Whitney U test. (C and D) Post-FAL foot perfusion recovery as assessed by laser Doppler perfusion imaging (n = 6) for miR-199a mimic (C) and anti-miR-199a (D) treated mice. (E and F) Line graphs of perfusion recovery in miR-199a mimic (E) and anti-miR-199a (F) treated mice. *$p < 0.05$ versus scramble, two-way ANOVA with repeated measures followed by Holm-Sidak test for multiple comparisons. Data are mean ± SEM.

(Figure 3B). Additionally, we found that miR-199a overexpression decreased Cav1 protein expression and anti-miR-199a increased Cav1 protein expression compared to controls (Figure S4). Perfusion measurements of the plantar surface of the foot indicated moderate ischemia immediately post-FAL, followed by an incomplete perfusion recovery in the scramble-treated mice for both the overexpression (0.92 ± 0.02, day 21 post-FAL) and inhibition (0.88 ± 0.02, day 21 post-FAL) studies (Figures 3C–3F). When miR-199a was overexpressed, the reperfusion response was further dampened as early as 4 days post-FAL and only reached 80% reperfusion 21 days post-FAL (Figures 3C and 3E). However, anti-miR-199a-treated mice fully recovered foot perfusion by day 21 post-FAL (Figures 3D and 3F).

Overexpression of miR-199a Inhibits Arteriogenesis in BALB/c Mice

We next examined whether overexpression of miR-199a affects arteriogenesis by measuring the lumenal diameter of gracilis collateral arteries 21 days post-FAL (Figures 4A and 5A). While both scramble- and miR-199a mimic-treated mice experienced significant ($p < 0.0001$) arteriogenesis in their ligated limbs compared to sham-operated controls, treatment with an miR-199a mimic significantly ($p = 0.0008$) reduced collateral artery growth by >25% (Figure 4B). Moreover, this reduction in arteriogenesis occurred at both the muscular (non-reversed) and saphenous (reversed) collateral artery regions (Figure S5). Cross-sectional analysis of these collateral arteries was used to confirm whole-mount diameter measurements and to determine collateral wall area (Figure 4C). Mice treated with miR-199a mimic experienced a lesser degree of enhancement in lumenal diameter (Figure 4D) and wall area (Figure 4E) than scramble controls, while there was no significant difference ($p = 0.087$) in the diameter-to-wall area ratio 21 days after FAL.

Inhibition of miR-199a Amplifies Arteriogenesis in BALB/c Mice following FAL

In contrast, when we administered a single intramuscular injection of an anti-miR-199a locked nucleic acid oligonucleotide to mice immediately after FAL, arteriogenesis was significantly amplified (Figure 5A). Inhibition of miR-199a generated a 36% increase in collateral lumenal diameter 21 days post-FAL ($p < 0.0001$) (Figure 5B). Again, cross-sectional analysis was used to confirm whole-mount diameter measurements and to determine wall area (Figure 5C). Following FAL, intramuscular anti-miR-199a treatment produced increased collateral artery lumenal diameter (Figure 5D) and wall area (Figure 5E) compared to scramble-treated BALB/c mice, while there was no significant difference in the diameter-to-wall area ratio (Figure 5F). The enhancement in lumenal diameter and wall area in response to miR-199a inhibition was evident along the collateral at both the muscular (non-reversed) and saphenous (reversed) regions (Figure S6).

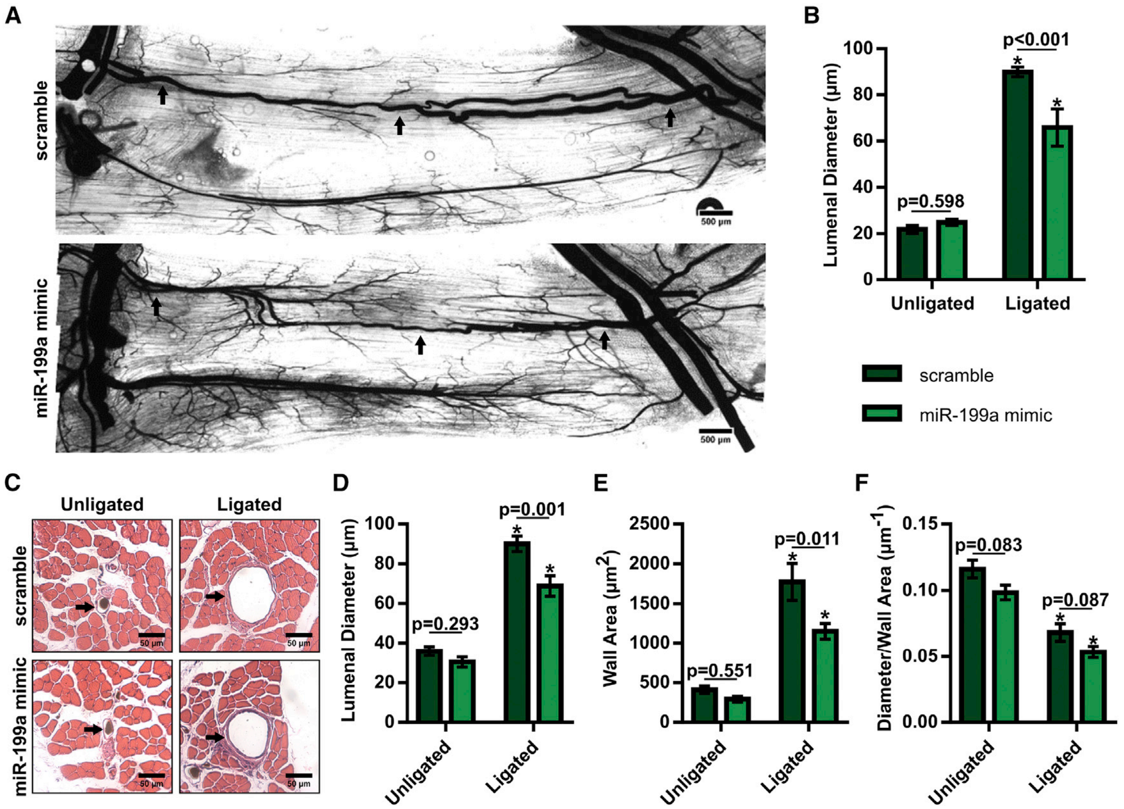

Figure 4. Overexpression of miR-199a Inhibits Arteriogenesis in BALB/c Mice

(A) Representative whole-mount vascular cast images of gracilis collateral arteries 21 days post-FAL in scramble- (top) or miR-199a mimic- (bottom) treated BALB/c mice. (B) Bar graph of mean lumenal diameter along collateral artery length for ligated and unligated limbs of miR-199a mimic- and scramble-treated mice (n = 5–6 for mimic and scramble groups, respectively). *p < 0.001 versus unligated, two-way ANOVA followed by Holm-Sidak test for multiple comparisons. (C) Representative H&E-stained cross sections of collateral arteries from ligated and unligated limbs. (D–F) Bar graphs of lumenal diameter (D), wall area (E), and diameter per wall area (F) from H&E-stained cross sections (n = 5–6 for mimic and scramble groups, respectively). *p < 0.001 versus unligated, two-way ANOVA followed by Holm-Sidak test for multiple comparisons. Arrows indicate the primary collateral artery in (A) and (C). Data are mean ± SEM.

Pericollateral Macrophage Recruitment Is Modulated by miR-199a

We next sought to determine whether modulating miR-199a expression altered macrophage recruitment, a necessary component of collateral artery growth,[32–38] *in vivo*. miR-199a overexpression caused a 36% decrease in pericollateral Mac3$^+$ macrophages 7 days post-FAL in BALB/c mice, whereas miR-199a inhibition caused a 43% increase in pericollateral macrophage recruitment (Figure 6). This trend was also observed when the muscular (non-reversed) and saphenous (reversed) collateral artery regions were assessed individually (Figure S7). We observed a similar trend across all groups when using F4/80$^+$ as an additional macrophage-specific marker[39] (Figure S8).

miRNA-199a Inhibition Improves Gastrocnemius Muscle Composition in FAL-Operated BALB/c Mice

Next, we examined the effect of altered miR-199a expression on the composition of ischemic muscle tissue downstream of the femoral artery occlusion. Gastrocnemius muscle tissue was categorized as viable (mature and regenerating fibers) or non-viable (necrotic and fibro-adipose tissue) by histological analysis 21 days after FAL (Figures 7A and 7B). Though muscle composition was not significantly altered in miR-199a mimic-treated mice (Figure S9), inhibition of miR-199a resulted in a greater than 34% reduction in the percentage of non-viable tissue in the gastrocnemius muscle (Figure 7E). Masson trichrome staining was used as an additional metric to assess fibrosis in the gastrocnemius muscle 21 days post-FAL.[40,41] Similar to H&E staining, anti-miR-199a-treated mice exhibited a trend toward decreased fibrotic area compared to scramble controls (Figures 7C, 7D, and 7F). There was no difference in fibrosis between miR-199a mimic- and scramble control-treated mice (Figure S9).

Plasma miRNA-199a Expression Is Elevated in Patients with PAD

Finally, we sought to determine if miR-199a expression is altered in human PAD patients. A diagnosis of PAD with intermittent claudication was based on having one of the following: (1) an ankle-brachial index (ABI) < 0.9, (2) a previous peripheral vascular intervention, or (3) an abnormal tibial-brachial index (TBI), without any corresponding foot ulcers or resting pain. Plasma was collected from

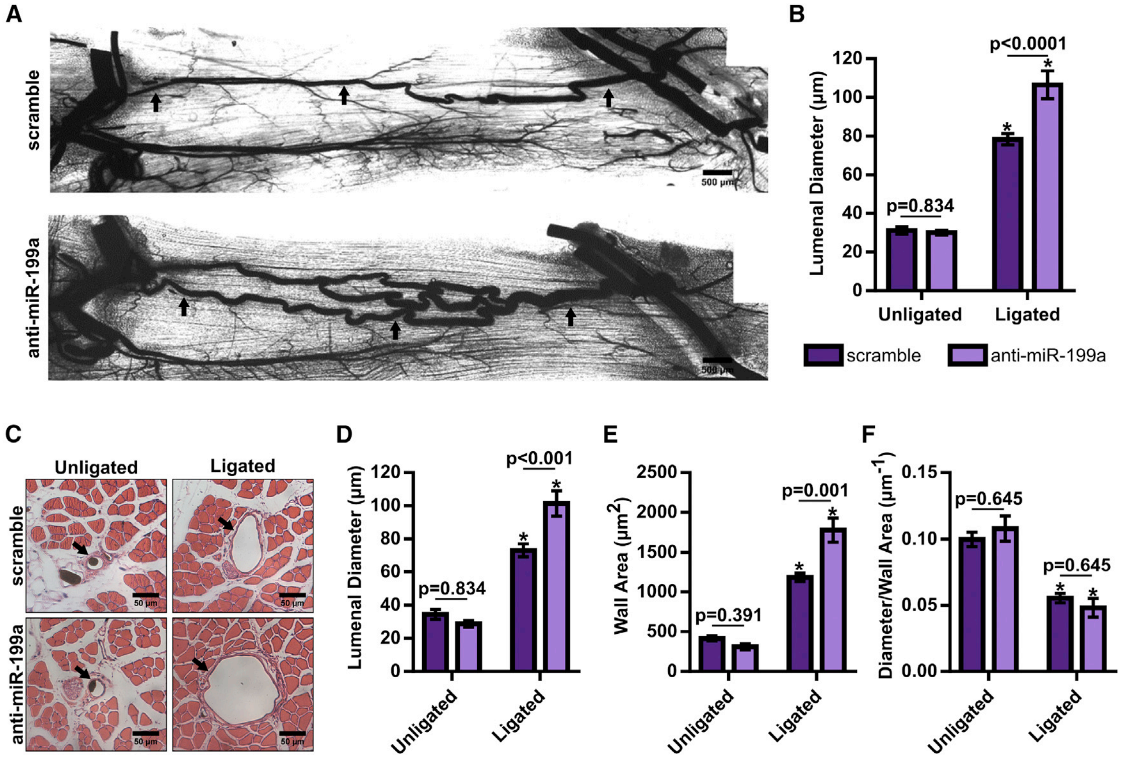

Figure 5. Inhibition of miR-199a Amplifies Arteriogenesis in BALB/c Mice following FAL

(A) Representative whole-mount vascular cast images of gracilis collateral arteries 21 days post-FAL in non-targeting scramble- (top) or anti-miR-199a- (bottom) treated BALB/c mice. (B) Bar graph of mean lumenal diameter along collateral artery length for ligated and unligated limbs of anti-miR-199a- and scramble-treated mice (n = 6). *p < 0.001 versus unligated, two-way ANOVA followed by Holm-Sidak test for multiple comparisons. (C) Representative H&E-stained cross sections of collateral arteries from ligated and unligated limbs. (D–F) Bar graphs of lumenal diameter (D), wall area (E), and diameter per wall area (F) from H&E-stained cross sections (n = 6). *p < 0.001 versus unligated, two-way ANOVA followed by Holm-Sidak test for multiple comparisons. Arrows indicate the primary collateral artery in (A) and (C). Data are mean ± SEM.

PAD patients with intermittent claudication (minimum ABI = 0.75 ± 0.26) and risk factor-controlled patients (minimum ABI = 1.09 ± 0.12) who otherwise exhibited similar PAD risk factors, such as age, sex, diabetes, hypertension, and hyperlipidemia (Table S2). We found relative plasma miR-199a expression to be significantly elevated (p = 0.033) in PAD patients relative to risk factor control patients (Figure 8).

DISCUSSION

The primary goal of this study was to determine whether mechanosensitive miRNAs regulate endogenous collateral artery growth and perfusion recovery following femoral arterial occlusion. First, we identified candidate regulators of arteriogenesis by comparing differential miRNA expression in ECs exposed to shear stress waveforms corresponding to moderate and amplified arteriogenesis responses *in vivo*. Among these candidate miRNAs, miR-199a was significantly downregulated by the amplified arteriogenesis (reversed flow) waveform and shown to regulate pro-arteriogenic molecule (IKKβ and Cav1) expression and monocyte adhesion to shear stress-exposed endothelium. *In vivo*, the overexpression of miR-199a limited foot perfusion, arteriogenesis, and monocyte recruitment in the mouse FAL model. In contrast, the inhibition of miR-199a elicited complete foot perfusion recovery, substantially enhanced collateral arteriogenesis via increased pericollateral macrophage recruitment, and considerably improved gastrocnemius muscle tissue composition. Finally, we showed miR-199a expression is elevated in the plasma of PAD patients with intermittent claudication compared to a risk factor control population, further indicating its relevance in human disease. We have, therefore, identified miR-199a as a novel mechanosensitive miRNA that regulates perfusion recovery and arteriogenesis. miRNA-199a inhibition may represent a new target for the therapeutic stimulation of arteriogenesis and the treatment of PAD.

Identification of miRNA-199a as a Novel Mechanosensitive Regulator of Pro-arteriogenic Gene Expression

Our strategy of interrogating differential mechano-signaling in ECs exposed to a biomimetic amplified arteriogenesis waveform represents a unique, transformative approach for identifying miRNA regulators of vascular growth and perfusion recovery in response to arterial occlusion(s). This strategy was enabled by our group's previous development of a transillumination laser speckle flowmetry method customized for measuring blood velocities in the collateral arteries of the mouse gracilis adductor muscle.[11] Using this method, we were able to determine segment-to-segment hemodynamic

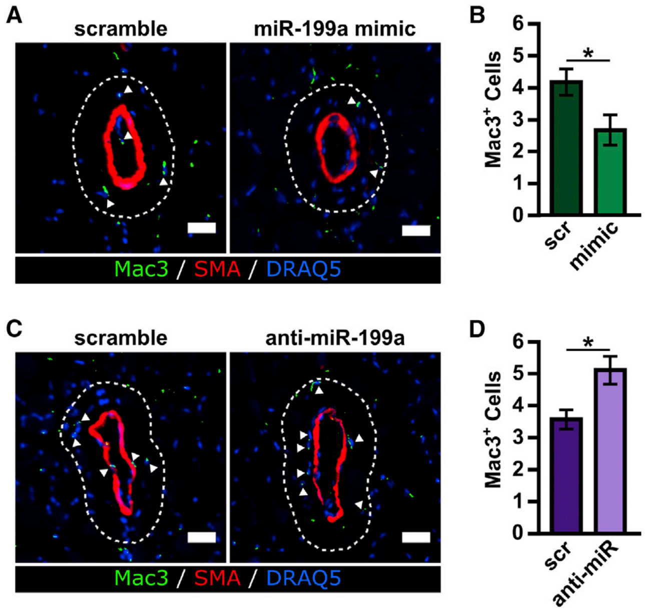

Figure 6. Pericollateral Macrophage Recruitment Is Modulated by miR-199a

(A) Representative cross sections of gracilis collateral artery regions immunolabeled for macrophage marker, Mac3 (green), smooth muscle alpha actin (SMαA, red), and nuclei (DRAQ5, blue) in BALB/c mice treated with miR-199a mimic or scramble mimic 7 days post-FAL. Dotted line indicates the pericollateral region (25 μm from vessel wall) used for quantification. Arrowheads indicate Mac3$^+$ cells (scale bar, 25 μm). (B) Bar graph of pericollateral Mac3$^+$ cells (n = 4–5 for miR-199a mimic and scramble, respectively). *$p < 0.05$, Student's t test. (C) Immunolabeled gracilis collateral artery regions, as in (A), in BALB/c mice treated with anti-miR-199a or scramble oligonucleotide 7 days post-FAL. (D) Bar graph of pericollateral Mac3$^+$ cells (n = 3). *$p < 0.05$, Student's t test. Data are mean ± SEM.

changes induced by FAL,[11] correlate these to variations in arteriogenesis,[10] and directly apply these flow waveforms to ECs *in vitro*. The utility of this approach is supported by our present results, as well as by our approach's identification of miR-100 as another candidate miRNA (Figure 1C). Inhibition of miR-100 has been previously shown to enhance perfusion recovery following FAL.[20] When considered in light of our findings, we postulate that the enhancement of shear stress-mediated collateral arteriogenesis may be a probable component of perfusion recovery with anti-miR-100 treatment.

We also identified miR-34a and miR-146a as potential mechanosensitive regulators of arteriogenesis that could be targeted for therapeutic arteriogenesis (Figure 1). Indeed, we have recently shown that miR-146a-5p inhibition improves collateral artery growth following FAL.[42] However, based on their known downstream targets and functions, we postulate that these miRNAs could also be subject to the so-called Janus phenomenon, wherein pro-arteriogenic therapies also tend to promote atherosclerosis.[43] To this end, both miR-34a and miR-146a have been found to be significantly upregulated in human atherosclerotic plaques.[44] Moreover, deletion of miR-146a in the vasculature increases EC activation and atherogenesis, though miR-146a deletion in bone marrow-derived cells reduces atherogenesis despite paradoxically elevating circulating pro-inflammatory cytokines in Ldlr$^{-/-}$ mice on a high-cholesterol diet.[45]

To date, miR-199a has been mostly studied for its roles in cardiac hypertrophy,[46,47] smooth muscle proliferation,[48] angiogenesis,[49–54] and as a tumor suppressor in a variety of cancers.[29,50,52,55–59] However, miR-199a also regulates several genes important for arteriogenesis via direct seed sequence binding to target mRNAs. To this end, it has been shown that miR-199a negatively regulates nuclear factor κB (NF-κB), which is necessary for arteriogenesis,[60] by directly targeting IKKβ.[29] Additionally, Cav1, a critical component of caveolae shown to be key in mechanotransduction,[61] the response to arterial occlusion,[30,31] and neovascularization,[62] has been previously shown to be a direct target of miR-199a.[63–67] Here we found that miR-199a alters both IKKβ and Cav1 expression in ECs exposed to our pro-arteriogenic biomimetic shear stress waveforms. Moreover, Cav1 protein expression was regulated by miR-199a expression in the gracilis muscle *in vivo*, suggesting a potential pathway by which altered miR-199a is able to regulate arteriogenesis. Furthermore, through both loss- and gain-of-function studies, we found miR-199a to regulate monocyte adhesion to ECs, a key aspect of arteriogenesis. Altogether, this made miR-199a a compelling candidate miRNA to study in the context of arteriogenesis *in vivo*.

Previous Studies Implicating a Role for miRNAs in Regulating Arteriogenesis

Despite a number of previous reports indicating miRNA regulation of perfusion recovery in hindlimb ischemia models,[19–27,68] only a few of these miRNAs (e.g., miR-155, miR-17∼92a, miR-93, miR-487b, and miR-352) have been suggested to have a role in arteriogenesis per se. Inhibition of miR-155 attenuates blood flow recovery and leukocyte recruitment, despite promoting angiogenesis in the ischemic tissue of FAL-treated mice, thereby indirectly implying a role for miR-155 in arteriogenesis. However, collateral diameters were not explicitly reported.[26] In another study, endothelial-specific deletion of the miR-17∼92 cluster increased foot perfusion 14 days following FAL as well as arteriole density in FAL-operated limbs.[27] More recently, overexpression of miR-93 was sufficient to rescue the impaired perfusion recovery, capillary density, and arteriolar density observed in miR-106b-93-25$^{-/-}$ mice following femoral artery excision via an miR-93-IRF9-IRG1-itaconic acid pathway leading to increased M2-like macrophage polarization in ischemic tissue.[68] Additionally, miR-329, miR-487b, miR-494, and miR-495 inhibition all led to a 25%–40% increase in blood flow recovery following FAL, though only miR-487b inhibition increased vessel diameters from PBS-treated controls.[24] While these studies further corroborate miRNA-mediated regulation of arteriogenesis, it is difficult to directly compare our results due to differences in oligonucleotide dosing, the extent of ischemia in the various animal models, and methods for assessing arteriogenesis.

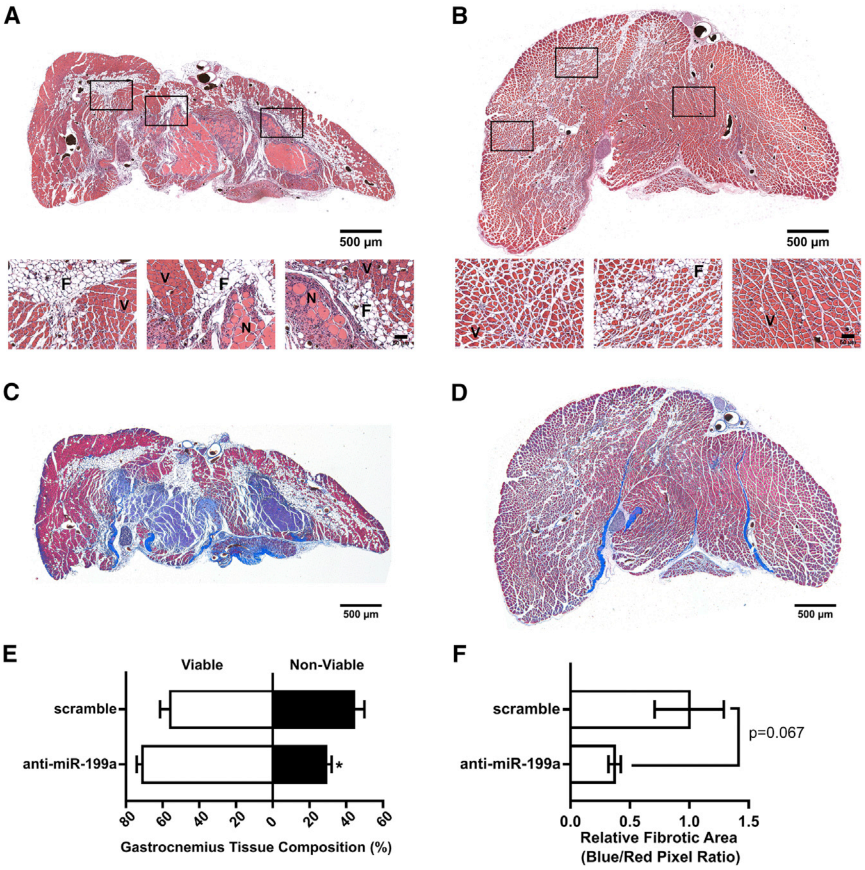

Figure 7. miRNA-199a Inhibition Improves Gastrocnemius Muscle Composition in FAL-Operated BALB/c Mice

(A and B) Representative images of H&E staining of gastrocnemius muscle for ligated limb of BALB/c mice treated with scramble (A) or anti-miR-199a (B) locked nucleic acid oligonucleotide immediately after FAL (scale bar, 500 μm; inset scale bar, 50 μm). V, viable muscle; N, necrotic tissue; F, fibro-adipose tissue. (C and D) Representative images of Masson trichrome-stained gastrocnemius muscles from ligated limb of BALB/c mice treated with scramble (C) or anti-miR-199a (D) (scale bar, 500 μm). Blue staining is indicative of collagen and/or fibrotic content whereas red staining indicates healthy tissue. (E) Bar graph of the percentage of gastrocnemius muscle that is viable (white) or non-viable (black) at day 21 post-FAL in each group (n = 5). (F) Bar graph of the relative fibrotic area in gastrocnemius muscle at day 21 post-FAL in each group (n = 5). $^{*}p < 0.05$ versus scramble, Student's t test. Data are mean ± SEM.

To our knowledge, only one other previous study has examined flow-responsive miRNAs in the regulation of arteriogenesis. In a rat arteriovenous (AV) shunt model of amplified arteriogenesis due to chronically elevated blood flow, 94 miRNAs were found to be differentially expressed.[69] Of these, miR-353 was found to be downregulated in the pro-growth, chronically elevated blood flow condition. Subsequent miR-352 inhibition increased the number of collateral arteries, proliferation within the vessel wall, and collateral flow,[69] though the direct regulation of endothelial miR-352 expression by shear stress was not shown. Interestingly, miR-199a-5p was also downregulated (−2.56-fold in AV shunt [i.e., pro-growth] versus control) in this alternative model of amplified arteriogenesis, providing further support that the inhibition of miR-199a promotes arteriogenesis.

miRNA-199a as a Regulator of Arteriogenesis Leading to Enhanced Perfusion Recovery

In testing our hypothesis that miR-199a negatively regulates perfusion recovery and arteriogenesis *in vivo*, we chose to perform our FAL model in BALB/c mice to enable the necessary dynamic range for both loss- and gain-of-function studies, as these mice exhibit a more blunted arteriogenic response compared to C57BL/6 mice.[70,71] Following FAL, scramble-treated mice exhibited incomplete perfusion recovery as expected, though they did recover 80%–90% perfusion in ligated limbs compared to the ~50%–60% perfusion recovery seen in previous studies.[71] This greater baseline recovery response was likely due to the more mild nature of our ligation scheme; nonetheless, we were still able to observe significant changes in foot perfusion with the modulation of miR-199a expression, similar to those seen by previous miRNA studies.[20,23,72] We also emphasize that our FAL model is still severe enough to elicit a substantial transformation of skeletal muscle into necrotic and fibro-adipose tissue in the calf, which is characteristic of PAD in humans.

Corresponding to the observed changes in foot perfusion, we found that miR-199a negatively regulates arteriogenesis. Our results indicating miR-199a modulates monocyte and/or macrophage recruitment are consistent with the known necessary role for leukocyte recruitment in arteriogenesis,[32,35,36,73] and they suggest that this is the mechanism through which miR-199a regulates arteriogenesis following FAL. Critical in determining the reperfusion response, alterations to endogenous collateral growth can lead to substantial changes in collateral conductance. To this end, miR-199a overexpression attenuated collateral artery growth ~25% (~2.4-fold decrease in conductance) and miR-199a inhibition enhanced arteriogenesis by 36% (>3.4-fold conductance increase). Of particular importance for potential clinical translation, collateral arteries in anti-miR-199a-treated mice were morphologically normal, as there was no difference in the lumenal diameter-to-wall area ratio when miR-199a expression was altered (Figure 6F).

In addition to arteriogenesis, perfusion recovery following FAL can also be dependent upon angiogenesis[7] and/or tissue clearance and skeletal muscle regeneration.[8] Here we did not observe any significant

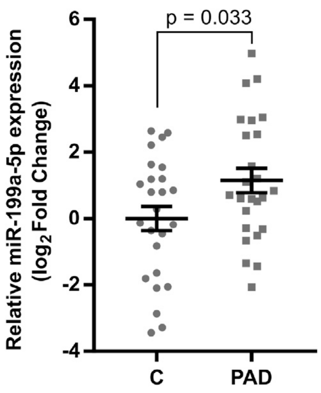

Figure 8. miR-199a-5p Expression Is Elevated in the Plasma of PAD Patients with Intermittent Claudication

Log_2 fold change of miR-199a-5p expression in plasma from human PAD patients with intermittent claudication (PAD) versus a risk factor control population (C). Plasma miR-199a-5p expression was normalized to RNU6 (n = 25). *p < 0.05, Student's t test. Data are mean ± SEM.

differences in gastrocnemius capillary density within viable and non-viable tissue regions in anti-miR-199a-treated mice at day 21 post-FAL (Figure S10). However, we did find that a single bolus of anti-miR-199a oligonucleotide was sufficient to improve gastrocnemius muscle tissue composition in FAL-operated mice (Figure 8), though there was no significant change in muscle composition with miR-199a overexpression (Figure S9). While it is probable that this improvement in muscle composition is a consequence of enhanced downstream tissue perfusion due to amplified arteriogenesis with miR-199a inhibition, miR-199a could also play a direct role in tissue clearance and regeneration. Indeed, miR-199a has been previously implicated in tissue fibrosis,[66] myoblast proliferation and differentiation,[74] and adipocyte differentiation,[75] all of which are consistent with the hypothesis that miR-199a can directly regulate skeletal muscle proliferation and/or differentiation in a hypoxic environment. However, in our hands, miR-199a inhibition did not alter cell survival of C2C12 myoblasts after exposure to hypoxia and serum starvation conditions (Figure S11). Though future work could test the cell-specific contribution of miR-199a in response to FAL, our present results indicate that improved muscle composition with anti-miR-199a treatment is secondary to enhanced upstream arteriogenesis.

Clinical Perspective

Based on both our results and previous studies, we postulate that miRNA-199a inhibition represents a potentially promising treatment for arterial occlusive diseases. Most patients with PAD also have concomitant coronary artery disease (CAD).[76] Previously, miR-199a-5p was shown to be upregulated in patients with CAD versus matched controls,[77] though its expression in PAD was unknown. Here we observed that plasma miR-199-5p expression is elevated in PAD patients with intermittent claudication versus a risk factor control population. Moreover, our *in vivo* results indicate that miR-199a inhibition improves limb perfusion by enhancing arteriogenesis and reduces the fibro-adipose composition of ischemic muscle, which is often found in PAD patients.[78–81] Together, these data indicate anti-miR-199a-5p delivery strategies may have a therapeutic benefit for PAD patients.

MATERIALS AND METHODS

HUVEC Culture

HUVECs purchased from VEC Technologies (Rensselaer, NY) were thawed and maintained on 0.1% gelatin-coated flasks in M-199 medium (Lonza, Basel, Switzerland), supplemented with 10% fetal bovine serum (Life Technologies, Grand Island, NY), 100 U/mL penicillin-G + 100 μg/mL streptomycin (Life Technologies), 2 mmol/L L-glutamine (Life Technologies), 5 μg/mL EC growth supplement (Biomedical Technologies, Stoughton, MA), and 10 μg/mL heparin (Sigma-Aldrich, St. Louis, MO). For each set of experimental comparisons, cells were used from the same cell line between subculture passages 2 and 3.

In Vitro Exposure of ECs to Biomimetic Shear Stress Waveforms

HUVECs were plated on cell culture grade plastic dishes coated with 0.1% gelatin and grown to confluence. A cone and plate flow apparatus,[82] which maintains cells at 5% CO_2 and 37°C, was used to induce a shear stress protocol. The applied shear stress protocol consisted of a 24-hr preconditioning period at a steady 15 dyne/cm^2, which was then either increased to 30 dynes/cm^2 (non-reversed flow) or increased to 30 dynes/cm^2 and reversed in direction (reversed flow) to simulate relative hemodynamics previously quantified in our *in vivo* FAL model.[11] Fresh culture medium, consisting of M199 with 4% dextran from *Leuconostoc* spp (Sigma-Aldrich, M_r ∼500,000), 2% fetal bovine serum, 100 U/mL penicillin-G + 100 μg/mL streptomycin, 2 mmol/L L-glutamine, 5 μg/mL EC growth supplement, and 10 μg/mL heparin, was added to cells before exposure to shear stress, and it was continuously exchanged throughout the duration in the cone and plate apparatus.

HUVEC Microarray Gene Expression Profiling

Microarray analysis was performed as previously described and is publicly available at GEO: GSE46248 (https://www.ncbi.nlm.nih.gov/geo/). Volcano plots were generated using the expression change and false discovery rates (FDRs) from the reversed versus non-reversed (RvN) and reversed versus control (RvC) datasets, respectively.

Transfection of miRNA Antagomirs and Mimics of HUVECs

At 24 hr prior to the exposure of HUVECs to flow conditions, HUVECs were plated without antibiotics on 0.1% gelatin-coated plates in serum-free M199 (Life Technologies) supplemented with 10% fetal bovine serum, 2 mmol/L L-glutamine, 5 μg/mL EC growth supplement (Biomedical Technologies, Stoughton, MA), and 10 μg/mL heparin (Sigma-Aldrich). After cells were allowed to adhere for 2 hr after plating, cells were transfected using Lipofectamine RNAiMax (Invitrogen, Carlsbad, CA), according to the manufacturer's instructions. For inhibitor experiments, HUVECs were transfected with 20 nM scramble or miR-199a-5p locked nucleic acid (LNA) inhibitors (Power Inhibitors, Exiqon, Vedbaek, Denmark).

For overexpression experiments, HUVECs were transfected with 20 nM scramble or miR-199a-5p LNA mimic (miRCURY LNA mimics, Exiqon).

HUVEC RNA Isolation and qRT-PCR

Total RNA was extracted using the PureLink total RNA purification system (Life Technologies) using the on-column DNase protocol (Life Technologies), according to the manufacturer's instructions. RNA concentration and purity were determined with a NanoDrop spectrophotometer in duplicate.

For qRT-PCR, 500 ng total RNA was reverse transcribed using the miScript II reverse transcription kit (QIAGEN, Hilden, Germany, 218160) with HiFlex buffer mix, according to the manufacturer's instructions. Following reverse transcription, RT-PCR was performed on 20 ng cDNA using miScript SYBR Green PCR kit (QIAGEN, 218073) and miScript Primer Assays (QIAGEN) for U6 (MS00033740), miR-199a-5p (MS00006741), miR-146a-5p (MS00003535), and miR-29a (MS00003262) on a CFX96 Real Time Detection System (Bio-Rad). Normalized expression to U6 was quantified using the comparative $2^{\Delta\Delta Ct}$ method.

Target mRNA expression was assessed by qRT-PCR on 12.5 ng reverse transcribed cDNA with FastStart SYBR Green (Roche Applied Sciences, Penzberg, Germany) on a CFX96 Real Time Detection System (Bio-Rad, Hercules, CA). Primers used were as follows: CD44 (forward 5′-GCAGCCAACTTCCGAGGCAGC-3′, reverse 5′-CGGA GGACGGGACGAGGATGG-3′), CCND1 (forward 5′-AACTACCT GGACCGCTTC-3′, reverse 5′-GAGTTGTCGGTGTAGATGC-3′), and IKKβ (forward 5′-GCCTGGGAAATGAAAGAGCG-3′, reverse 5′-ATCTGCTCACCTGTTTCCTGA-3′). Expression was normalized to β2-microglobulin (forward 5′-AGCATTCGGGCCGAGATGT CT-3′, reverse 5′-CTGCTGGATGACGTGAGTAAACCT-3′), which is endogenously expressed and is not altered by many stimuli, including shear stress.[83]

Cav1 mRNA expression was assessed by qRT-PCR on 12.5 ng cDNA with SensiMix II (Bioline, London, UK, BIO-83005) using Cav1-FAM (Hs00971716_m1) and B2M-VIC (Hs00187842_m1) TaqMan primer probes, purchased from Thermo Fisher Scientific, on a CFX96 Real Time Detection System. Normalized expression was quantified using the comparative $2^{\Delta\Delta Ct}$ method.

Monocyte Adhesion Functional Assay

Human-derived monocytes (THP-1 cell line) were purchased from the ATCC. Monocytes were thawed and maintained in RPMI 1640 (11875-093, Life Technologies) + 10% fetal bovine serum (Life Technologies) + 0.05 mM β-mercaptoethanol per ATCC culture instructions. Monocytes sub-cultured once cell density approached 800,000 cells/mL. Cells were used between passages 2 and 6.

Prior to the adhesion assay, cells were counted to obtain 3,000,000 cells/plate of HUVECs. Cells were pelleted, washed with PBS, pelleted, and then re-suspended in serum-free RPMI medium at 1,000,000 cells/mL. Thawed calcein acetoxymethyl (AM) was added at 1 μg/mL and incubated with cells for 15 min at 37°C. After 15 min, the reaction was stopped by adding excess serum-free RPMI to the cell solution then pelleted. Cells were washed once with serum-free M199 medium, pelleted, and then re-suspended in serum-free M199 at 500,000 cells/mL. Immediately following completion of flow exposure to HUVECs, flow medium was removed by aspiration. HUVECs were quickly washed with serum-free M199 medium. This medium was then aspirated off and 6 mL serum-free M199 + monocytes (3,000,000/plate) were added to and incubated with HUVECs for 30 min at 37°C. Following the 30 min, cells were washed twice with PBS to remove unbound monocytes. Adhered monocytes and HUVECs were fixed with 4% paraformaldehyde (PFA) for 10 min followed by two washes with PBS. Coverslips were mounted with Prolong Gold (Life Technologies). Plates were then imaged using a Nikon TE2000 C1 laser-scanning confocal microscope. Nine randomly selected fields of view (FOVs) per condition for 4 independent experiments were obtained. Images were de-identified and randomized in MATLAB. Images were converted to 8-bit images, set to an equivalent threshold, and bound monocytes were quantified using Fiji's Analyze Particles tool (20 μm^2 minimum particle size). Results were centered on the mean of all conditions within each independent experiment.

Mice

All animal protocols were approved by the Institutional Animal Care and Use Committee at the University of Virginia (protocol 3814), and they conformed to all regulations for animal use outlined in the American Heart Association Guidelines for the Use of Animals in Research. BALB/c mice were purchased from Charles River Laboratory (Wilmington, MA). All animals were housed in the animal facilities at the University of Virginia.

FAL Model

We used a previously detailed FAL scheme[10,84,85] that produces consistent arteriogenesis in the collateral arteries of the gracilis adductor muscles,[7,8,10,86–89] along with minimal heterogeneity in the baseline collateral structure and with known changes in flow direction from baseline. Male mice, 10–12 weeks of age, were anesthetized (intraperitoneally [i.p.], 120 mg/kg ketamine, 12 mg/kg xylazine, and 0.08 mg/kg atropine), depilated, and prepped for aseptic surgery. On the left leg, an incision was made directly above and along the femoral artery, which was gently dissected from the femoral vein and nerve between the bifurcation of the superior epigastric artery and popliteal artery. Two 6.0 silk sutures were placed immediately distal to the epigastric artery, which served as the origin of the muscular branch artery in all mice, and the artery segment between the two ligatures was then severed with micro-dissecting scissors. The surgical site was then closed with 5.0 prolene sutures. A sham surgery, wherein the femoral artery was exposed, but not ligated, was performed on the right hindlimb (i.e., on the other leg). Animals received one injection of buprenorphine for analgesia at the time of surgery and a second dose 8–12 hr later.

In Vivo miR-199a Antagomir Treatment

Anti-miR-199a-5p (5′-TAGTCTGAACACTGGG-3′) and scramble control (5′-ACGTCTATACGCCCA-3′) LNA oligonucleotides were purchased from Exiqon. Oligonucleotides were reconstituted in sterile TE buffer and stored at 1.2 nmol/μL at −20°C. Prior to use, aliquots of oligonucleotides were thawed and diluted in sterile saline to a final concentration of 0.25 nmol/μL. Immediately following FAL, 7.5 nmol oligonucleotide was injected into each (ligated and sham-operated) gracilis muscles.

In Vivo miR-199a Mimic Treatment

In vivo ready miRVana miR-199a-5p (4464070, MC10893) mimic and scramble control (4464061) mimic were purchased from Ambion. Mimics (250 nmol) were reconstituted in sterile nuclease-free water and stored at 0.5 nmol/μL at −20°C. Prior to use, aliquots of oligonucleotides were thawed and diluted in sterile saline to a final concentration of 0.25 nmol/μL. Immediately following FAL, 7.5 nmol oligonucleotide was injected into both ligated and sham-operated gracilis muscles.

Laser Doppler Perfusion Imaging

For monitoring blood flow recovery and post-surgical ischemia, mice were anesthetized via 1.5% isoflurane under constant oxygen. Mice were placed in a prone position and the soles of the feet were scanned (PeriCam PSI, PeriMed, Stockholm, Sweden). Mean foot perfusion was used to calculate relative perfusion ratio (ratio of ligated over unligated).

Tissue Harvesting for miRNA Expression

At 7 days after FAL, mice were anesthetized (i.p., 120 mg/kg ketamine, 12 mg/kg xylazine, and 0.08 mg/kg atropine) and euthanized via an overdose of pentobarbital sodium and phenytoin sodium (Euthasol, Virbac, Fort Worth, TX). The left ventricle was cannulated with a 23G catheter (right ventricle was carefully opened to act as a sink for perfusate), and the entire body was perfused with 7 mL Tris-$CaCl_2$ (0.1 g/L $CaCl_2$) containing 2% heparin, 2 mmol/L adenosine (16404, Fisher Scientific, Pittsburgh, PA), and 0.1 mmol/L papaverine (P3510, Sigma-Aldrich, St. Louis, MO) to clear and vasodilate the downstream vasculature at a constant rate of 1.5 mL/min (PHD2000, Harvard Apparatus). Once perfused, we waited 5 min to enable vasodilation. The entire body was then perfused with 14 mL Tris-$CaCl_2$, and both gracilis muscles were dissected free, placed in RNAlater (Ambion), and stored at −20°C.

RNA Isolation from Tissues and qRT-PCR

Excess RNAlater was removed from tissues. Tissues were then incubated in 450 μL TRIzol reagent for 5 min at room temperature. Tissues were then placed on ice and homogenized using a power homogenizer (Omni International, Kennesaw, GA) in short bursts to avoid overheating. Following homogenization, an additional 550 μL TRIzol reagent was added. Samples were incubated for another 5 min at room temperature to ensure complete lysis. 200 μL chloroform was added to each sample. Samples were then shaken vigorously for 15 s and incubated for 3 min at room temperature. Following this incubation, samples were centrifuged for 10 min at 12,000 × *g* at 4°C. The resulting aqueous layer was carefully removed, placed in a new RNA and DNase-free tube, and an equal volume of 70% ethanol was added to save the aqueous layer. RNA isolation then proceeded using the PureLink total RNA purification system (Life Technologies) with the on-column DNase protocol (Life Technologies), according to the manufacturer's instructions. RNA concentration and purity were determined with a NanoDrop spectrophotometer in duplicate.

For reverse transcription (Applied Biosystems, 4366596) and miR-199a-5p detection, TaqMan miRNA assays (Applied Biosystems, 4427975) were used. Real-time qPCRs were done on an ABI Prism 7900 HT detection system (Applied Biosystems). Gene expression was normalized to RNU6 (Applied Biosystems, 4427975), and the relative expression was determined with the comparative $2^{\Delta\Delta Ct}$ method.

Protein Expression of miR-199a-5p Targets

HUVECs were directly lysed in ice-cold radioimmunoprecipitation assay (RIPA) buffer (Sigma, R0278) with protease inhibitor (Sigma, 1:100, P8340). For mouse tissues, half of the entire gracilis muscle was placed in 1 mL RIPA buffer supplemented with protease inhibitor (Sigma, 1:100, P8340) on ice, and then it was homogenized using a power homogenizer (Omni International) in short bursts to avoid overheating. Lysed samples (both HUVEC and gracilis muscle) were then cleared for 30 min at 4°C under constant agitation. Samples were centrifuged for 1 min at 10,000 × *g*, the supernatant was collected, and a Pierce bicinchoninic acid (BCA) assay (Thermo Fisher Scientific, 23225) was used to determine total protein concentration. Samples were diluted 1:1 in 4× XT sample buffer (Bio-Rad, 161-0791) with XT reducing reagent (Bio-Rad, 161-0792, 1× final concentration) and boiled for 10 min. Equal protein was loaded onto a 4%–12% Bis-Tris Criterion XT pre-cast gel (Bio-Rad, 3450123) and run at constant voltage in XT 3-(N-morpholino)propanesulfonic acid (MOPS) running buffer (Bio-Rad, 161-0791). Protein was then transferred for 1 hr at 1 A constant current onto a nitrocellulose membrane. After transfer, membranes were stained with Ponceau S for 15 min at room temperature to determine total protein. Blots were then de-stained in PBS and blocked for 1 hr at room temperature in Tris-buffered saline with Tween (TBST) + 5% BSA and then incubated with primary antibodies overnight at 4°C. Western blots were performed by using primary antibodies directed against IKKβ (Abcam, 1:1,000, ab124957) and Cav1 (Abcam, 1:1,000, ab2910). Following overnight incubation, blots were washed 5 times for 5 min in TBST before being incubated in secondary antibody for 1 hr at room temperature. Goat anti-rabbit horseradish peroxidase (HRP)-conjugated secondary antibodies were purchased from Cell Signaling Technology (Danvers, MA, 7074) and used at a 1:5,000 dilution. Blots were again washed 5 times in TBST. Bands were then developed using Clarity Western ECL Substrate (Bio-Rad, 170-5061), followed by detection using a ChemiDoc Touch Imaging System (Bio-Rad). Images were quantified using Image Studio Lite software program (LI-COR Biosciences, Lincoln, NB). Total IKKβ and Cav1 expression was normalized to total protein, as determined

by Ponceau staining. Each set of HUVEC biological replicates was mean centered to account for variation between gels.

Tissue Harvesting for Whole-Mount Vascular Casting and Cross-Sectional Analysis

For the analysis of lumen diameters in the gracilis collateral arteries and to enable sectioning at specific regions, vascular casting was performed using an opaque polymer that allows for accurate lumenal diameter measurements.[88] At 21 days after FAL, mice were anesthetized (i.p., 120 mg/kg ketamine, 12 mg/kg xylazine, and 0.08 mg/kg atropine), euthanized via an overdose of pentobarbital sodium and phenytoin sodium (Euthasol), and then the abdominal aorta was cannulated. The lower body was then perfused with 7 mL 2% heparinized saline with 2 mmol/L adenosine (16404, Thermo Fisher Scientific) and 0.1 mmol/L papaverine (P3510, Sigma-Aldrich) to clear and vasodilate the downstream vasculature at a constant rate of 1 mL/min (PHD2000, Harvard Apparatus). Once perfused, we waited 5 min to enable vasodilation. Tissues were then perfused with 3 mL 4% PFA solution (19943, Affymetrix, Cleveland, OH) at 1 mL/min and allowed to fix for 10 min. The lower body was then perfused with 0.8 mL MICROFIL casting agent (Flow Tech, Carver, MA) at a constant speed of 0.15 mL/min. The viscosity of MICROFIL was adjusted to minimize transport across capillaries. After curing for 1.5 hr at room temperature, gracilis muscles were dissected free and then cleared in 50% glycerol in PBS overnight. Cleared tissues were mounted between two coverslips using 500-μm-thick spacers (645501, Grace Bio-Labs) to keep constant thickness between muscles. Muscles were imaged using transmitted light at 4× magnification on a Nikon TE200 inverted microscope with a charge coupled device (CCD) camera (Quantifier, Optronics). Individual FOV were montaged together (Photoshop CS2, Adobe Systems).

For analysis of lumen diameters from intact gracilis collateral whole mounts (i.e., vascular casting), collateral entrance regions were defined according to the following method. A cropped portion (560 × 560 μm) of the montaged image (previously randomized and de-identified) was taken of the collateral artery at the first visible branchpoint of a terminal arteriole from the primary collateral as it extended from either the muscular branch or saphenous artery, as previously described.[10] After each cropped image region was taken, all images were randomized and de-identified. The mean diameter was then taken from 4–5 separate diameter measurements along the length of cropped portion of the collateral artery.

After imaging, muscles were rehydrated, cut, and then paraffin embedded for cross-sectional analysis at the muscular branch and saphenous artery entrance regions to the collateral arteries. Resulting cross sections were rehydrated and immunolabeled for the macrophage marker Mac3 or F4/80 (day 7 post-FAL) and H&E stained for collateral artery structure analysis (day 21 post-FAL).

Cross-Sectional Analysis of Collateral Artery Structure

Sections (5-μm thickness) of paraffin-embedded muscle from the muscular and saphenous regions were labeled for H&E. Individual FOV encompassing the collateral vessels were imaged with a 40× water objective on a Zeiss inverted microscope (Zeiss Axioskop, Thornwood, NY) with a CCD camera (Quantifier, Optronics). All images were randomized and de-identified prior to analysis. Lumenal diameter, wall area, and wall thickness were determined using Fiji.[90]

Immunofluorescence Labeling of Pericollateral Macrophages

Sections (5-μm thickness) of paraffin-embedded muscle from the muscular and saphenous regions were rehydrated and subjected to heat-mediated antigen retrieval for 10 min in a citrate-based antigen retrieval buffer (Vector Laboratories, Burlingame, CA; H-3300). Slides were then quenched of endogenous peroxidase activity with a 30-min incubation in 3% H_2O_2, blocked, and labeled with rat-anti-Mac3 (1:100, M3/4 clone, 550292; BD Biosciences, San Jose, CA) overnight at 4°C. Slides were washed and incubated with a biotinylated sheep-anti-rat secondary antibody (Abcam, Cambridge, MA; ab6851, 1:500) for 1 hr at room temperature. Slides were washed and incubated with an avidin-biotin complex (Vectastain ABC solution, Vector Laboratories) for 30 min at room temperature. Slides were washed and incubated with a tyramide signal amplification (TSA) reagent (PerkinElmer, Waltham, MA; 1:50) for 10 min at room temperature. Slides were washed and incubated with streptavidin-488 (Life Technologies, 1:500), Cy3-anti-SMA (1A4 clone, Sigma, 1:1,000), and DRAQ5 (Thermo Fisher Scientific, 1:1,000) for 1 hr at room temperature. Slides were then mounted with Prolong Gold (Life Technologies) to minimize photobleaching, allowed to cure overnight, and imaged using a Nikon TE2000 C1 laser-scanning confocal microscope with a 20× oil objective. Cropped FOV (512 × 512 pixels) encompassing the collaterals in each region were randomized and de-identified. The pericollateral region was outlined (25 μm around the vessel), and pericollateral Mac3$^+$ nuclei were counted in Fiji.[90]

F4/80 staining was performed similarly as previously described.[39] Sections (5-μm thickness) of paraffin-embedded muscle from the muscular and saphenous regions were rehydrated and subjected to enzyme-mediated antigen retrieval for 15 min in 4-(2-hydroxyethyl)-1-piperazineethanesulfonic acid (HEPES)-buffered saline containing 0.05% trypsin and 10 μg/mL proteinase K. Sections were then quenched of endogenous peroxidase activity with a 30-min incubation in 3% H_2O_2. Slides were washed and incubated with anti-mouse CD16/CD32 Fc blocker (BD Biosciences, San Jose, CA; 1:100) for 30 min, and then blocked in PBS with 2% BSA and 5% normal goat serum for 1 hr. Endogenous biotin was also blocked, using an avidin-biotin-blocking kit as per the manufacturer's instructions (Thermo Fisher Scientific, 004303). Slides were then incubated overnight at 4°C with a rat anti-F4/80 antibody (CI:A3-1 clone, Bio-Rad, 1:100). Following overnight incubation, slides were washed and incubated with a biotinylated sheep-anti-rat antibody (Abcam, ab6851, 1:200) for 1 hr at room temperature. Following secondary antibody incubation, slides were incubated in streptavidin-conjugated horseradish peroxidase (Tyramide Signal Amplification kit, PerkinElmer, Waltham, MA; 1:200) for 1 hr at room temperature. Slides were washed and incubated with a TSA reagent (PerkinElmer,

Waltham, MA; 1:50) for 10 min at room temperature. Slides were washed and incubated with streptavidin-647 (Thermo Scientific, 1:200), Cy3-anti-SMA (1A4 clone, Sigma, 1:1,000), and Sytox Green (Thermo Scientific, 10 nM). Slides were then mounted with Prolong Diamond (Life Technologies) to minimize photobleaching, allowed to cure overnight, and imaged using a Nikon TE2000 C1 laser-scanning confocal microscope with a 20× oil objective. Cropped FOV (512 × 512 pixels) encompassing the collaterals in each region were randomized and de-identified. The pericollateral region was outlined (25 μm around the vessel), and pericollateral F4/80$^+$ nuclei were counted in Fiji.

Cross-Sectional Analysis for Gastrocnemius Muscle Morphology

For H&E analysis, sections (5-μm thickness) of paraffin-embedded muscle from the gastrocnemius muscle were H&E labeled. Individual FOV were imaged with a 10× objective on a Zeiss inverted microscope (Zeiss Axioskop) with a CCD camera (Quantifier, Optronics). Individual FOV were montaged together (Photoshop CS2, Adobe Systems). All montaged images were randomized and de-identified prior to analysis. Muscle areas were manually outlined using Fiji. Tissue composition was classified into viable and non-viable, which were defined as follows.

> Viable: fibers are present and have centrally located nuclei (regenerating) or fibers are comparable in size, organization, and structure to unligated control with peripheral nuclei (mature).[8]
>
> Non-viable: fibers lack nuclei, are rounded and dilated in appearance, have weak eosinophilic cytoplasm (necrotic), or there is a minimal presence of myoblasts and dominant fibrous matrix and adipose tissue (fibro-adipose).[8]

For Masson trichrome analysis, sections (5-μm thickness) of paraffin-embedded muscle from the muscular and saphenous regions were stained with Masson trichrome (iron hematoxylin, aniline blue, and Biebrich scarlet-acid fuchsin). Individual FOV were imaged using a Zeiss Axioskop transmitted light microscope with a 4× objective and a Jenoptik Gryphax camera, and then they were montaged together (Photoshop CS2, Adobe Systems). The tissue sections were outlined in the resulting images, and the pixels within the outline were analyzed with a R script written by the authors to assess the number of pixels with a significantly greater (>170%) blue than red intensity (indicating fibrotic tissue)[91] in order to semiquantitatively assess the extent of fibrosis in each section.

Cross-Sectional Analysis for Gastrocnemius Capillary Density

Sections (5-μm thickness) of paraffin-embedded gastrocnemius muscles were deparaffinized, rehydrated, then blocked in Carbofree blocking solution (1:10, Vector Laboratories). Slides were then incubated with fluorophore-conjugated primary antibody (isolectin-IB4-AlexFluor-647, 1:200, Life Technologies) overnight at 4°C. Nuclei were counterstained with Sytox green (500 μM, Life Technologies). Slides were washed and sealed with Prolong Gold (Life Technologies) to minimize photobleaching. Individual FOV were imaged with a Nikon TE2000 C1 laser-scanning confocal microscope with a 10× objective and the same imaging parameters for all FOV. FOV were then montaged together using Photoshop CS2 (Adobe Systems). Muscle areas were manually outlined using Fiji and classified as either viable or non-viable tissue. The numbers of capillaries (Isolectin-B4$^+$ vessels <25 μm^2 in diameter) and muscle area (identified from autofluorescence) were determined in each montaged image view using a semi-automated Fiji image analysis.

Human Patient Population and Plasma Collection

All protocols were approved by the Institutional Review Board at the University of Virginia. Plasma was collected from an equal number of patients exhibiting intermittent claudication or as risk factor (e.g., diabetic, hypertensive, hyperlipidemic, and smoker) controls (n = 25/group). A diagnosis of PAD with intermittent claudication was based on having one of the following: (1) an ABI < 0.9; (2) a previous peripheral vascular intervention; or (3) an abnormal TBI, without any corresponding foot ulcers or resting pain. Total RNA was purified from 200 μL plasma using the miRCURY Biofluids RNA isolation kit (Exiqon, 300112). For reverse transcription (Applied Biosystems, 4366596) and miR-199a-5p detection, TaqMan miRNA assays (Applied Biosystems, 4427975) were used. RT-PCR was performed using the SensiMix II probe kit (Bioline) on an CFX96 detection system (Bio-Rad). Gene expression was normalized to RNU6 (Applied Biosystems, 4427975). Relative expression was determined with the comparative $2^{\Delta\Delta Ct}$ method and is represented as the $\log_2$ fold change. The $\log_2$ fold change data were normally distributed as determined by a Shapiro-Wilk test was tested for statistical significance using a two-sided Student's t test.

C2C12 Viability Assay

C2C12 myoblasts were plated at 1×10^4 cells/well in a 96-well plate, and they were transfected with 20 nM scramble or miR-199a-5p LNA inhibitors (Exiqon) using siPORT NeoFX transfection agent (AM4510, Thermo Fisher Scientific), according to the manufacturer's instructions. At 24 hr after transfection, cell culture medium was changed to serum starvation medium (209-250, Cell Applications) supplemented with tetrazolium salt WST-1/ECS solution (K301, BioVision Technologies, Milipitas, CA). Cells were incubated in a 2% oxygen chamber (BioSpherix, Lacona, NY) under hypoxia serum starvation (HSS) conditions for 6 hr. This time point was chosen as previous studies have shown significant C2C12 cell death under HSS conditions at later time points.[23] After 6 hr, cell viability was assessed as the resulting tetrazolium salt cleavage to formazan by mitochondrial dehydrogenases by measuring absorbance at 450 nm.

Statistical Analyses

All results are reported as mean ± SEM, unless otherwise noted. All data were first tested for normality using a Shapiro-Wilk test followed by equal variance. Statistical significance was then assessed by a Student's t test or a two-way ANOVA followed by a Holm-Sidak multiple comparisons test, unless otherwise noted (SigmaStat 3.5, Systat). Significance was assessed at $p < 0.05$.

SUPPLEMENTAL INFORMATION

Supplemental Information includes eleven figures and two tables and can be found with this article online at https://doi.org/10.1016/j.omtn.2018.08.001.

AUTHOR CONTRIBUTIONS

Conceptualization, J.L.H. and R.J.P.; Methodology, J.L.H. and R.J.P.; Investigation, J.L.H., C.M.G., S.P.M., and J.S.; Formal Analysis, J.L.H., C.M.G., and R.J.P.; Resources, B.H.A.; Writing – Original Draft, J.L.H. and R.J.P.; Writing – Review & Editing, J.L.H., C.M.G., S.P.M., J.S., B.H.A., and R.J.P.; Visualization, J.L.H. and R.J.P.; Supervision, B.H.A. and R.J.P.; Funding Acquisition, J.L.H., B.H.A., and R.J.P.

CONFLICTS OF INTEREST

The authors declare no competing interests.

ACKNOWLEDGMENTS

The authors would like to thank the University of Virginia Research Histology Core (under the direction of Sheri VanHoose) for histological tissue processing and the DNA Sciences Core (under the direction of Yongde Bao), which is supported by the University of Virginia School of Medicine, for running TaqMan miRNA qRT-PCR plates. This work was supported by the NIH (grants T32HLI007284 to J.L.H., R03EB017927 to R.J.P., R01EB020147 to R.J.P., R21EB024323 to R.J.P., R01HL116455 to B.H.A., R01HL121635 to B.H.A., and R01HL101200 to B.H.A.) and the National Science Foundation (grant DGE-1315231 to J.L.H.).

REFERENCES

1. Fowkes, F.G.R., Rudan, D., Rudan, I., Aboyans, V., Denenberg, J.O., McDermott, M.M., Norman, P.E., Sampson, U.K., Williams, L.J., Mensah, G.A., and Criqui, M.H. (2013). Comparison of global estimates of prevalence and risk factors for peripheral artery disease in 2000 and 2010: a systematic review and analysis. Lancet *382*, 1329–1340.

2. Annex, B.H. (2013). Therapeutic angiogenesis for critical limb ischaemia. Nat. Rev. Cardiol. *10*, 387–396.

3. Meisner, J.K., and Price, R.J. (2010). Spatial and temporal coordination of bone marrow-derived cell activity during arteriogenesis: regulation of the endogenous response and therapeutic implications. Microcirculation *17*, 583–599.

4. Robbins, J.L., Jones, W.S., Duscha, B.D., Allen, J.D., Kraus, W.E., Regensteiner, J.G., Hiatt, W.R., and Annex, B.H. (2011). Relationship between leg muscle capillary density and peak hyperemic blood flow with endurance capacity in peripheral artery disease. J. Appl. Physiol. *111*, 81–86.

5. Duscha, B.D., Robbins, J.L., Jones, W.S., Kraus, W.E., Lye, R.J., Sanders, J.M., Allen, J.D., Regensteiner, J.G., Hiatt, W.R., and Annex, B.H. (2011). Angiogenesis in skeletal muscle precede improvements in peak oxygen uptake in peripheral artery disease patients. Arterioscler. Thromb. Vasc. Biol. *31*, 2742–2748.

6. Heuslein, J.L., Li, X., Murrell, K.P., Annex, B.H., Peirce, S.M., and Price, R.J. (2015). Computational network model prediction of hemodynamic alterations due to arteriolar rarefaction and estimation of skeletal muscle perfusion in peripheral arterial disease. Microcirculation *22*, 360–369.

7. Meisner, J.K., Song, J., Annex, B.H., and Price, R.J. (2013). Myoglobin overexpression inhibits reperfusion in the ischemic mouse hindlimb through impaired angiogenesis but not arteriogenesis. Am. J. Pathol. *183*, 1710–1718.

8. Meisner, J.K., Annex, B.H., and Price, R.J. (2015). Despite normal arteriogenic and angiogenic responses, hind limb perfusion recovery and necrotic and fibroadipose tissue clearance are impaired in matrix metalloproteinase 9-deficient mice. J. Vasc. Surg. *61*, 1583, 94.e1, 10.

9. Schaper, W. (2009). Collateral circulation: past and present. Basic Res. Cardiol. *104*, 5–21.

10. Heuslein, J.L., Meisner, J.K., Li, X., Song, J., Vincentelli, H., Leiphart, R.J., Ames, E.G., Blackman, B.R., Blackman, B.R., and Price, R.J. (2015). Mechanisms of amplified arteriogenesis in collateral artery segments exposed to reversed flow direction. Arterioscler. Thromb. Vasc. Biol. *35*, 2354–2365.

11. Meisner, J.K., Niu, J., Sumer, S., and Price, R.J. (2013). Trans-illuminated laser speckle imaging of collateral artery blood flow in ischemic mouse hindlimb. J. Biomed. Opt. *18*, 096011.

12. Neth, P., Nazari-Jahantigh, M., Schober, A., and Weber, C. (2013). MicroRNAs in flow-dependent vascular remodelling. Cardiovasc. Res. *99*, 294–303.

13. Urbich, C., Kuehbacher, A., and Dimmeler, S. (2008). Role of microRNAs in vascular diseases, inflammation, and angiogenesis. Cardiovasc. Res. *79*, 581–588.

14. Bartel, D.P. (2004). MicroRNAs: genomics, biogenesis, mechanism, and function. Cell *116*, 281–297.

15. Ha, M., and Kim, V.N. (2014). Regulation of microRNA biogenesis. Nat. Rev. Mol. Cell Biol. *15*, 509–524.

16. Jonas, S., and Izaurralde, E. (2015). Towards a molecular understanding of microRNA-mediated gene silencing. Nat. Rev. Genet. *16*, 421–433.

17. Lewis, B.P., Shih, I.H., Jones-Rhoades, M.W., Bartel, D.P., and Burge, C.B. (2003). Prediction of mammalian microRNA targets. Cell *115*, 787–798.

18. van Rooij, E. (2011). The art of microRNA research. Circ. Res. *108*, 219–234.

19. Bonauer, A., Carmona, G., Iwasaki, M., Mione, M., Koyanagi, M., Fischer, A., Burchfield, J., Fox, H., Doebele, C., Ohtani, K., et al. (2009). MicroRNA-92a controls angiogenesis and functional recovery of ischemic tissues in mice. Science *324*, 1710–1713.

20. Grundmann, S., Hans, F.P., Kinniry, S., Heinke, J., Helbing, T., Bluhm, F., Sluijter, J.P., Hoefer, I., Pasterkamp, G., Bode, C., and Moser, M. (2011). MicroRNA-100 regulates neovascularization by suppression of mammalian target of rapamycin in endothelial and vascular smooth muscle cells. Circulation *123*, 999–1009.

21. Caporali, A., Meloni, M., Völlenkle, C., Bonci, D., Sala-Newby, G.B., Addis, R., Spinetti, G., Losa, S., Masson, R., Baker, A.H., et al. (2011). Deregulation of microRNA-503 contributes to diabetes mellitus-induced impairment of endothelial function and reparative angiogenesis after limb ischemia. Circulation *123*, 282–291.

22. Yin, K.J., Olsen, K., Hamblin, M., Zhang, J., Schwendeman, S.P., and Chen, Y.E. (2012). Vascular endothelial cell-specific microRNA-15a inhibits angiogenesis in hindlimb ischemia. J. Biol. Chem. *287*, 27055–27064.

23. Hazarika, S., Farber, C.R., Dokun, A.O., Pitsillides, A.N., Wang, T., Lye, R.J., and Annex, B.H. (2013). MicroRNA-93 controls perfusion recovery after hindlimb ischemia by modulating expression of multiple genes in the cell cycle pathway. Circulation *127*, 1818–1828.

24. Welten, S.M., Bastiaansen, A.J., de Jong, R.C., de Vries, M.R., Peters, E.A., Boonstra, M.C., Sheikh, S.P., La Monica, N., Kandimalla, E.R., Quax, P.H., and Nossent, A.Y. (2014). Inhibition of 14q32 MicroRNAs miR-329, miR-487b, miR-494, and miR-495 increases neovascularization and blood flow recovery after ischemia. Circ. Res. *115*, 696–708.

25. Cao, W.J., Rosenblat, J.D., Roth, N.C., Kuliszewski, M.A., Matkar, P.N., Rudenko, D., Liao, C., Lee, P.J., and Leong-Poi, H. (2015). Therapeutic angiogenesis by ultrasound-mediated microRNA-126-3p delivery. Arterioscler. Thromb. Vasc. Biol. *35*, 2401–2411.

26. Pankratz, F., Bemtgen, X., Zeiser, R., Leonhardt, F., Kreuzaler, S., Hilgendorf, I., Smolka, C., Helbing, T., Hoefer, I., Esser, J.S., et al. (2015). MicroRNA-155 exerts cell-specific antiangiogenic but proarteriogenic effects during adaptive neovascularization. Circulation *131*, 1575–1589.

27. Landskroner-Eiger, S., Qiu, C., Perrotta, P., Siragusa, M., Lee, M.Y., Ulrich, V., Luciano, A.K., Zhuang, Z.W., Corti, F., Simons, M., et al. (2015). Endothelial miR-17∼92 cluster negatively regulates arteriogenesis via miRNA-19 repression of WNT signaling. Proc. Natl. Acad. Sci. USA *112*, 12812–12817.

28. Chiu, J.-J., and Chien, S. (2011). Effects of disturbed flow on vascular endothelium: pathophysiological basis and clinical perspectives. Physiol. Rev. *91*, 327–387.

29. Chen, R., Alvero, A.B., Silasi, D.A., Kelly, M.G., Fest, S., Visintin, I., Leiser, A., Schwartz, P.E., Rutherford, T., and Mor, G. (2008). Regulation of IKKbeta by miR-199a affects NF-kappaB activity in ovarian cancer cells. Oncogene *27*, 4712–4723.

30. Bauer, P.M., Yu, J., Chen, Y., Hickey, R., Bernatchez, P.N., Looft-Wilson, R., Huang, Y., Giordano, F., Stan, R.V., and Sessa, W.C. (2005). Endothelial-specific expression of caveolin-1 impairs microvascular permeability and angiogenesis. Proc. Natl. Acad. Sci. USA *102*, 204–209.

31. Sonveaux, P., Martinive, P., DeWever, J., Batova, Z., Daneau, G., Pelat, M., Ghisdal, P., Grégoire, V., Dessy, C., Balligand, J.L., and Feron, O. (2004). Caveolin-1 expression is critical for vascular endothelial growth factor-induced ischemic hindlimb collateralization and nitric oxide-mediated angiogenesis. Circ. Res. *95*, 154–161.

32. Hoefer, I.E., van Royen, N., Rectenwald, J.E., Deindl, E., Hua, J., Jost, M., Grundmann, S., Voskuil, M., Ozaki, C.K., Piek, J.J., and Buschmann, I.R. (2004). Arteriogenesis proceeds via ICAM-1/Mac-1- mediated mechanisms. Circ. Res. *94*, 1179–1185.

33. Buschmann, I.R., Hoefer, I.E., van Royen, N., Katzer, E., Braun-Dulleaus, R., Heil, M., Kostin, S., Bode, C., and Schaper, W. (2001). GM-CSF: a strong arteriogenic factor acting by amplification of monocyte function. Atherosclerosis *159*, 343–356.

34. Hoefer, I.E., Grundmann, S., van Royen, N., Voskuil, M., Schirmer, S.H., Ulusans, S., Bode, C., Buschmann, I.R., and Piek, J.J. (2005). Leukocyte subpopulations and arteriogenesis: specific role of monocytes, lymphocytes and granulocytes. Atherosclerosis *181*, 285–293.

35. Bergmann, C.E., Hoefer, I.E., Meder, B., Roth, H., van Royen, N., Breit, S.M., Jost, M.M., Aharinejad, S., Hartmann, S., and Buschmann, I.R. (2006). Arteriogenesis depends on circulating monocytes and macrophage accumulation and is severely depressed in op/op mice. J. Leukoc. Biol. *80*, 59–65.

36. Arras, M., Ito, W.D., Scholz, D., Winkler, B., Schaper, J., and Schaper, W. (1998). Monocyte activation in angiogenesis and collateral growth in the rabbit hindlimb. J. Clin. Invest. *101*, 40–50.

37. Scholz, D., Ito, W., Fleming, I., Deindl, E., Sauer, A., Wiesnet, M., Busse, R., Schaper, J., and Schaper, W. (2000). Ultrastructure and molecular histology of rabbit hindlimb collateral artery growth (arteriogenesis). Virchows Arch. *436*, 257–270.

38. Bruce, A.C., Kelly-Goss, M.R., Heuslein, J.L., Meisner, J.K., Price, R.J., and Peirce, S.M. (2014). Monocytes are recruited from venules during arteriogenesis in the murine spinotrapezius ligation model. Arterioscler. Thromb. Vasc. Biol. *34*, 2012–2022.

39. Inoue, T., Plieth, D., Venkov, C.D., Xu, C., and Neilson, E.G. (2005). Antibodies against macrophages that overlap in specificity with fibroblasts. Kidney Int. *67*, 2488–2493.

40. Spallotta, F., Tardivo, S., Nanni, S., Rosati, J.D., Straino, S., Mai, A., Vecellio, M., Valente, S., Capogrossi, M.C., Farsetti, A., et al. (2013). Detrimental effect of class-selective histone deacetylase inhibitors during tissue regeneration following hindlimb ischemia. J. Biol. Chem. *288*, 22915–22929.

41. Lian, Q., Zhang, Y., Zhang, J., Zhang, H.K., Wu, X., Zhang, Y., Lam, F.F., Kang, S., Xia, J.C., Lai, W.H., et al. (2010). Functional mesenchymal stem cells derived from human induced pluripotent stem cells attenuate limb ischemia in mice. Circulation *121*, 1113–1123.

42. Heuslein, J.L., McDonnell, S.P., Song, J., Annex, B.H., and Price, R.J. (2018). MicroRNA-146a Regulates Perfusion Recovery in Response to Arterial Occlusion *via* Arteriogenesis. Front. Bioeng. Biotechnol. *6*, 1–13.

43. Epstein, S.E., Stabile, E., Kinnaird, T., Lee, C.W., Clavijo, L., and Burnett, M.S. (2004). Janus phenomenon: the interrelated tradeoffs inherent in therapies designed to enhance collateral formation and those designed to inhibit atherogenesis. Circulation *109*, 2826–2831.

44. Raitoharju, E., Lyytikäinen, L.-P., Levula, M., Oksala, N., Mennander, A., Tarkka, M., Klopp, N., Illig, T., Kähönen, M., Karhunen, P.J., et al. (2011). miR-21, miR-210, miR-34a, and miR-146a/b are up-regulated in human atherosclerotic plaques in the Tampere Vascular Study. Atherosclerosis *219*, 211–217.

45. Cheng, H.S., Besla, R., Li, A., Chen, Z., Shikatani, E.A., Nazari-Jahantigh, M., Hammoutène, A., Nguyen, M.A., Geoffrion, M., Cai, L., et al. (2017). Paradoxical suppression of atherosclerosis in the absence of microRNA-146a. Circ. Res. *121*, 354–367.

46. Song, X.W., Li, Q., Lin, L., Wang, X.C., Li, D.F., Wang, G.K., Ren, A.J., Wang, Y.R., Qin, Y.W., Yuan, W.J., and Jing, Q. (2010). MicroRNAs are dynamically regulated in hypertrophic hearts, and miR-199a is essential for the maintenance of cell size in cardiomyocytes. J. Cell. Physiol. *225*, 437–443.

47. Li, Z., Song, Y., Liu, L., Hou, N., An, X., Zhan, D., Li, Y., Zhou, L., Li, P., Yu, L., et al. (2017). miR-199a impairs autophagy and induces cardiac hypertrophy through mTOR activation. Cell Death Differ. *24*, 1205–1213.

48. Hashemi Gheinani, A., Burkhard, F.C., Rehrauer, H., Aquino Fournier, C., and Monastyrskaya, K. (2015). MicroRNA MiR-199a-5p regulates smooth muscle cell proliferation and morphology by targeting WNT2 signaling pathway. J. Biol. Chem. *290*, 7067–7086.

49. Chan, Y.C., Roy, S., Huang, Y., Khanna, S., and Sen, C.K. (2012). The microRNA miR-199a-5p down-regulation switches on wound angiogenesis by derepressing the v-ets erythroblastosis virus E26 oncogene homolog 1-matrix metalloproteinase-1 pathway. J. Biol. Chem. *287*, 41032–41043.

50. Ding, G., Huang, G., Liu, H.D., Liang, H.X., Ni, Y.F., Ding, Z.H., Ni, G.Y., and Hua, H.W. (2013). MiR-199a suppresses the hypoxia-induced proliferation of non-small cell lung cancer cells through targeting HIF1α. Mol. Cell. Biochem. *384*, 173–180.

51. He, J., Jing, Y., Li, W., Qian, X., Xu, Q., Li, F.S., Liu, L.Z., Jiang, B.H., and Jiang, Y. (2013). Roles and mechanism of miR-199a and miR-125b in tumor angiogenesis. PLoS ONE *8*, e56647.

52. Raimondi, L., Amodio, N., Di Martino, M.T., Altomare, E., Leotta, M., Caracciolo, D., Gullà, A., Neri, A., Taverna, S., D'Aquila, P., et al. (2014). Targeting of multiple myeloma-related angiogenesis by miR-199a-5p mimics: in vitro and in vivo anti-tumor activity. Oncotarget *5*, 3039–3054.

53. Dai, L., Lou, W., Zhu, J., Zhou, X., and Di, W. (2015). MiR-199a inhibits the angiogenic potential of endometrial stromal cells under hypoxia by targeting HIF-1α/VEGF pathway. Int. J. Clin. Exp. Pathol. *8*, 4735–4744.

54. Liu, G.-T., Huang, Y.-L., Tzeng, H.-E., Tsai, C.-H., Wang, S.-W., and Tang, C.-H. (2015). CCL5 promotes vascular endothelial growth factor expression and induces angiogenesis by down-regulating miR-199a in human chondrosarcoma cells. Cancer Lett. *357*, 476–487.

55. Cheng, W., Liu, T., Wan, X., Gao, Y., and Wang, H. (2012). MicroRNA-199a targets CD44 to suppress the tumorigenicity and multidrug resistance of ovarian cancer-initiating cells. FEBS J. *279*, 2047–2059.

56. Song, J., Gao, L., Yang, G., Tang, S., Xie, H., Wang, Y., Wang, J., Zhang, Y., Jin, J., Gou, Y., et al. (2014). MiR-199a regulates cell proliferation and survival by targeting FZD7. PLoS ONE *9*, e110074.

57. Zhang, L.F., Lou, J.T., Lu, M.H., Gao, C., Zhao, S., Li, B., Liang, S., Li, Y., Li, D., and Liu, M.F. (2015). Suppression of miR-199a maturation by HuR is crucial for hypoxia-induced glycolytic switch in hepatocellular carcinoma. EMBO J. *34*, 2671–2685.

58. Gui, R., Huang, R., Zhang, J.-H., Wen, X.-H., and Nie, X.-M. (2016). MicroRNA-199a-5p inhibits VEGF-induced tumorigenesis through targeting oxidored-nitro domain-containing protein 1 in human HepG2 cells. Oncol. Rep. *35*, 2216–2222.

59. Yang, X., Lei, S., Long, J., Liu, X., and Wu, Q. (2016). MicroRNA-199a-5p inhibits tumor proliferation in melanoma by mediating HIF-1α. Mol. Med. Rep. *13*, 5241–5247.

60. Tirziu, D., Jaba, I.M., Yu, P., Larrivée, B., Coon, B.G., Cristofaro, B., Zhuang, Z.W., Lanahan, A.A., Schwartz, M.A., Eichmann, A., and Simons, M. (2012). Endothelial nuclear factor-κB-dependent regulation of arteriogenesis and branching. Circulation *126*, 2589–2600.

61. Sun, R.J., Muller, S., Stoltz, J.F., and Wang, X. (2002). Shear stress induces caveolin-1 translocation in cultured endothelial cells. Eur. Biophys. J. *30*, 605–611.

62. Jiang, Y., Lin, X., Tang, Z., Lee, C., Tian, G., Du, Y., Yin, X., Ren, X., Huang, L., Ye, Z., et al. (2017). Critical role of caveolin-1 in ocular neovascularization and multitargeted antiangiogenic effects of cavtratin via JNK. Proc. Natl. Acad. Sci. USA *114*, 10737–10742.

63. Zhang, P.X., Cheng, J., Zou, S., D'Souza, A.D., Koff, J.L., Lu, J., Lee, P.J., Krause, D.S., Egan, M.E., and Bruscia, E.M. (2015). Pharmacological modulation of the AKT/microRNA-199a-5p/CAV1 pathway ameliorates cystic fibrosis lung hyper-inflammation. Nat. Commun. *6*, 6221.

64. Shi, X.-E., Li, Y.-F., Jia, L., Ji, H.-L., Song, Z.-Y., Cheng, J., Wu, G.F., Song, C.C., Zhang, Q.L., Zhu, J.Y., and Yang, G.S. (2014). MicroRNA-199a-5p affects porcine preadipocyte proliferation and differentiation. Int. J. Mol. Sci. *15*, 8526–8538.

65. Zeng, J., Chen, L., Chen, B., Lu, K., Belguise, K., Wang, X., and Yi, B. (2015). MicroRNA-199a-5p Regulates the Proliferation of Pulmonary Microvascular Endothelial Cells in Hepatopulmonary Syndrome. Cell. Physiol. Biochem. *37*, 1289–1300.

66. Lino Cardenas, C.L., Henaoui, I.S., Courcot, E., Roderburg, C., Cauffiez, C., Aubert, S., Copin, M.C., Wallaert, B., Glowacki, F., Dewaeles, E., et al. (2013). miR-199a-5p Is upregulated during fibrogenic response to tissue injury and mediates TGFbeta-induced lung fibroblast activation by targeting caveolin-1. PLoS Genet. *9*, e1003291.

67. Aranda, J.F., Canfrán-Duque, A., Goedeke, L., Suárez, Y., and Fernández-Hernando, C. (2015). The miR-199-dynamin regulatory axis controls receptor-mediated endocytosis. J. Cell Sci. *128*, 3197–3209.

68. Ganta, V.C., Choi, M.H., Kutateladze, A., Fox, T.E., Farber, C.R., and Annex, B.H. (2017). A microRNA93-interferon regulatory factor-9-immunoresponsive gene-1-itaconic acid pathway modulates M2-like macrophage polarization to revascularize ischemic muscle. Circulation *135*, 2403–2425.

69. Guan, Y., Cai, B., Wu, X., Peng, S., Gan, L., Huang, D., Liu, G., Dong, L., Xiao, L., Liu, J., et al. (2017). microRNA-352 regulates collateral vessel growth induced by elevated fluid shear stress in the rat hind limb. Sci. Rep. *7*, 6643.

70. Scholz, D., Ziegelhoeffer, T., Helisch, A., Wagner, S., Friedrich, C., Podzuweit, T., and Schaper, W. (2002). Contribution of arteriogenesis and angiogenesis to postocclusive hindlimb perfusion in mice. J. Mol. Cell. Cardiol. *34*, 775–787.

71. Helisch, A., Wagner, S., Khan, N., Drinane, M., Wolfram, S., Heil, M., Ziegelhoeffer, T., Brandt, U., Pearlman, J.D., Swartz, H.M., and Schaper, W. (2006). Impact of mouse strain differences in innate hindlimb collateral vasculature. Arterioscler. Thromb. Vasc. Biol. *26*, 520–526.

72. van Solingen, C., Seghers, L., Bijkerk, R., Duijs, J.M.G.J., Roeten, M.K., van Oeveren-Rietdijk, A.M., Baelde, H.J., Monge, M., Vos, J.B., de Boer, H.C., et al. (2009). Antagomir-mediated silencing of endothelial cell specific microRNA-126 impairs ischemia-induced angiogenesis. J. Cell. Mol. Med. *13* (8A), 1577–1585.

73. Heil, M., Ziegelhoeffer, T., Pipp, F., Kostin, S., Martin, S., Clauss, M., and Schaper, W. (2002). Blood monocyte concentration is critical for enhancement of collateral artery growth. Am. J. Physiol. Heart Circ. Physiol. *283*, H2411–H2419.

74. Alexander, M.S., Kawahara, G., Motohashi, N., Casar, J.C., Eisenberg, I., Myers, J.A., Gasperini, M.J., Estrella, E.A., Kho, A.T., Mitsuhashi, S., et al. (2013). MicroRNA-199a is induced in dystrophic muscle and affects WNT signaling, cell proliferation, and myogenic differentiation. Cell Death Differ. *20*, 1194–1208.

75. Oskowitz, A.Z., Lu, J., Penfornis, P., Ylostalo, J., McBride, J., Flemington, E.K., Prockop, D.J., and Pochampally, R. (2008). Human multipotent stromal cells from bone marrow and microRNA: regulation of differentiation and leukemia inhibitory factor expression. Proc. Natl. Acad. Sci. USA *105*, 18372–18377.

76. Lau, J.F., Weinberg, M.D., and Olin, J.W. (2011). Peripheral artery disease. Part 1: clinical evaluation and noninvasive diagnosis. Nat. Rev. Cardiol. *8*, 405–418.

77. D'Alessandra, Y., Carena, M.C., Spazzafumo, L., Martinelli, F., Bassetti, B., Devanna, P., Rubino, M., Marenzi, G., Colombo, G.I., Achilli, F., et al. (2013). Diagnostic potential of plasmatic MicroRNA signatures in stable and unstable angina. PLoS ONE *8*, e80345.

78. Regensteiner, J.G., Wolfel, E.E., Brass, E.P., Carry, M.R., Ringel, S.P., Hargarten, M.E., Stamm, E.R., and Hiatt, W.R. (1993). Chronic changes in skeletal muscle histology and function in peripheral arterial disease. Circulation *87*, 413–421.

79. Hedberg, B., Angquist, K.-A., Henriksson-Larsen, K., and Sjöström, M. (1989). Fibre loss and distribution in skeletal muscle from patients with severe peripheral arterial insufficiency. Eur. J. Vasc. Surg. *3*, 315–322.

80. Mitchell, R.G., Duscha, B.D., Robbins, J.L., Redfern, S.I., Chung, J., Bensimhon, D.R., Kraus, W.E., Hiatt, W.R., Regensteiner, J.G., and Annex, B.H. (2007). Increased levels of apoptosis in gastrocnemius skeletal muscle in patients with peripheral arterial disease. Vasc. Med. *12*, 285–290.

81. Koutakis, P., Myers, S.A., Cluff, K., Ha, D.M., Haynatzki, G., McComb, R.D., Uchida, K., Miserlis, D., Papoutsi, E., Johanning, J.M., et al. (2015). Abnormal myofiber morphology and limb dysfunction in claudication. J. Surg. Res. *196*, 172–179.

82. Blackman, B.R., García-Cardeña, G., and Gimbrone, M.A., Jr. (2002). A new in vitro model to evaluate differential responses of endothelial cells to simulated arterial shear stress waveforms. J. Biomech. Eng. *124*, 397–407.

83. Garcia-Cardeña, G., Comander, J., Anderson, K.R., Blackman, B.R., and Gimbrone, M.A., Jr. (2001). Biomechanical activation of vascular endothelium as a determinant of its functional phenotype. Proc. Natl. Acad. Sci. USA *98*, 4478–4485.

84. Meisner, J.K., Sumer, S., Murrell, K.P., Higgins, T.J., and Price, R.J. (2012). Laser speckle flowmetry method for measuring spatial and temporal hemodynamic alterations throughout large microvascular networks. Microcirculation *19*, 619–631.

85. Heuslein, J.L., Murrell, K.P., Leiphart, R.J., Llewellyn, R.A., Meisner, J.K., and Price, R.J. (2016). Vascular growth responses to chronic arterial occlusion are unaffected by myeloid specific focal adhesion kinase (FAK) deletion. Sci. Rep. *6*, 27029.

86. Chappell, J.C., Song, J., Burke, C.W., Klibanov, A.L., and Price, R.J. (2008). Targeted delivery of nanoparticles bearing fibroblast growth factor-2 by ultrasonic microbubble destruction for therapeutic arteriogenesis. Small *4*, 1769–1777.

87. Nickerson, M.M., Burke, C.W., Meisner, J.K., Shuptrine, C.W., Song, J., and Price, R.J. (2009). Capillary arterialization requires the bone-marrow-derived cell (BMC)-specific expression of chemokine (C-C motif) receptor-2, but BMCs do not transdifferentiate into microvascular smooth muscle. Angiogenesis *12*, 355–363.

88. Distasi, M.R., Case, J., Ziegler, M.A., Dinauer, M.C., Yoder, M.C., Haneline, L.S., Dalsing, M.C., Miller, S.J., Labarrere, C.A., Murphy, M.P., et al. (2009). Suppressed hindlimb perfusion in Rac2-/- and Nox2-/- mice does not result from impaired collateral growth. Am. J. Physiol. Heart Circ. Physiol. *296*, H877–H886.

89. Dai, X., and Faber, J.E. (2010). Endothelial nitric oxide synthase deficiency causes collateral vessel rarefaction and impairs activation of a cell cycle gene network during arteriogenesis. Circ. Res. *106*, 1870–1881.

90. Schindelin, J., Arganda-Carreras, I., Frise, E., Kaynig, V., Longair, M., Pietzsch, T., Preibisch, S., Rueden, C., Saalfeld, S., Schmid, B., et al. (2012). Fiji: an open-source platform for biological-image analysis. Nat. Methods *9*, 676–682.

91. Kennedy, D.J., Vetteth, S., Periyasamy, S.M., Kanj, M., Fedorova, L., Khouri, S., Kahaleh, M.B., Xie, Z., Malhotra, D., Kolodkin, N.I., et al. (2006). Central role for the cardiotonic steroid marinobufagenin in the pathogenesis of experimental uremic cardiomyopathy. Hypertension *47*, 488–495.

OMTN, Volume *12*

Supplemental Information

Exposure of Endothelium to Biomimetic Flow Waveforms Yields Identification of miR-199a-5p as a Potent Regulator of Arteriogenesis

Joshua L. Heuslein, Catherine M. Gorick, Stephanie P. McDonnell, Ji Song, Brian H. Annex, and Richard J. Price

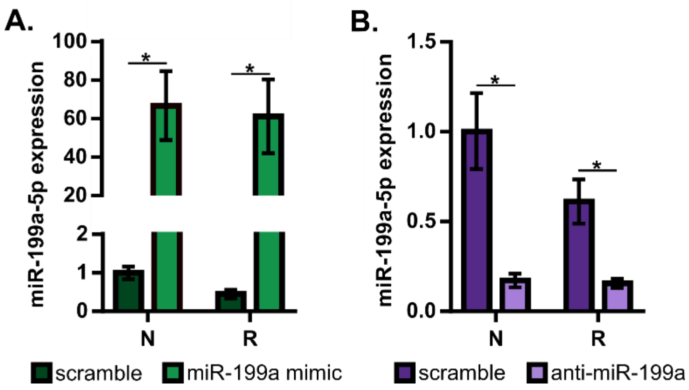

Figure S1. Validation of altered miR-199a-5p expression in transfected HUVECs.
A, B Bar graph of relative miR-199a expression 6-hours after simulated FAL in HUVECs transfected with (A) miR-199a mimic, (B) anti-miR-199a or the respective scramble controls and subjected to non-reversed (N) or reversed (R) flow waveforms as outlined in Figure 1*B* (n=4). *p<0.05, Student's *t*-test. Data are mean ± SEM.

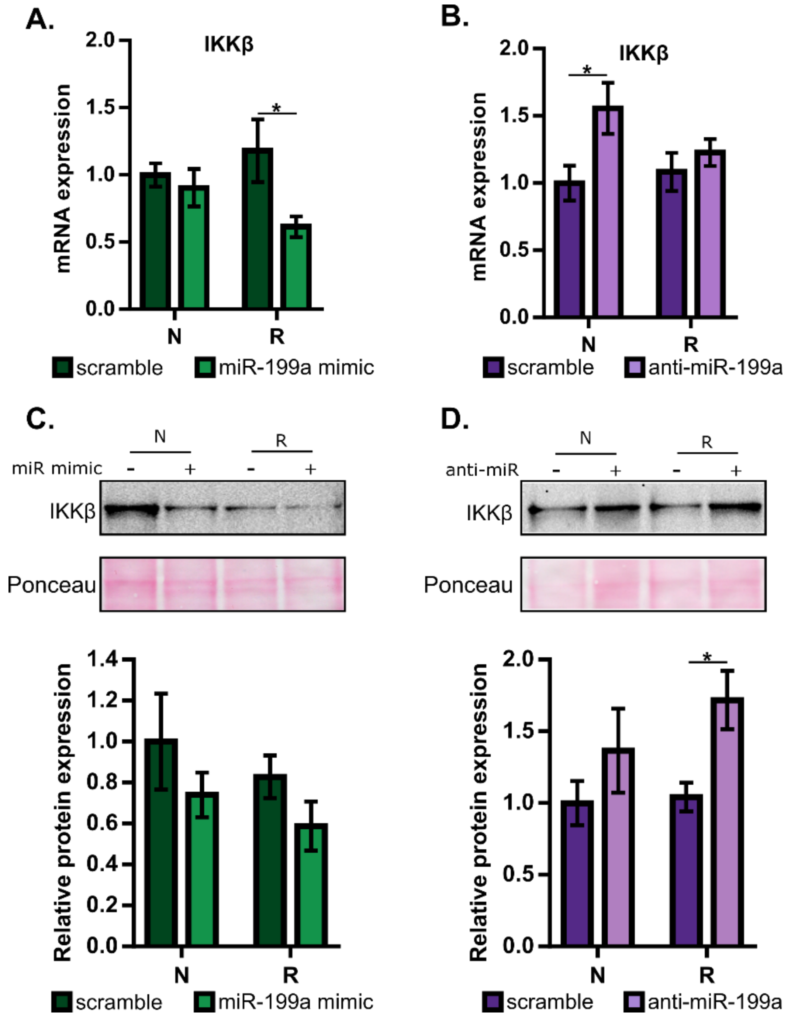

Figure S2. MicroRNA-199a modulation alters IKKβ expression .

A, B Bar graphs of relative IKKβ mRNA expression in transfected HUVECs subjected to non-reversed (N) or reversed (R) flow waveforms, 6-hr after simulated FAL. (n=4). *p<0.05, Student's *t*-test. **C**, **D** Bar graphs of relative IKKβ protein expression (MW~ 85kDa) expression in transfected HUVECs 6-hr after simulated FAL. (n=4). Ponceau staining was used for normalization of total protein. *p<0.05, Student's *t*-test. Data are mean ± SEM.

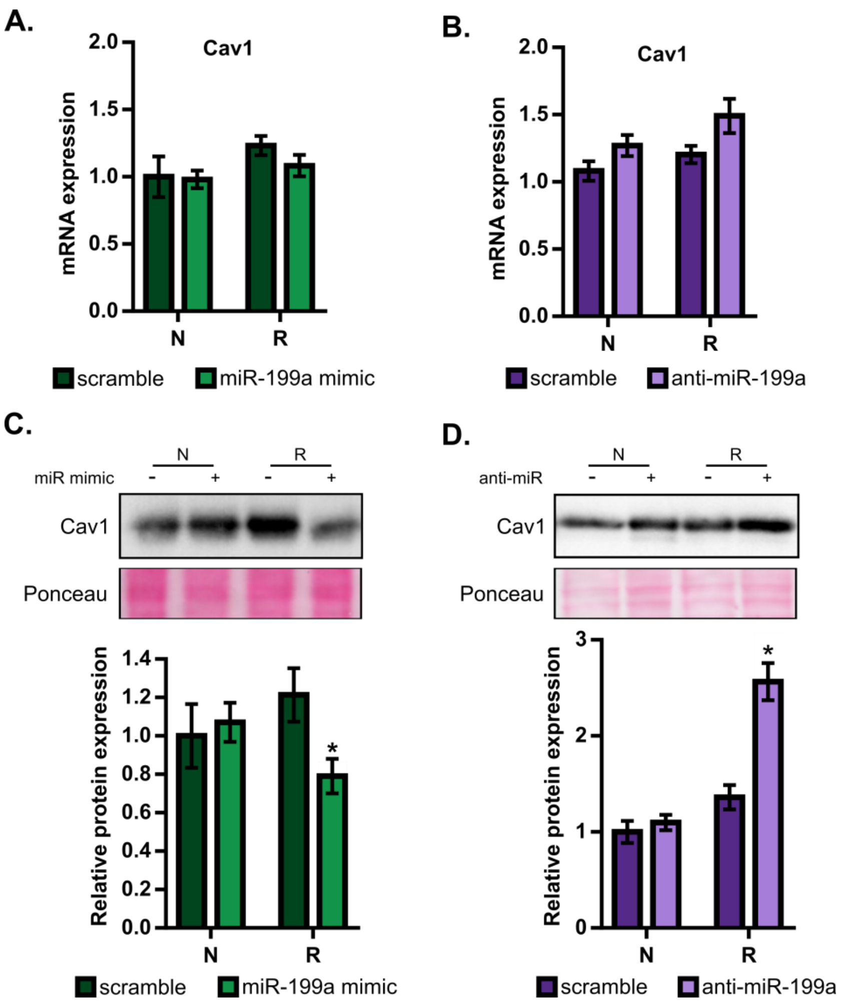

Figure S3. Altered miR-199a expression regulates Cav1 protein expression.
A, B Bar graphs of relative caveolin-1 (Cav1) mRNA expression in transfected HUVECs subjected to non-reversed (N) or reversed (R) flow waveforms, 6-hr after simulated FAL. (n=4). Student's *t*-test. **C**, **D** Bar graphs of relative Cav1 protein expression (MW~ 22kDa) expression in transfected HUVECs 6-hr after simulated FAL. (n=4). Ponceau staining was used for normalization of total protein. *p<0.05, Student's *t*-test. Data are mean ± SEM.

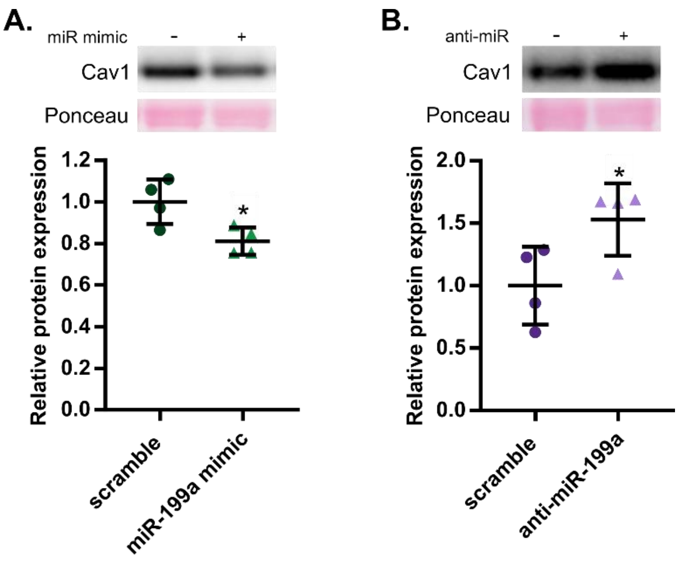

Figure S4. MicroRNA-199a negatively regulates the expression of caveolin-1 in-vivo.
A, B Bar graphs of relative caveolin-1 (Cav1, MW~22kDa) expression in the gracilis muscle 7 days post FAL in Balb/c mice injected with (A) miR-199a-5p mimic, (B) anti-miR-199a-5p, or the respective scramble control. (n=4). Ponceau staining was used for normalization of total protein.. *p<0.05, Student's *t*-test. Data are mean ± SEM.

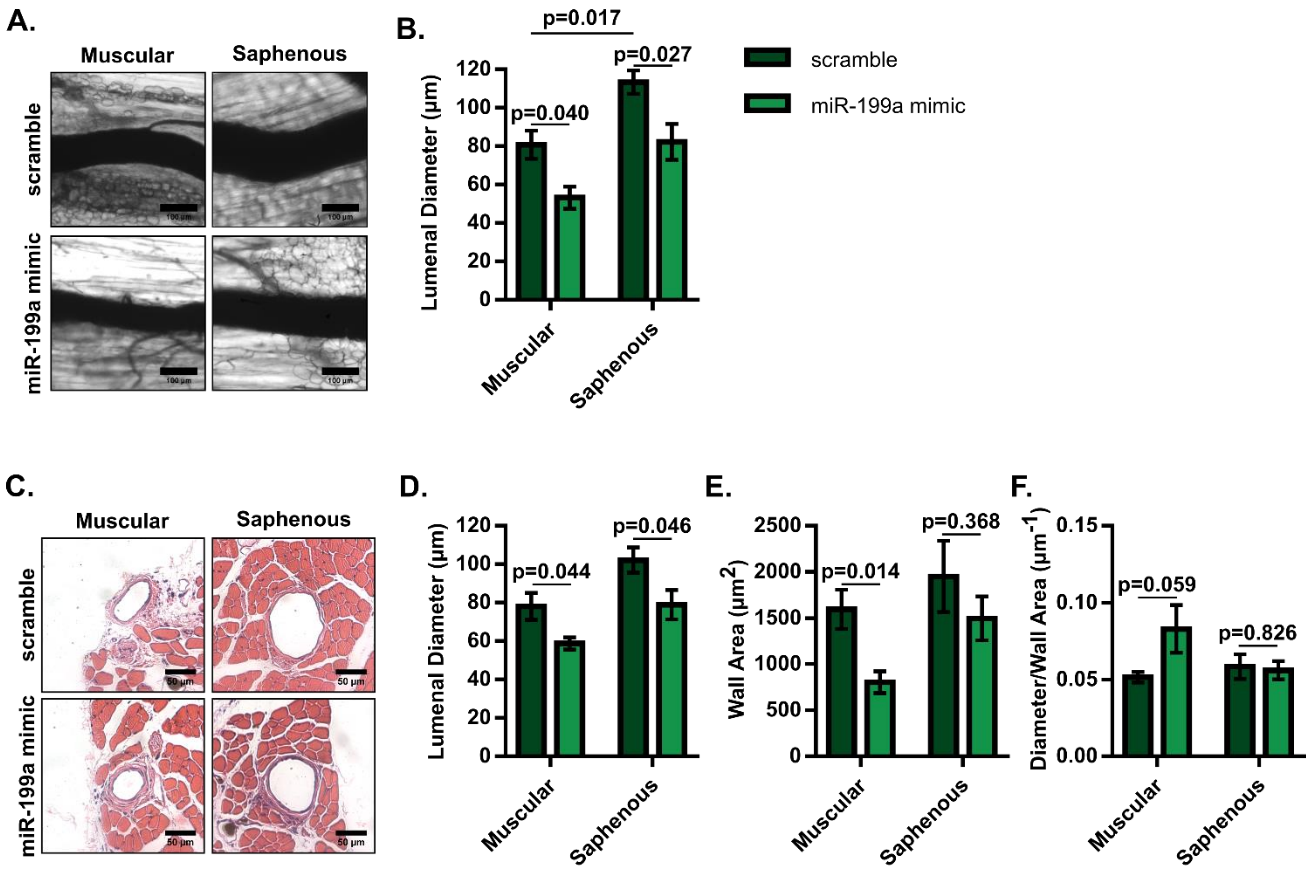

Figure S5. Regional analysis of Balb/c mice treated with miR-199a mimic after FAL.

A, Representative whole mount vascular cast images of collateral artery regions in scramble or miR-199a mimic treated Balb/c mice 21 days post-FAL (Scale bar = 100μm**)**. **B,** Bar graph of lumenal diameter for each group.*p<0.001 vs. unligated, two-way ANOVA followed by Holm-Sidak test for multiple comparisons. **C,** Representative H&E stained cross-sections of collateral arteries regions (n=5-6 for mimic and scramble groups respectively). **D-F,** Bar graphs of lumenal diameter, wall area, and diameter per wall area ratio from H&E stained cross-sections (n=5-6 for mimic and scramble groups respectively); Student's t-test. Data are mean ± SEM.

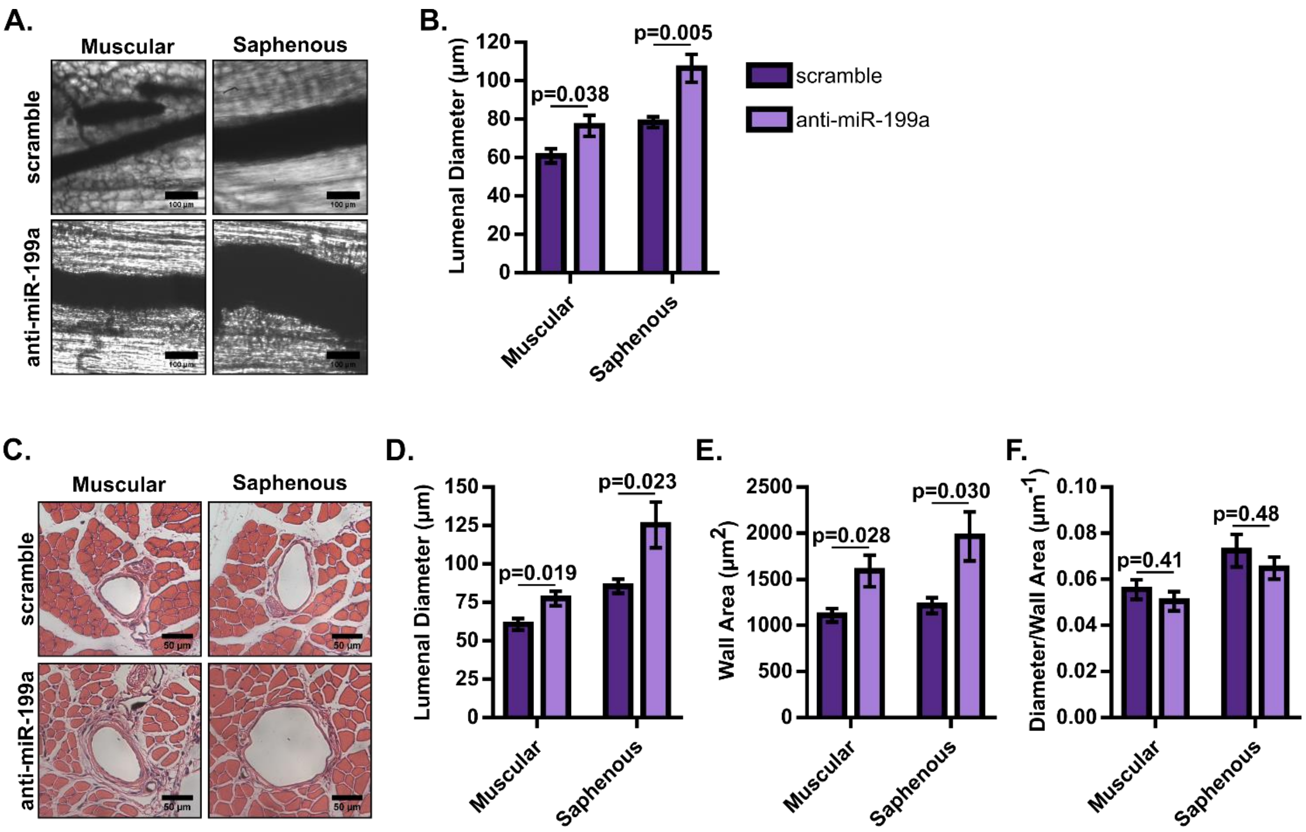

Figure S6. Regional analysis of Balb/c mice treated with anti-miR-199a after FAL.
A, Representative whole mount vascular cast images of collateral artery regions in scramble or anti-miR-199a treated Balb/c mice 21 days post-FAL (Scale bar=100μm). **B,** Bar graph of lumenal diameter for each group (n=6). *p<0.001 vs. unligated, two-way ANOVA followed by Holm-Sidak test for multiple comparisons. **C,** Representative H&E stained cross-sections of collateral arteries regions (n=6). **D-F,** Bar graphs of lumenal diameter, wall area, and diameter per wall area ratio from H&E stained cross-sections (n=6); Student’s t-test. Data are mean ± SEM.

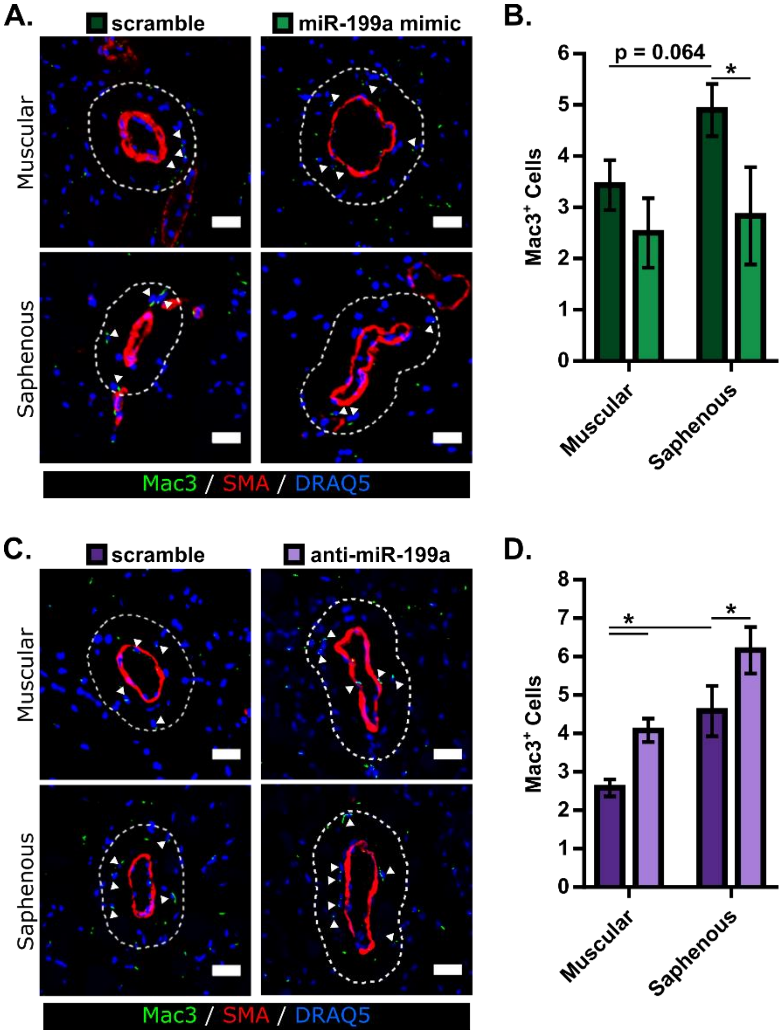

Figure S7. Regional pericollateral macrophage recruitment is modulated by miR-199a.
A, Representative cross-sections of gracilis collateral artery regions 7 days post-FAL immunolabeled for macrophage marker, Mac3 (green), smooth muscle alpha actin (SMA, red), and nuclei (DRAQ5, blue) in Balb/c mice treated with miR-199a mimic or scramble mimic. Dotted line indicates the pericollateral region (25μm from vessel wall) used for quantification. Arrowheads indicate Mac3^{+} cells (Scale bar=25μm). **B,** Bar graph of pericollateral Mac3^{+} cells (n=4-5 for miR-199a mimic and scramble, respectively). *p<0.05, two-way ANOVA followed by a Holm-Sidak test for multiple comparisons. **C,** Immunolabeled gracilis collateral artery regions, as in (A), in Balb/c mice treated with anti-miR-199a or scramble oligonucleotide. **D,** Bar graph of pericollateral Mac3^{+} cells (n=3). *p<0.05, two-way ANOVA followed by a Holm-Sidak test for multiple comparisons. Data are mean ± SEM.

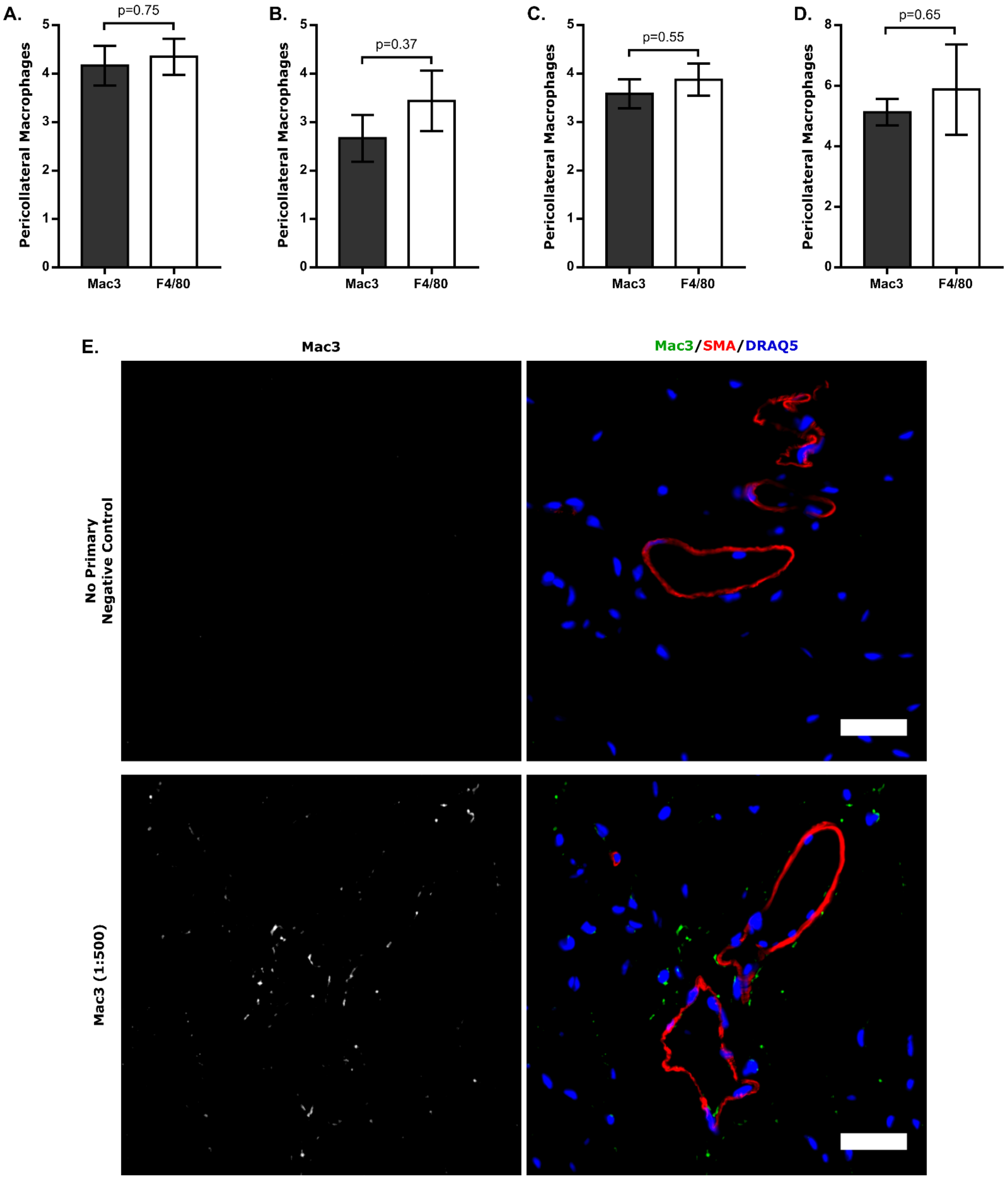

Figure S8. F4/80+ pericollateral macrophages exhibit similar trend as Mac3
A-D, Counts of Mac3⁺ or F4/80⁺ pericollateral macrophages in mice treated with (A) scramble mimic (n=5) (B) miR-199a mimic (n=4) (C) scramble anti-miR (n=3) or (D) anti-miR-199a (n=3) oligonucleotides, day 7 post-FAL. There is no statistical difference between Mac3 or F4/80 in any of the groups. Student's t-test. Data are mean ± SEM. **E,** Cross-sections of gracilis collateral artery regions 7 days post-FAL immunolabeled without primary antibody (negative control) or Mac3 (green, 1:500). Sections were counterstained with smooth muscle alpha actin (SMA, red) and DRAQ5 (nuclei, blue) (Scale bar=25μm).

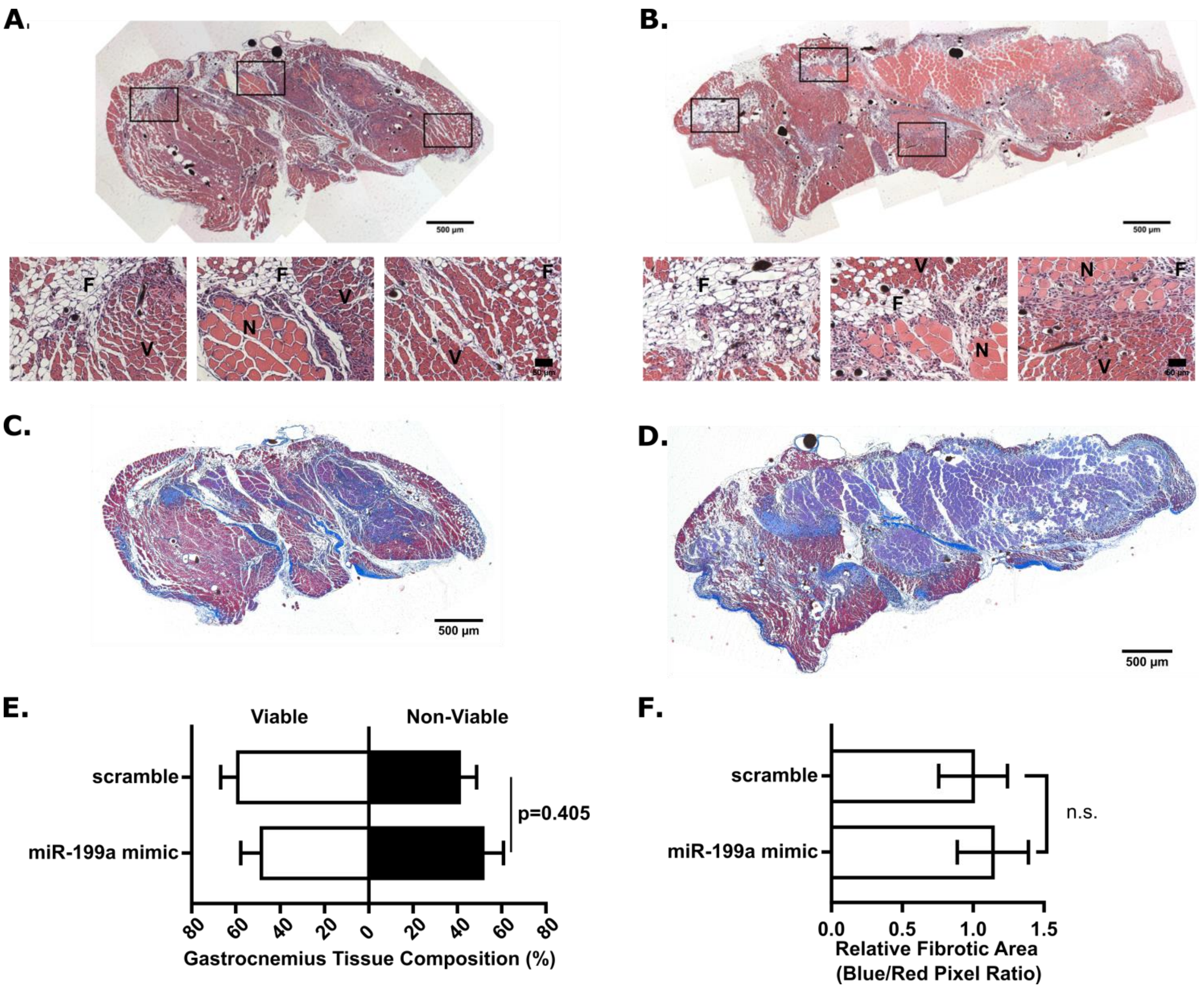

Figure S9. Gastrocnemius muscle composition is not further impaired with miR-199a overexpression following FAL

A-B, Representative images of H&E staining of gastrocnemius muscle for ligated limb of Balb/c mice treated with scramble (A) or miR-199a mimic (B) (Scale bar=500μm, inset scale bar=50μm). V=viable muscle, N=necrotic tissue, and F=fibro-adipose tissue. **C-D,** Representative images of Masson Trichrome stained gastrocnemius muscles from ligated limb of Balb/c mice treated with scramble (C) or miR-199a mimic (D) (Scale bar=500μm). Blue staining is indicative of collagen/fibrotic content whereas red staining indicates healthy tissue. **E,** Bar graph of the percentage of gastrocnemius muscle that is viable (white) or non-viable (black) at day 21 post-FAL in each group (n=6). **F,** Bar graph of the relative fibrotic area in gastrocnemius muscle at day 21 post-FAL in each group (n=6). n.s. = not significant ($p>0.05$), Student's *t*-test. Data are mean ± SEM.

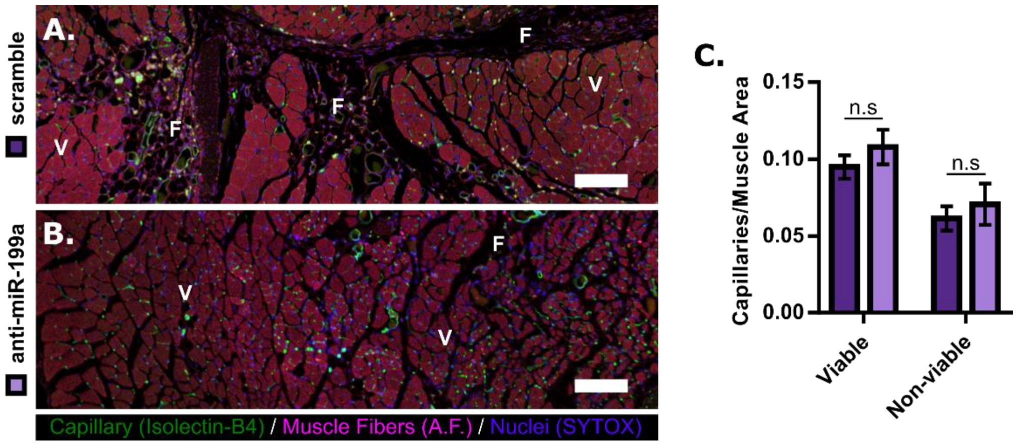

Figure S10. MicroRNA-199a inhibition does not affect capillary density in the gastrocnemius muscle of FAL-operated Balb/c mice.
A-B, Representative photomerged images of gastrocnemius muscle for ligated limb of Balb/c mice treated with scramble (A) or anti-miR-199a (B) locked nucleic acid oligonucleotides immediately after FAL (Scale bar=500μm). Tissues are immunolabeled for capillaries (green, isolectin-B4), muscle fibers (red, autofluorescence [A.F]), and nuclei (blue, SYTOX). **C,** Capillary density was determined for both groups in viable tissue (mature and regenerating muscle, V) and non-viable tissue (necrotic and fibro-adipose [F]) (n=4-5, for ctrl and anti-miR-199a, respectively). n.s = not significant, Student's t-test. Data are mean ± SEM.

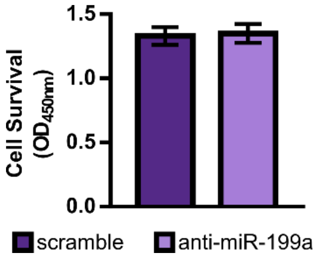

Figure S11. miR-199a inhibition does not improve C2C12 survival under hypoxic conditions in-vitro. Twenty-four hours after transfection with 30nM scramble or anti-miR-199a-5p oligonucleotides, C2C12 myoblasts were exposed to 6 hours of hypoxia (2% O2) and serum starvation conditions and assess for cell viability (n=4). Student's *t*-test. Data are mean ± SEM.

Table S1. *Differential microRNA gene expression in ECs exposed to arteriogenesis-biomimetic shear stress waveforms.*

Symbol	Name	NvC FC	RvC FC	RvN FC	$-log_{10}$(FDR)
MIR146A	microRNA 146a	0.99	0.52	0.52	2.00
MIR199A2	microRNA 199a-2	0.95	0.72	0.76	1.89
MIR34A	microRNA 34a	0.92	1.00	1.09	1.50
MIR100	microRNA 100	0.91	0.69	0.76	1.49
MIR216A	microRNA 216a	0.93	0.74	0.79	1.26
MIR93	microRNA 93	1.05	0.84	0.80	1.19
MIR17HG	miR-17-92 cluster host gene	1.01	0.81	0.80	0.97
MIR126	microRNA 126	0.85	0.70	0.82	0.87
MIR224	microRNA 224	1.07	0.73	0.68	0.87
MIR382	microRNA 382	0.95	1.22	1.28	0.87
MIR181B1	microRNA 181b-1	0.99	0.81	0.82	0.83
MIR155	microRNA 155	1.23	0.77	0.63	0.76
MIR145	microRNA 145	1.07	1.18	1.11	0.70
MIR103A1	microRNA 103a-1	0.87	0.90	1.04	0.70
MIR105-2	microRNA 105-2	1.12	1.23	1.10	0.69
MIR32	microRNA 32	1.00	0.76	0.76	0.64
MIR21	microRNA 21	1.05	0.77	0.73	0.59
MIR219-2	microRNA 219-2	0.97	1.10	1.13	0.58
MIR22HG	MIR22 host gene	1.07	1.12	1.05	0.58
MIR181B2	microRNA 181b-2	0.96	0.84	0.87	0.57
MIR30E	microRNA 30e	0.93	0.88	0.94	0.56
MIR485	microRNA 485	0.99	1.12	1.13	0.55
MIR127	microRNA 127	0.96	0.90	0.93	0.54
MIR10A	microRNA 10a	0.81	0.78	0.97	0.51
MIRLET7C	microRNA let-7c	0.96	0.82	0.85	0.51
MIR429	microRNA 429	1.01	1.11	1.11	0.50
MIR23A	microRNA 23a	1.02	0.84	0.83	0.48
MIR199A1	microRNA 199a-1	1.12	0.89	0.79	0.45
MIR106B	microRNA 106b	0.98	0.91	0.94	0.41
MIRLET7I	microRNA let-7i	1.03	1.11	1.08	0.39
MIR133A1	microRNA 133a-1	0.97	1.11	1.14	0.38
MIR29A	microRNA 29a	1.13	0.78	0.69	0.37
MIR27A	microRNA 27a	0.85	0.85	1.01	0.37
MIR622	microRNA 622	0.90	1.21	1.36	0.36
MIR29B2	microRNA 29b-2	1.07	0.86	0.81	0.35
MIR143	microRNA 143	1.05	0.92	0.88	0.35
MIR30C1	microRNA 30c-1	1.00	0.89	0.90	0.32
MIR9-2	microRNA 9-2	1.00	1.07	1.07	0.31
MIRLET7F1	microRNA let-7f-1	1.07	1.16	1.09	0.30
MIR24-2	microRNA 24-2	0.93	0.88	0.95	0.29
MIR154	microRNA 154	0.97	1.24	1.27	0.28
MIR26A1	microRNA 26a-1	0.94	0.90	0.96	0.26
MIR25	microRNA 25	0.85	0.91	1.08	0.26
MIR15B	microRNA 15b	0.77	0.91	1.18	0.25
MIR320C1	microRNA 320c-1	1.11	1.08	0.97	0.25
MIR124-2	microRNA 124-2	1.00	0.95	0.95	0.25

MIR214	microRNA 214	0.93	0.93	1.00	0.24
MIR206	microRNA 206	1.04	1.06	1.02	0.24
MIR186	microRNA 186	0.94	1.11	1.18	0.24
MIR365A	microRNA 365a	0.78	0.91	1.18	0.24
MIR34C	microRNA 34c	0.86	0.93	1.08	0.24
MIR181A1	microRNA 181a-1	0.99	0.92	0.93	0.23
MIR195	microRNA 195	1.06	0.93	0.88	0.22
MIR221	microRNA 221	0.95	1.14	1.21	0.22
MIR99B	microRNA 99b	0.96	0.93	0.96	0.21
MIR122	microRNA 122	1.09	1.07	0.97	0.21
MIR377	microRNA 377	0.98	1.07	1.10	0.20
MIR137HG	MIR137 host gene	1.00	1.08	1.08	0.20
MIR194-1	microRNA 194-1	0.92	1.10	1.20	0.20
MIR24-1	microRNA 24-1	1.14	0.90	0.80	0.19
MIR210	microRNA 210	1.07	0.95	0.89	0.18
MIR184	microRNA 184	0.99	0.94	0.94	0.17
MIR105-1	microRNA 105-1	1.24	1.24	0.99	0.17
MIR520C	microRNA 520c	0.99	0.91	0.91	0.16
MIR15A	microRNA 15a	0.80	0.87	1.10	0.16
MIR23B	microRNA 23b	0.85	0.91	1.07	0.16
MIR376C	microRNA 376c	0.92	1.05	1.15	0.16
MIR218-1	microRNA 218-1	0.85	0.91	1.07	0.16
MIR492	microRNA 492	1.03	1.08	1.05	0.15
MIR101-2	microRNA 101-2	1.03	1.05	1.02	0.15
MIR28	microRNA 28	1.01	1.09	1.08	0.14
MIR200A	microRNA 200a	0.98	1.08	1.10	0.14
MIR320A	microRNA 320a	0.92	1.06	1.15	0.14
MIR323A	microRNA 323a	1.08	1.06	0.98	0.14
MIR147A	microRNA 147a	0.71	0.94	1.33	0.14
MIR26B	microRNA 26b	0.94	0.96	1.01	0.13
MIR16-1	microRNA 16-1	0.82	0.96	1.17	0.13
MIRLET7A1	microRNA let-7a-1	1.01	0.93	0.93	0.13
MIR29C	microRNA 29c	1.20	1.10	0.92	0.13
MIR136	microRNA 136	1.03	1.08	1.04	0.12
MIR135A1	microRNA 135a-1	1.00	0.93	0.94	0.12
MIR7-3HG	MIR7-3 host gene	0.89	0.95	1.06	0.12
MIR219-1	microRNA 219-1	0.96	1.07	1.12	0.11
MIR208A	microRNA 208a	0.97	1.06	1.09	0.11
MIR153-1	microRNA 153-1	1.01	0.95	0.94	0.11
MIR183	microRNA 183	0.98	0.95	0.97	0.11
MIR199B	microRNA 199b	0.87	0.93	1.06	0.11
MIR519A2	microRNA 519a-2	1.00	0.96	0.96	0.10
MIR99A	microRNA 99a	0.93	0.95	1.02	0.10
MIR7-2	microRNA 7-2	0.61	1.04	1.71	0.10
MIRLET7A3	microRNA let-7a-3	1.02	0.93	0.91	0.10
MIR217	microRNA 217	0.98	0.88	0.90	0.10
MIR144	microRNA 144	0.91	1.05	1.16	0.10
MIR139	microRNA 139	1.02	1.04	1.02	0.09
MIRLET7F2	microRNA let-7f-2	1.00	1.08	1.09	0.09
MIR410	microRNA 410	0.96	0.95	0.99	0.09

MIR211	microRNA 211	0.77	0.94	1.23	0.09
MIR185	microRNA 185	0.98	0.96	0.98	0.09
MIR412	microRNA 412	1.01	1.05	1.04	0.09
MIR197	microRNA 197	0.93	0.95	1.02	0.09
MIR188	microRNA 188	1.08	1.03	0.95	0.09
MIR33A	microRNA 33a	0.93	0.93	1.00	0.09
MIR519A2	microRNA 519a-2	1.00	0.92	0.92	0.08
MIR302A	microRNA 302a	0.94	0.97	1.02	0.08
MIR30D	microRNA 30d	0.95	1.04	1.10	0.08
MIRLET7E	microRNA let-7e	0.89	0.93	1.04	0.08
MIR96	microRNA 96	0.98	1.05	1.07	0.08
MIR16-2	microRNA 16-2	0.95	0.96	1.01	0.08
MIR181A2	microRNA 181a-2	0.86	0.93	1.09	0.08
MIR134	microRNA 134	1.00	1.04	1.04	0.07
MIR495	microRNA 495	1.16	0.96	0.83	0.07
MIR516B2	microRNA 516b-2	0.97	0.94	0.97	0.07
MIR30A	microRNA 30a	0.91	1.06	1.16	0.07
MIRLET7D	microRNA let-7d	0.94	1.04	1.11	0.07
MIR223	microRNA 223	1.27	0.97	0.77	0.07
MIR30C2	microRNA 30c-2	0.99	0.92	0.93	0.07
MIR124-1	microRNA 124-1	1.04	1.05	1.00	0.07
MIR193A	microRNA 193a	0.96	1.04	1.08	0.07
MIR137	microRNA 137	1.18	1.04	0.88	0.07
MIR600HG	MIR600 host gene	1.14	0.96	0.85	0.07
MIR218-2	microRNA 218-2	0.88	0.95	1.09	0.07
MIR9-3	microRNA 9-3	0.91	0.96	1.05	0.07
MIR187	microRNA 187	0.96	0.97	1.00	0.06
MIR202	microRNA 202	1.00	1.04	1.04	0.06
MIR1-1	microRNA 1-1	0.98	1.03	1.06	0.06
MIR203	microRNA 203	0.94	1.03	1.09	0.06
MIR339	microRNA 339	1.03	1.04	1.01	0.06
MIRLET7B	microRNA let-7b	1.00	0.97	0.97	0.06
MIR509-1	microRNA 509-1	1.02	1.03	1.01	0.06
MIR103A2	microRNA 103a-2	0.87	1.05	1.21	0.06
MIR26A2	microRNA 26a-2	1.04	1.06	1.01	0.06
MIR487A	microRNA 487a	1.12	1.04	0.93	0.06
MIR301A	microRNA 301a	1.09	0.96	0.89	0.06
MIR124-3	microRNA 124-3	1.03	0.95	0.93	0.06
MIR140	microRNA 140	0.98	0.97	0.98	0.06
MIR451A	microRNA 451a	0.98	1.03	1.05	0.06
MIR128-1	microRNA 128-1	0.98	1.02	1.05	0.06
MIR182	microRNA 182	0.92	0.96	1.05	0.06
MIR212	microRNA 212	0.90	0.97	1.07	0.06
MIR148A	microRNA 148a	0.92	1.03	1.12	0.05
MIR153-2	microRNA 153-2	1.01	0.98	0.97	0.05
MIR1247	microRNA 1247	0.95	0.98	1.03	0.05
MIR130B	microRNA 130b	1.00	1.03	1.03	0.05
MIR503	microRNA 503	1.07	0.97	0.91	0.05
MIR7-3	microRNA 7-3	0.97	0.97	1.00	0.05
MIR331	microRNA 331	1.03	1.04	1.01	0.05

MIR204	microRNA 204	0.96	1.03	1.08	0.04
MIR125A	microRNA 125a	0.90	0.97	1.07	0.04
MIR129-2	microRNA 129-2	1.11	0.97	0.87	0.04
MIR34B	microRNA 34b	0.94	1.03	1.10	0.04
MIR504	microRNA 504	1.20	1.03	0.86	0.04
MIR190A	microRNA 190a	0.90	1.02	1.14	0.04
MIRLET7A2	microRNA let-7a-2	1.03	0.96	0.93	0.04
MIR425	microRNA 425	1.28	0.97	0.76	0.03
MIR132	microRNA 132	0.92	0.97	1.06	0.03
MIR192	microRNA 192	1.06	1.03	0.97	0.03
MIR95	microRNA 95	0.97	1.02	1.05	0.03
MIR133B	microRNA 133b	1.04	1.02	0.98	0.03
MIR148B	microRNA 148b	1.05	0.97	0.92	0.03
MIR455	microRNA 455	0.95	0.98	1.04	0.03
MIRLET7BHG	MIRLET7B host gene	1.01	0.98	0.97	0.03
MIR215	microRNA 215	0.94	1.02	1.08	0.03
MIR194-2	microRNA 194-2	0.98	1.02	1.04	0.03
MIR19B2	microRNA 19b-2	0.72	1.02	1.42	0.03
MIR92A2	microRNA 92a-2	1.12	0.99	0.88	0.03
MIR30B	microRNA 30b	1.06	1.03	0.97	0.03
MIR27B	microRNA 27b	0.94	1.03	1.10	0.02
MIR130A	microRNA 130a	0.98	1.02	1.04	0.02
MIR149	microRNA 149	0.89	1.02	1.14	0.02
MIR138-1	microRNA 138-1	1.05	1.02	0.97	0.02
MIR135A2	microRNA 135a-2	0.96	0.98	1.03	0.02
MIR1-2	microRNA 1-2	1.06	0.98	0.93	0.02
MIR141	microRNA 141	1.01	0.98	0.97	0.02
MIR196A2	microRNA 196a-2	0.98	1.01	1.04	0.02
MIR423	microRNA 423	1.04	0.98	0.94	0.02
MIR516B1	microRNA 516b-1	1.06	0.98	0.92	0.02
MIR191	microRNA 191	1.01	0.99	0.97	0.02
MIR222	microRNA 222	1.03	0.98	0.95	0.02
MIR375	microRNA 375	0.96	1.01	1.06	0.01
MIR181C	microRNA 181c	1.10	1.01	0.92	0.01
MIR9-1	microRNA 9-1	1.02	1.01	0.99	0.01
MIR31HG	MIR31 host gene	0.99	1.01	1.02	0.01
MIRLET7G	microRNA let-7g	0.91	0.98	1.08	0.01
MIR196A1	microRNA 196a-1	0.93	1.01	1.09	0.01
MIR129-1	microRNA 129-1	0.97	0.99	1.02	0.01
MIR125B2	microRNA 125b-2	1.08	0.99	0.92	0.01
MIR31	microRNA 31	1.03	1.02	0.99	0.01
MIR326	microRNA 326	0.85	1.01	1.19	0.01
MIR296	microRNA 296	1.00	1.01	1.01	0.01
MIR142	microRNA 142	0.96	1.01	1.05	0.01
MIR138-2	microRNA 138-2	0.93	1.01	1.08	0.01
MIR152	microRNA 152	0.98	1.01	1.03	0.01
MIR125B1	microRNA 125b-1	0.97	0.99	1.02	0.01
MIR106A	microRNA 106a	0.99	1.01	1.02	0.01
MIR10B	microRNA 10b	1.01	1.01	1.00	0.01
MIR101-1	microRNA 101-1	0.89	0.99	1.12	0.01

MIR330	microRNA 330	1.01	1.01	1.00	0.01
MIR302B	microRNA 302b	1.11	1.01	0.91	0.01
MIR200B	microRNA 200b	1.03	1.00	0.97	0.01
MIR150	microRNA 150	0.99	1.01	1.01	0.01
MIR200C	microRNA 200c	0.99	1.01	1.01	0.01
MIR128-2	microRNA 128-2	0.97	0.99	1.03	0.00
MIR34A	microRNA 34a	0.99	0.72	0.73	0.00
MIR98	microRNA 98	1.05	1.00	0.96	0.00
MIR29B1	microRNA 29b-1	0.91	1.00	1.10	0.00
MIR299	microRNA 299	0.91	1.00	1.11	0.00
MIR107	microRNA 107	1.02	1.00	0.99	0.00
MIR494	microRNA 494	1.00	1.00	1.00	0.00
MIR205	microRNA 205	0.99	1.00	1.01	0.00

*FC = fold change. FDR = false discovery rate of RvC dataset.
N = non-reversed, R = reversed, C= control shear stress waveforms from Heuslein and Meisner et al.

Table S2. Patient demographics of human plasma samples.

Patient Information	Control (n=25)	PAD (n=25)	p-value
Minimum ABI	1.09 ± 0.12	0.75 ± 0.26	<0.0001
Male / Female	60% / 40%	52% / 48%	0.776
Age (years)	64.2 ± 7.7	64.8 ± 11.3	0.977
Smoker	68%	92%	0.074
Diabetic	40%	36%	0.999
Hypertensive	88%	96%	0.609
Hyperlipidemic	80%	85%	0.702

Data are mean ± SD. A Mann-Whitney U-test was used for minimum ankle-brachial index (ABI) and age data whereas categorical data was subjected to a Fisher's exact test to determine statistical differences between groups. $p<0.05$ is considered statistically significant.
